# Mechanistic insights into the reductive dehydroxylation pathway for the biosynthesis of isoprenoids promoted by the IspH enzyme†
†Electronic supplementary information (ESI) available: Computational methods, further analysis, and Cartesian coordinates of all the species discussed. See DOI: 10.1039/c5sc01693b



**DOI:** 10.1039/c5sc01693b

**Published:** 2015-06-22

**Authors:** Safwat Abdel-Azeim, Abdesslem Jedidi, Jorg Eppinger, Luigi Cavallo

**Affiliations:** a King Abdullah University of Science and Technology , KAUST Catalysis Research Center , Physical Sciences and Engineering Division , Thuwal 23955-6900 , Saudi Arabia . Email: luigi.cavallo@kaust.edu.sa

## Abstract

Here, we report an integrated quantum mechanics/molecular mechanics (QM/MM) study of the bio-organometallic reaction pathway of the 2H^+^/2e^–^ reduction of (*E*)-4-hydroxy-3-methylbut-2-enyl pyrophosphate (HMBPP) into the so called universal terpenoid precursors isopentenyl pyrophosphate (IPP) and dimethylallyl pyrophosphate (DMAPP), promoted by the IspH enzyme. Our results support the viability of the bio-organometallic pathway through rotation of the OH group of HMBPP away from the [Fe_4_S_4_] cluster at the core of the catalytic site, to become engaged in a H-bond with Glu126. This rotation is synchronous with π-coordination of the C2=C3 double bond of HMBPP to the apical Fe atom of the [Fe_4_S_4_] cluster. Dehydroxylation of HMBPP is triggered by a proton transfer from Glu126 to the OH group of HMBPP. The reaction pathway is completed by competitive proton transfer from the terminal phosphate group to the C2 or C4 atom of HMBPP.

## Abstract 2

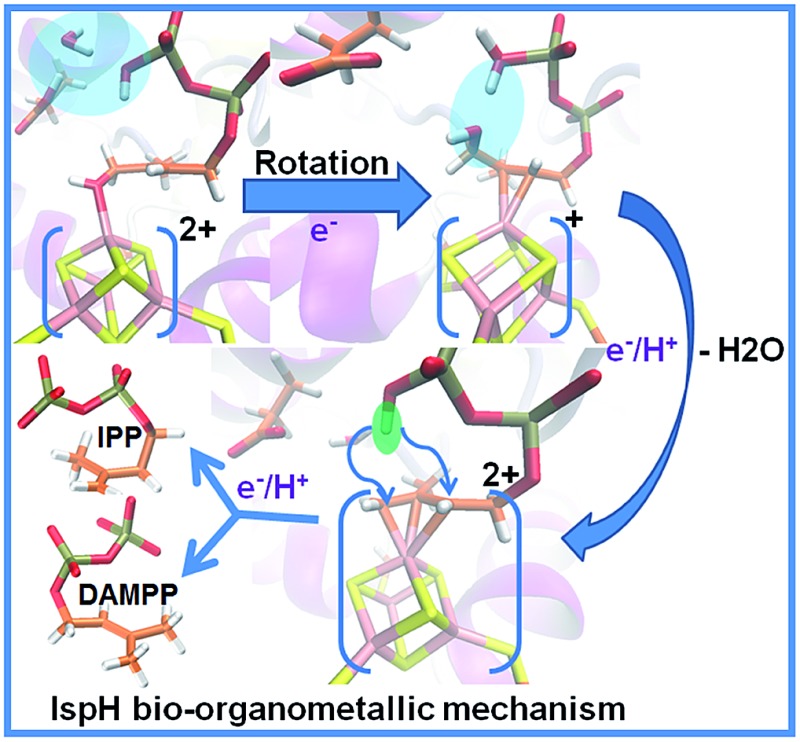
We report an integrated QM/MM study of the bio-organometallic reaction pathway of the reductive dehydroxylation of (*E*)-4-hydroxy-3-methylbut-2-enyl pyrophosphate (HMBPP).

## Introduction

Malaria and tuberculosis are plagues threatening the health of millions of humans every year.^1,2^ This has spurred intense research programs targeting the development of drugs against these diseases. One of the most promising strategies is focused on blocking the biosynthesis of isoprenoids in the pathogenic bacteria. This strategy is possible because there are two main pathways for the biosynthesis of isoprenoids. The first is the non-mevalonate pathway, known as the methylerythritol phosphate (MEP) pathway, and the second is the mevalonate (MVA) pathway.^3^ The MEP pathway is operative in the overwhelming majority of eubacteria, including key pathogens, while the MVA pathway is operative in archaebacteria, most eukaryotes and fungi. Thus, blocking the MEP pathway would allow the inhibition of isoprenoid biosynthesis in pathogenic bacteria without affecting their biosynthesis in humans.

**Scheme 1:**
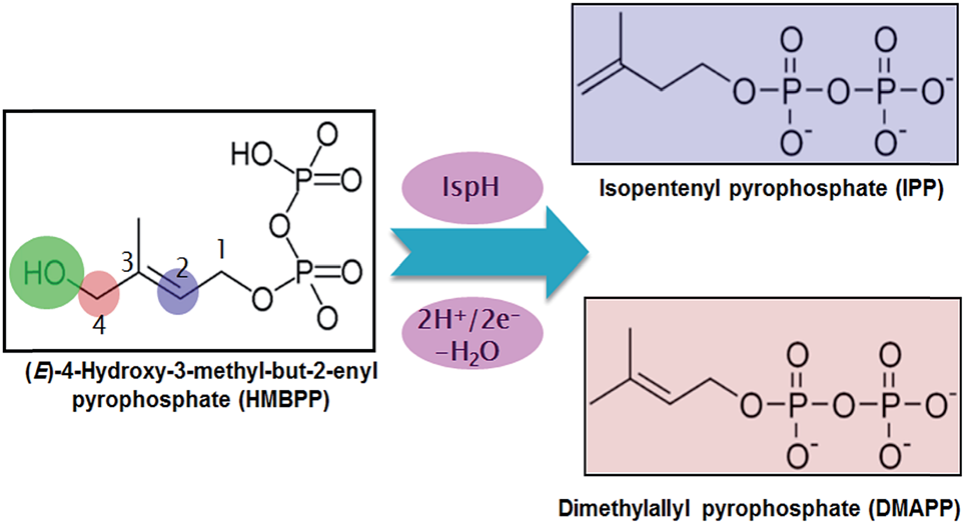
Conversion of HMBPP into IPP and DMAPP by IspH.

In this scenario, the greatest attention has been focused on two enzymes at the end of the sequential cascade of the MEP pathway, namely the IspG and IspH enzymes. Both enzymes use a [Fe_4_S_4_] iron–sulfur cluster as a cofactor. The IspH enzyme, the focus of this work, is functional in the 2H^+^/2e^–^ reduction of (*E*)-4-hydroxy-3-methylbut-2-enyl pyrophosphate (HMBPP) into the so called universal terpenoid precursors isopentenyl pyrophosphate (IPP) and dimethylallyl pyrophosphate (DMAPP), see Scheme 1.

**Fig. 1:**
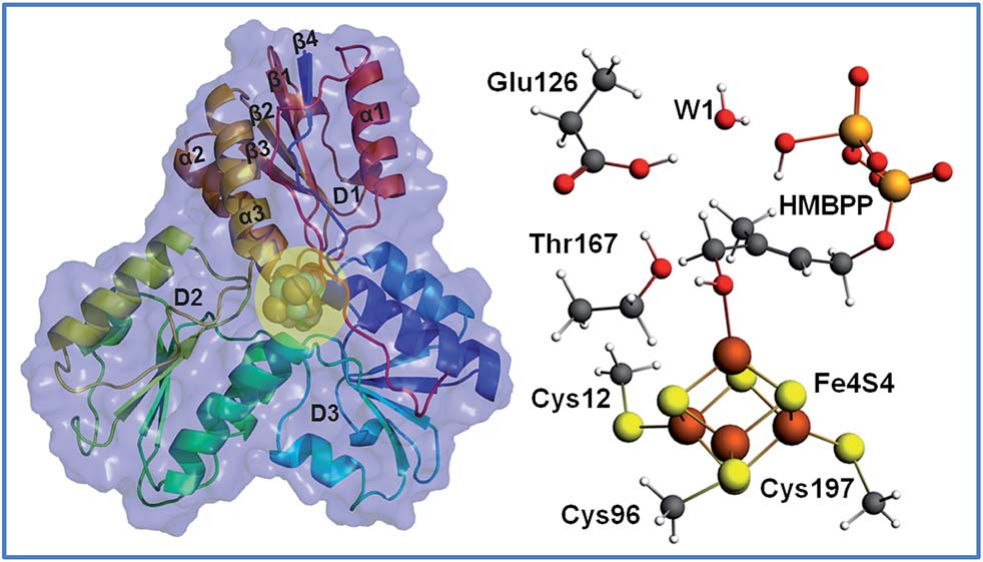
The IspH secondary structure, with a sphere representation of the iron–sulfur cluster and the HMBPP substrate (highlighted in the yellow circle), and the stick and ball views of the iron–sulfur cluster, coordinated cysteinates, HMBPP, Thr167, Glu126 and a bridging water molecule.

**Fig. 2:**
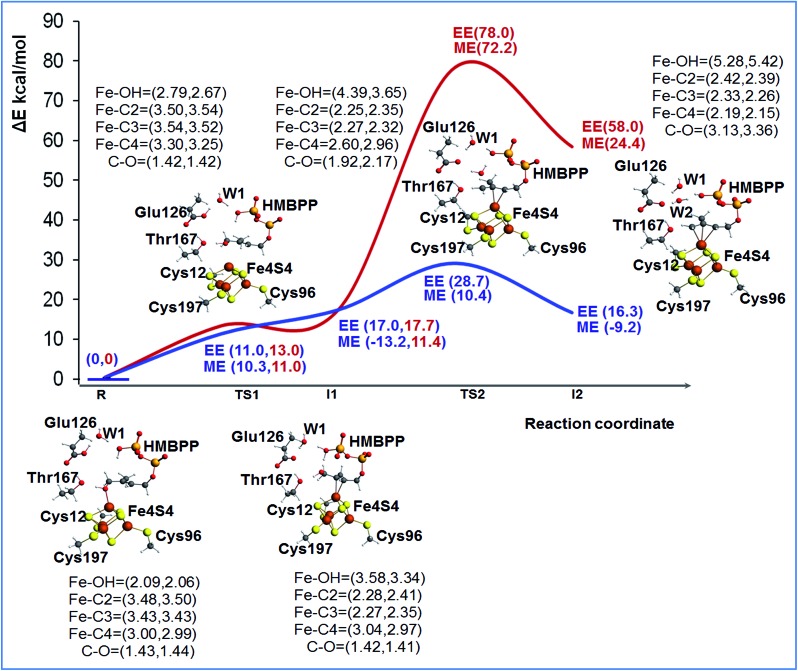
The ONIOM (B3LYP/TZVP:Amber) reaction profile of the first step in the IspH promoted reactivity, corresponding to rotation and dehydroxylation. The red line corresponds to the case of deprotonated Glu126, while the blue line corresponds to the case of protonated Glu126. ME energies correspond to mechanical embedding with model M1, which is the mode used in the geometry optimization. EE energies correspond to electrostatic embedding evaluated through single point energy calculations with model M2 using geometries from model M1. Stationary points are represented as balls and sticks. Key bond distances are reported in parenthesis, the first value is for the profile of deprotonated Glu126 and the second value is for the profile of protonated Glu126.

The structure of IspH consists of three domains with similar folding and it adopts an overall clover-like shape. The [Fe_4_S_4_] cofactor is located inside the central cavity of the enzyme (see Fig. 1). Each domain comprises four strands (β1–β4) arranged in a central parallel β sheet surrounded by α-helices (α1–α3). The cluster is coordinated by the highly conserved cysteines 12, 96 and 197, whose presence is fundamental in preserving enzymatic functionality, as evidenced by mutagenesis experiments.^4^ The crystallographic structure of IspH complexed with HMBPP reveals the hairpin conformation of the ligand, with the hydroxyl group bound to the apical iron atom Fe1 of the [Fe_4_S_4_]^2+^ cluster (for atom numbering see Fig. 2).^5^

**Scheme 2:**
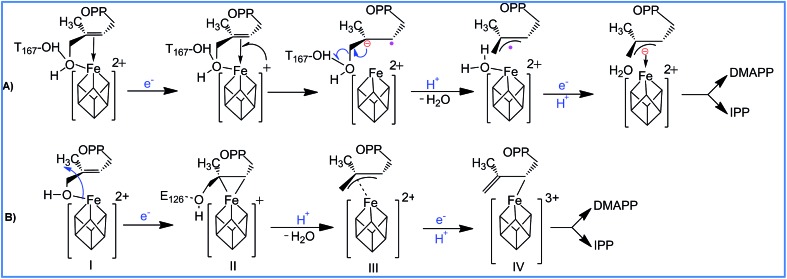
Reaction pathways and intermediates proposed to be involved in the IspH catalysis. (A) is the Birch-like mechanism, and (B) is the most accepted bio-organometallic mechanism.

With regards to the sequence of elementary steps composing the reaction pathway for HMBPP reduction by the IspH enzyme, a number of studies have converged on two distinct mechanisms. The so called Birch-like reduction pathway, and the bio-organometallic pathway, see Scheme 2. In the Birch-like reduction pathway, one electron is transferred from the iron–sulfur cluster to HMBPP, which prompts the rupture of the C–O bond. This converts the C-skeleton of HMBPP into an allyl radical intermediate, which is coordinated to the [Fe_4_S_4_] cluster. A second electron transfer, coupled with protonation at different carbons of the allyl moiety (C2 or C4 of HMBPP), gives IPP or DMAPP, respectively, see Scheme 2A.^6–9^

The most likely bio-organometallic pathway starts with dissociation and rotation of the OH group of HMBPP away from the apical Fe1 atom, to become engaged in a H-bond with the universally conserved Glu126 residue. Synchronous to this rotation, is a small slippage of the C2=C3 double bond of HMBPP to π-coordinate to the Fe1 atom, see Scheme 2B. This structural rearrangement triggers dehydroxylation of HMBPP, assisted by a proton transfer from Glu126, and conversion of the C-skeleton of HMBPP into an allyl moiety π-coordinated to the Fe1 atom. Then, a second electron transfer associated with protonation of the allyl intermediate to form IPP or DMAPP completes the reaction pathway.^10–12^

According to the bio-organometallic pathway, rotation of the 4-OH group to engage in a H-bond with Glu126 and π-coordination of the C2=C3 bond to the [Fe_4_S_4_] cluster are fundamental.^5^ Support for this proposal came from mutagenesis experiments, which demonstrated that replacing Glu126 by a glutamine reduces the enzymatic activity to a negligible 0.3%.^11,13^ Incidentally, this also suggested that besides maintaining the hydrogen bond network around the catalytic site, Glu126 also plays an important catalytic role.

In addition to the aforementioned evidence, electron paramagnetic resonance spectroscopy and crystallographic studies identified paramagnetic reaction intermediates using Glu126Ala and Glu126Gln mutants. These mutants cause the OH group of HMBPP to be rotated away from the Fe1 atom of the [Fe_4_S_4_] cluster, and a weakened π-complex between the Fe1 atom and the C2=C3 double bond of HMBPP, intermediate II in Scheme 2B. Furthermore, similar studies on the wild-type IspH allowed the characterization of intermediate IV in Scheme 2B. Finally, free radical formation was not detected during the reaction steps, which was taken as evidence against the occurrence of the Birch reduction mechanism.^14^

Despite the available data converging in favor of the bio-organometallic mechanism, a clear understanding of the elementary steps characterizing this mechanism are still missing. To shed light on these points, which would further support the viability of the bio-organometallic mechanism, we performed N-layered integrated molecular orbital and molecular mechanics (ONIOM) calculations.^15–17^

This approach has already proved reliable for characterization of the electronic properties of large biological systems.^18–21^ The modelling of metallo-enzymes containing magnetic molecules like the iron–sulfur cluster in IspH presents a further challenge due to the presence of the antiferromagnetic coupling between the iron atoms. This kind of coupling can be well described using the broken symmetry (BS) approach developed by Noodleman,^22,23^ and we have adopted it here. Indeed, the BS approach has been successfully used to study the electronic properties of iron–sulfur clusters.^24–29^ Finally, to investigate the convergence of the calculations with respect to the size of the quantum mechanics model used to simulate the active site, we performed DFT+U calculations on an extended model of up to 456 atoms.

## Results and discussion

### The optimized structure of the HMBPP-coordinated complex

In the crystallographic structure the Fe–S distances vary significantly, with the apical Fe1 atom showing the longest Fe–S bond lengths, with an average Fe1–S distance of 2.38 Å, whereas the Fe2 atom displays significantly shorter Fe–S bond lengths, with an average Fe2–S distance of 2.20 Å, consistent with the ferric character of the Fe2 atom. The Fe3–S and Fe4–S bond lengths assume intermediate values, with a mean Fe–S distance of 2.28 ± 0.04 Å, suggesting mixed valence for these atoms. An extensive list of experimental distances compared with the analogous distances in the ONIOM optimized (B3LYP/TZVP:Amber) structures using model M1 for the HBBPP-coordinated IspH in the oxidized [Fe_4_S_4_]^2+^ state in different BS states is reported in Table S1.†

An analysis of the structures optimized using different spin couplings between the Fe centers indicates that, on average, the DFT Fe–S distances are elongated compared to the corresponding distances in the crystallographic structure, with the exception of the Fe1–S distance, which is instead reproduced accurately. The general trend we observed is that longer Fe–S bond lengths were obtained for Fe1 and Fe4, with an average value of 2.38 Å, compared to Fe2 and Fe3, with an average value of 2.33 Å. Nevertheless, as indicated by Blachly *et al.*,^30^ the main conclusion is that the DFT optimized structures are unable to reproduce the asymmetry observed experimentally, with all the Fe–S distances in the restricted 2.33–2.38 Å range. As a final remark, we noticed that the optimized Fe–S_Cys_ bonds are generally in better agreement with the X-ray values, compared to the Fe–S–Fe bonds, which is not surprising considering that the Fe–S–Fe bonds have to mediate spin coupling between the Fe centers.

Moving to the Fe–Fe bond distances, all of them are elongated in comparison to the experimental distances. Nevertheless, considering these are distances between atoms that are not directly bonded, the agreement of the optimized structure with the X-ray structure is satisfactory. Indeed, focusing on the most stable BS state, the largest deviation between the DFT and crystallographic distances amounts to only 0.20 Å for the Fe2–Fe3 distance, which is reasonably small and definitely much smaller than the deviation reported for the Fe–Fe distance in similar systems.^31,32^

To check further for the impact of the computational protocol on the optimized geometries, test calculations were performed by adopting the ONIOM-EE scheme in the geometry optimization. The minimal deviation between the ONIOM-ME and ONIOM-EE geometries, with the Fe–S and Fe–Fe distances differing by less than 0.02 and 0.05 Å (see Table S3†), clearly indicates that the protocol for the electrostatic coupling between the QM and MM parts has minimal impact on the optimized structures.

The overall good agreement of the ONIOM-ME optimized structure of model M1 with the crystallographic structure, and the stability of the optimized structure relative to the electrostatic coupling between the QM and MM parts, suggests that geometries optimized at the ONIOM-ME level using model M1 can offer valuable structural information on species for which an experimental structure is not available, including those involved in reactivity. However, for better energetics we performed single point ONIOM-EE energy calculations on the ONION-ME optimized geometries.

Geometry optimizations of model M1 were also performed for the reduced spin state [Fe_4_S_4_]^+^ with different spin couplings, see the ESI† for details. Focusing on the most stable BS state, see Table S3,† we observed further elongation of the Fe–S bond lengths with respect to the same structure optimized for the oxidized [Fe_4_S_4_]^2+^ state, with an average Fe–S value of 2.36 Å against 2.35 Å for the oxidized state, which in the end is a minimal change considering the addition of one electron into the model. Natural population analysis (NPA) indicates that the added electron is essentially located on the [Fe_4_S_4_] cluster (see ESI†).

### HMBPP dehydroxylation

In the crystallographic structure of the IspH/HMBPP complex, the 4-OH group of the substrate is coordinated to the apical Fe1 atom. The first step proposed in the bio-organometallic mechanism consists of a conformational rearrangement of the substrate that, through rotation around the C3–C4 bond, replaces the Fe1–OH interaction by π-coordination with the C2=C3 double bond. Support for this scenario was provided recently by HYSCORE experiments resulting in a very weak ^17^O hyperfine coupling constant of 1 MHz. This indicated a weak to nonexistent Fe–4OH interaction, since Fe–O bonding in other Fe–S cluster-containing enzymes usually exhibits ^17^O hyperfine coupling constants in the 8–15 MHz range.^14^

To shed light on the feasibility of this rearrangement of the substrate, we performed ONIOM calculations using the most stable BS state of model M1. The reduced [Fe_4_S_4_]^+^ state of the cluster was considered, since this is the experimentally supported active species.^14^ Initially, we considered Glu126 to be deprotonated. A relaxed scan of the Fe1–O distance was carried out to simulate rotation of the 4-OH group away from the apical Fe1 atom and promote coordination of the C2=C3 double bond, see Fig. S3.† The structure highest in energy during the scan was used to locate the transition state for this rearrangement of the substrate, **TS1**, and its first order saddle point character at the ONIOM-ME level was verified through frequency calculations. According to these calculations, the 4-OH group undergoes this rotation with a barrier of 9.7 kcal mol^–1^, a result basically confirmed by the single point ONIOM-EE calculations, which indicate a barrier of 13.0 kcal mol^–1^. Relaxation of the transition state **TS1** towards the product side leads to the expected η^2^-complex **I1**, only 0.7 kcal mol^–1^ below **TS1** at the ONIOM-ME level, while at the ONIOM-EE level **I1** is predicted to be 4.7 kcal mol^–1^ above **TS1**. The structure of intermediate **I1** shows that the C2=C3 bond is well-coordinated to the apical Fe1 atom, with the Fe1–C2 and Fe1–C3 distances around 2.27 Å, while the 4-OH group is 3.58 Å away from the Fe1 atom, see Fig. 2.

In short, these calculations converge to suggest that rotation of the 4-OH group is facile, with a barrier around 10–15 kcal mol^–1^, and that the product of this rotation, the η^2^-complex **I1**, is clearly less stable than the starting 4-OH coordinated complex. At the same time, they highlight that the absolute numbers have to be taken *cum grano salis*, since the ONIOM-EE method predicts the ONIOM-ME geometry of intermediate **I1** to be less stable than that of the preceding transition state **TS1**. The impact of the chosen computational approach on the energetics of the reaction is discussed in a dedicated section, see below. Next we modeled the dehydroxylation of HMBPP by elongating the C4–O4 bond, still in the presence of the unprotonated Glu126. The located transition state **TS2** and the following intermediate **I2** are about 75 and 50 kcal mol^–1^ above the initial reactant **R** using both the ONIOM-ME and ONIOM-EE methods, see Fig. 2, which rules out this pathway.

Considering the experimentally proved crucial role of Glu126 in promoting catalysis,^11,13^ we modeled the same steps in the presence of a protonated Glu126, see Fig. 2 and S4.† First, we modified the hydrogen bonding network around Glu126 to accommodate the added proton. Several geometry optimizations were performed to determine the most stable and appropriate orientation for enabling the reaction to occur. In the most stable conformation the proton of Glu126 is oriented towards water WT1, while one of the protons of water WT1 is oriented towards the phosphate group. Incidentally, this network of hydrogen bonds also results in a suitable orientation for a H-transfer from the phosphate to the substrate.

As a first step we recalculated the energy required for rearranging the substrate from the 4-OH coordinated species **R**, to the C2=C3 coordinated intermediate **I1**, through transition state **TS1**. In the presence of a protonated Glu126, rotation of the 4-OH group is also an easy process, with a barrier around 10 kcal mol^–1^ at both the ONIOM-ME and ONIOM-EE levels. This is reasonable, as this process does not perturb the hydrogen bonding network of the active site considerably. Next, we located transition state **TS2**, corresponding to cleavage of the C4–OH bond, and we found a much lower barrier, 7.7 and 28.7 kcal mol^–1^ at the ONIOM-ME and ONIOM-EE levels, relative to the barrier of about 75 kcal mol^–1^ in the presence of a deprotonated Glu126. Furthermore, the protonation of Glu126 also results in a much more stable intermediate **I2**, see Fig. 2. In this case, the ONIOM-ME and ONIOM-EE results are in clear discrepancy, which again indicates that the energies have to be taken with caution.

The much better energetics of the C4–OH cleavage in the presence of a protonated Glu126 is due to a concerted transfer of the acidic proton of Glu126 to water WT1, while one of the protons of water WT1 is transferred to the leaving 4-OH group (see Fig. S2†), which then leaves the substrate as a water molecule rather than a hydroxyl, as in the presence of the deprotonated Glu126. In other words, it is the acidity of Glu126 that triggers the C4–OH bond cleavage through the mediating role of the WT1 water molecule. In line with this scenario, this bridged water molecule is well conserved in the available crystallographic structures of IspH (PDB codes: ; 3KE8, ; 3KE9, ; 3KEL,^33^; 3F7T,^4^; 3SZO, ; 3SZL,^5^; 3URK, ; 3UTC, ; 3UV3, ; 3UWM, ; 3MUX, ; 3UV6, ; 3UV7,^34^ and ; 4H4D^35^).

In addition, we also checked if the proton transfer assisting the C4–OH cleavage could occur from a properly oriented OH group on the phosphate, rather than from Glu126. To this end, we first transferred the proton from the phosphate to the 4-OH group and relaxed the (Glu126 protonated) structure, while constraining the distance between the 4-O atom and the transferred proton to 0.98 Å, to induce formation of intermediate **I2**. In the constrained optimized structure we observed an elongation of the C4–O bond, to 1.46 Å, still the bond is not broken. Relaxing the structure with the constraint removed resulted in the proton being transferred back to the phosphate group and the C4–O bond reforming again. This suggests that the phosphate cannot act as a promoter of the C4–O bond cleavage. As another possibility, we also checked if the terminal phosphate group could have two O atoms protonated (in this case Glu126 is deprotonated), see Fig. S10.† Starting from this di-protonated phosphate we again located **TS2**, corresponding to the dehydroxylation step. The calculated reaction barrier from the starting complex **R** is still high, around 70 kcal mol^–1^, and intermediate **I2** is of high energy, at around 39.0 kcal mol^–1^ above **R**, see Fig. S9,† ruling out also this possibility.

Finally, despite experimental evidence suggesting that the active spin state is the reduced [Fe_4_S_4_]^+^ state, we also investigated the reaction profile using the oxidized state [Fe_4_S_4_]^2+^. However, calculations indicate that intermediates **I1** and **I2** are high in energy, 27.4 and 34.5 kcal mol^–1^ above **R** at the ONIOM-EE level, which makes this possibility less likely to happen. These results suggest that the rotation and dehydroxylation of the 4-OH group are much easier in the reduced state than the oxidized one. This suggests that the electron transfer (ET) to the cluster, reducing the [Fe_4_S_4_]^2+^ state to the [Fe_4_S_4_]^+^ state, should occur before rotation of the 4-OH group.

In conclusion our analysis indicates that dehydroxylation can occur only on the reduced [Fe_4_S_4_]^+^ state in the presence of a protonated Glu126. For this reason, in the following we focus on a more detailed analysis of the **I1** and **I2** intermediates along this reaction pathway.

To have a better understanding of the energy difference between 4-OH coordination and C2=C3 coordination, we also investigated the simple model shown in Fig. S1.† This model allowed us to focus on the core interaction between the cluster and the substrate, without the complication of surrounding groups. Calculations were performed in the gas-phase, and for both the [Fe_4_S_4_]^2+^ and [Fe_4_S_4_]^+^ states (see the details reported in the ESI†). The optimized geometries are reported in Table S14.† According to the calculations, the C2=C3 coordinated geometry is favored by –0.1 and –10.7 kcal mol^–1^ in the [Fe_4_S_4_]^2+^ and [Fe_4_S_4_]^+^ states, respectively. This is in line with calculations using the M2 model discussed previously, where we found that the formation of intermediate **I1** competes more with 4-OH coordination in the reduced state, than in the oxidized state.

An analysis of the NPA charges and spin densities of the QM region along the reaction pathway in the case of the catalytically active species corresponding to the protonated Glu126 and reduced [Fe_4_S_4_]^+^ state indicates that there are significant changes to the cluster total charge and spin density between the 4-OH and C2=C3 coordinated species **R** and **I1**, rather than between **I1** and **I2**, as indicated by the NPA charges of –2.03*e*, –1.54*e* and –1.24*e* in **R**, **I1** and **I2**, and spin densities of 0.95*e*, 1.24*e* and 1.39*e*. Indeed, these results indicate that most of the changes in the electronic structure of the cluster are due to the rotation of the 4-OH group, which acts as a donor to increase the electron density on the cluster, while the C=C double bond in **I1** and the allyl moiety in **I2** have π* orbitals available to accept back-donation from the cluster. Consequently, the 4-OH rotation step results in a clear transfer of about 0.5*e* to the cluster from the substrate in **I1**, and an additional transfer of about 0.3*e* in the dehydroxylation step, **I1** to **I2**, which is consistent with the formation of a more acidic π* orbital in the allyl moiety of **I2**. Finally, the overall NPA charge of the allyl group in **I2** amounts to 0.43*e*, which indicates that the allyl group has substantial neutral character.

**Fig. 3:**
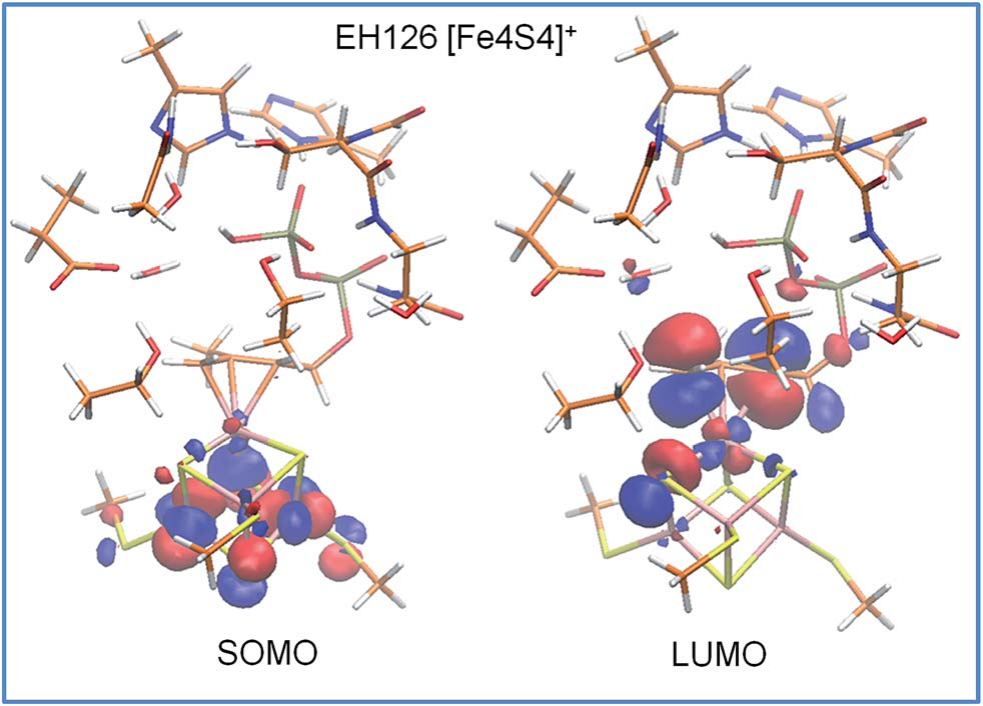
Frontier molecular orbitals of intermediate **I2** for the reduced [Fe_4_S_4_]^+^ state in the presence of a protonated Glu126. The molecular orbital isosurfaces are plotted at 0.03 a.u.

An analysis of the molecular orbitals of **I2**, in the enzymatically active reduced state with a protonated Glu126, shows more communication between the substrate and the cluster. For instance, the SOMO is concentrated on the iron cluster while the LUMO is localized on the allyl moiety, which can act as an electron acceptor in the following electron transfer and protonation steps, see Fig. 3. A complete list of the frontier orbitals of **I2** for the protonated and deprotonated Glu126, and the reduced and oxidized cluster states is reported in the ESI.†

**Fig. 4:**
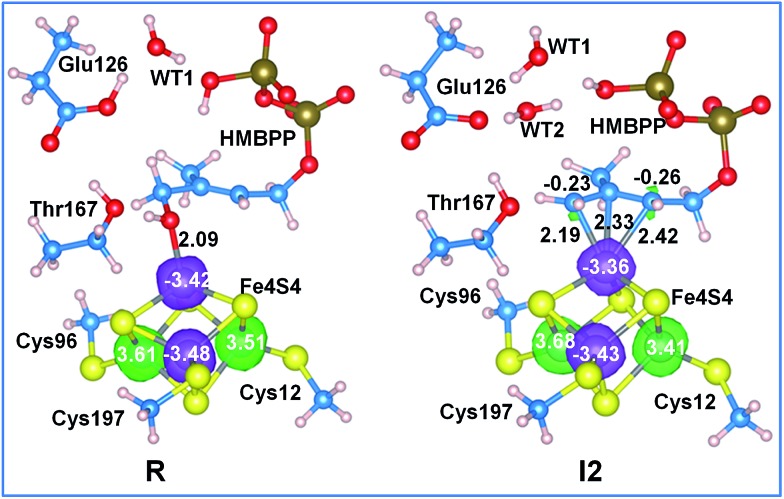
Spin density on the Fe atoms of structures **R** and **I2** along the reaction pathway with the [Fe_4_S_4_]^+^ cluster in a reduced state. The spin densities are represented by transparent spheres (α: green and β: purple) and are plotted at 0.03 a.u. Key bond distances are reported in Å.

Further analysis of the NPA charges and absolute spin densities does not show a remarkable difference between the four Fe atoms, with one of them having a clearly different oxidation state, see Fig. 4 and Table S6.† In fact, the NPA charges on the Fe atoms in **R**, **I1** and **I2** range between 1.02 and 1.23*e*, while the absolute spin densities range between 3.35 and 3.68*e*. This prevents the assignment of a formal oxidation state (iii) to one of the Fe atoms in the 3Fe(ii)/1Fe(iii) [Fe_4_S_4_]^+^ reduced state cluster. Finally, we also found a remarkable amount of excess α spin density, with an average value of 0.23*e* per S atom, on the bridged sulfide anions. Consistently, with similar conclusions reported for [FeFe]-hydrogenases^36,37^ and for model complexes,^38–40^ this shows the important role of the sulfur ligands in tuning the redox properties of the iron–sulfur cluster, with the sulfur atoms acting like reservoirs of electron density to compensate for changes in the electronic state of the cluster.

NBO analysis does not show any orbital overlap between the apical iron atom and the coordinated carbon moieties in **I1** and **I2**, despite the short Fe–C distances. We thus extended the analysis of the nature of the Fe–C interaction by calculating the Natural Bond Critical Points (NBCPs).^41^ Based on the atom in molecules (AIM) concept, the presence of a CP on the line joining two atoms indicates a chemical bond, while the electron density at the CP measures the strength of the bond. Finally, the sign of the Laplacian of the electron density, ∇^2^ *ρ*(*r*), is an indication of the concentration or depletion of electron density, with ∇^2^ *ρ*(*r*) < 0 and >0 indicating the covalent or ionic character of the bond.^42–44^

According to this analysis, the Fe–O and Fe–C interactions in **R**, **I1** and **I2** have dominant ionic character, since the density at the CP along these bonds is never greater than 0.07 a.u., and the ∇^2^ *ρ*(*r*) value is constantly positive. For comparison, for a strongly covalent bond, such as the C4–O bond, the electron density at the CP is 0.2442 a.u., and the ∇^2^ *ρ*(*r*) value is negative (see Table S12†). Interestingly, the same analysis performed on the Fe1–S bonds indicates that these bonds also have ionic character, as suggested by the very low electronic densities and positive Laplacians at the CPs located along these bonds.^45^ This is consistent with a similar analysis on methane monooxygenases,^46^ and with the dominant ionic interactions reported for several transition metals complexes.^45,47–49^

### QM/MM convergence and DFT+U calculations

To test the convergence of our results with respect to the size of the QM part in the ONIOM calculations, we compared the energies discussed so far, achieved using model M1, with 92 atoms in the QM part, with the ONIOM-ME energies achieved using model M2, with 160 atoms in the QM part. These tests indicate that the overall scenario provided by models M1 and M2 is very similar, and using the larger model M2 has no impact on the overall conclusions discussed above, although it is clear that the specific energy values are somewhat dependent on the specific model used. In fact, both models predict a rather small barrier for rotation of the 4-OH group, that dissociation of the C4–OH bond is rate determining, and that the resulting allyl coordinated intermediate **I2** is higher in energy than the starting 4-OH coordinated species **R**, see Fig. S7.† This is consistent with the results of Iwasaki *et al.*, which indicated that the hydrogen bonds established by the backbone peptide tune the electronic structure and the geometry of the Fe_2_S_2_ cluster in Rieske type proteins,^50^ and in model iron–sulfur complexes.^51^ Despite this positive test, we wondered if the extension of the hydrogen bond pattern and the resulting interactions around the iron cluster would affect our conclusions, since in the ONIOM-based calculations we considered only the first protein/water shell around the substrate, and we cut the cluster-bonded cysteines at the Cα–Cβ bond. To shed light on this issue we investigated the larger model M3, with 456 atoms, using DFT+U and plane wave calculations, as implemented in VASP, which performs quite well for magnetic molecules.^52^ Indeed, earlier studies showed that VASP calculations reproduced G09 results within a 0.5 kcal mol^–1^ limit of accuracy.^53^ Using model M3 we re-optimized the structures along the favored reaction pathway corresponding to the reduced [Fe_4_S_4_]^+^ state in the presence of a protonated Glu126. To mimic the overall protein structure, a geometry optimization of the intermediates was performed by constraining the position of the Cα atoms at the boundaries of the cluster. For the transition states **TS1** and **TS2**, we further constrained the bond breaking Fe–O and C–O distances at the values resulting from the ONIOM G09 calculations discussed above. Comparison between the ONIOM M1 and DFT+U M3 structures results in RMSDs between the heavy atoms smaller than 0.04 Å, see Table S13† for the optimized parameters, which validates all the structural considerations based on the M1 geometries. Further, comparison of the M3 and M2 energy profiles, see Fig. S13,† shows a very good agreement between the two methods, since the largest deviations are small differences between the relative stability of **TS1** and **I1**, which are predicted to be slightly more stable using the DFT+U profile. In short, these tests confirm that our results can be considered converged both in terms of geometry and energy.

### Allyl protonation

**Fig. 5:**
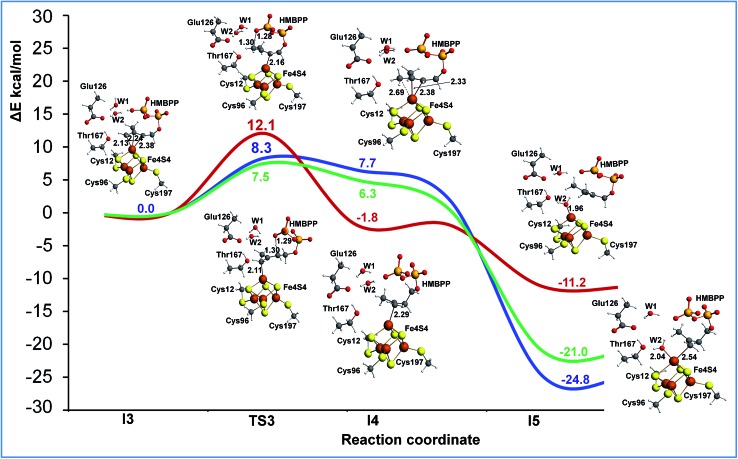
The ONIOM (B3LYP/TZVP:Amber)-EE reaction profile of the allyl protonation step. Key bond distances are reported in Å. The red line corresponds to protonation at C4, the blue line to protonation at C2, and the green line to protonation at C2 using the DFT+U approach and M3 model.

The last step of the IspH mechanism is the protonation of two different carbon atoms in the HMBPP skeleton of intermediate **I2**, namely C2 to produce IPP and C4 to produce DAMPP. To investigate this step we performed two different relaxed scans to simulate the proton transfer from the phosphate group of HMBPP to C2 and C4 (see Fig. S14†). Focusing on the potential energy surface calculated using ONIOM-EE and model M2, protonation at C2 (blue and green) is easier than protonation at C4 (red) (see Fig. 5). The reaction barrier for the proton transfer from the phosphate group to C2 is clearly lower (8.3 kcal mol^–1^) than the barrier to protonate C4 (12.1 kcal mol^–1^). This is in good qualitative agreement with the experimental evidence that suggests IspH produces IPP and DAMPP in a 5 : 1 ratio.^6,12^

Collapsing the two transition states towards the product side, with the substrate still coordinated to the apical Fe1 atom, resulted in kinetic products of limited stability. A much more stable structure, **I5**, was achieved by coordination of the formed water molecule to the apical Fe1 atom. This concerted rearrangement of the products, with a relatively large dislocation of several molecules, is practically impossible to model with static methods, so we did not perform attempts in this direction.

Nevertheless, we remark that protonation at either C2 or C4 from the phosphate tail of the substrate locates an additional formal negative charge on the phosphate. Considering the phosphate group is located near the protein surface, it is tempting to suggest that solvation could drive the final product outside the HMBPP binding site.

Also, in this case we recalculated the reaction profile using the larger model M3 and the DFT+U approach to ensure the convergence of the ONIOM results. As in the case of the initial part of the reaction profile, Fig. S13,† the DFT+U calculations also reproduce with high accuracy the ONIOM-EE numbers for proton transfer from the phosphate to the allyl moiety of the substrate, which again confirms the convergence of the results with respect to the model size (see Fig. 5).

A paramagnetic complex is formed upon formal e^–^/H^+^ transfer in which the iron–sulfur cluster has an oxidation state of [Fe_4_S_4_]^3+^, which is similar to that involved in the catalysis of the high-potential iron–sulfur protein (HiPIP) family. The LUMOs (LUMO–LUMO+5) of the final products are located on the cluster and are supposed to be populated under further reduction processes to activate the cluster (see Table S10†).

## Conclusion

We report the first computational investigation of the elementary steps of IspH catalysis using a state-of-the-art ONIOM technique coupled with a broken symmetry DFT approach. Furthermore, we have adopted plane wave DFT+U calculations for rescoring some key stationary points already optimized within the ONIOM approach. The main result is that our calculations fully support the bio-organometallic mechanism, while ruling out a Birch-like mechanism, with the formation of radical species during the dehydroxylation step.

Focusing on the favored bio-organometallic mechanism, our calculations indicate that the active species involved in IspH catalysis is the reduced cluster, [Fe_4_S_4_]^+^. The reduction of the iron–sulfur cluster helps the rotation of the 4-OH group of the substrate away from the iron cluster, with the π-coordination of the C2=C3 double bond. The rotated OH group is engaged in a H-bond interaction with Glu126. Proton transfer from Glu126, mediated by a conserved water molecule, triggers dehydroxylation of HMBPP and its conversion into a π-coordinated allyl moiety involving the C2–C4 atoms. Calculations suggest that these steps can only occur with the iron cluster in a reduced state, due to the high energy of the same intermediates in the presence of an iron cluster in an oxidized state. The key role of Glu126 is further demonstrated by the high energy of the dehydroxylation step in the case of a deprotonated Glu126. As for protonation of the formed allyl intermediate, our calculations suggest a crucial rearrangement of the active site in order to release the final product. Consistent with experiments, the protonation of the C2 and C4 atoms of HMBPP is competitive. Finally, we validated the ONIOM calculations by evaluating the relative energy of the key intermediates along the favored pathway using DFT+U plane wave calculations on a large quantum mechanics model including up to 456 atoms.

As a concluding remark, we note that the reaction profile calculated by the ONIOM and DFT+U approaches predicts that the dehydroxylation of HMBPP is the rate determining step, in agreement with recent inhibition studies showing that (*E*)-4-mercapto-3-methylbut-2-en-1-yl diphosphate and (*E*)-4-amino-3-methylbut-2-enyl 1-diphosphate are potent inhibitors of IspH in the nano-molar range.^54,55^ Both molecules are HMBPP analogues, where the 4-OH group is replaced by thiol and amino groups respectively.
