# Supplementary material for: Mechanistic insights into the reductive dehydroxylation pathway for the biosynthesis of isoprenoids promoted by the IspH enzyme
Source: Chem Sci. 2015 Jun 22;6(10):5643–51. doi: 10.1039/c5sc01693b (PMC5511988; doi:10.1039/c5sc01693b)
Supplement: Supplementary file 1 [file SC-006-C5SC01693B-s001.pdf]

Electronic Supplementary Material (ESI) for Chemical Science.
This journal is © The Royal Society of Chemistry 2015

Supporting Information for:

Mechanistic Insights on the Reductive Dehydroxylation Pathway for the Biosynthesis of Isoprenoids Promoted by the IspH Enzyme.

Safwat Abdel-Azeim, Abdesslem Jedidi, Jorg Eppinger and Luigi Cavallo

Table of contents

1. Computational Details
2. Figure S3: Relaxed scan of Fe-O and C-O to simulate the dissociation of the two bonds for the de-protonated Glu126 using model M1 and ONIOM-MM(B3LYP/TZVP:Amber).
3. Figure S4: Relaxed scan of Fe-O and C-O to simulate the dissociation of the two bonds for the protonated Glu126 using model M1 and ONIOM-MM(B3LYP/TZVP:Amber).
4. Figure S5: Relaxed scan of C-O to simulate the dissociation of the 4-OH group using protonated phosphate group and model M1.
5. Figure S6: ONIOM(B3LYP:TZVP/Amber)-EE reaction profile of the rotation and dehydroxylation step using different QM models calculated for the deprotonated Glu126.
6. Figure S7: ONIOM(B3LYP:TZVP/Amber)-EE reaction profile of the rotation and dehydroxylation step using different QM models calculated for the protonated Glu126.
7. Figure S8: Reaction profile of the 4-OH rotation and elimination calculated using ONIOM(B3LYP/TZVP:Amber)-ME level of theory and model M1 and M2 for the protonated and deprotonated Glu126.
8. Figure S9: Reaction profile of the 4-OH Rotation and elimination step calculated at ONIOM (B3LYP/TZVP:Amber)-EE using M2.
9. Figure S10: HMBPP substrate with the atom numbering reported in the following tables
10. Figure S11: Balls and sticks representation of the reaction profile stationary points for the reduced state $[Fe4S4]^+$ and deprotonated Glu126
11. Figure S12: Balls and sticks representation of the dehydroxylation reaction profile stationary points for the oxidized state $[Fe_4S_4]^{+2}$ and protonated Glu126.
12. Figure S13: Reaction profile of the 4-OH Rotation and elimination step calculated at ONIOM (B3LYP/TZVP:Amber)-EE using M2 (red) and DFT+U (blue) using M3.
13. Figure S14: Relaxed scans of the C-H bond distance to simulate the protonation at C2 (blue) and at C4 (red). Relaxed scans are calculated using model M1 and ONIOM-MM (B3LYP/TZVP:Amber) level of theory.
14. Figure S15: Balls and sticks representation of the reaction profile stationary points of the e-/H^+ transfer step calculated using the reduced state $[Fe_4S_4]^+$ and the spin densities are reported.
15. Table S1: ONIOM optimized parameters of IspH calculated at B3LYP/TZVP:AMBER level using different BS states and the oxidized $[Fe_4S_4]^{2+}$.
16. Table S2: ONIOM-EE relative energy of the BS states calculated at B3LYP/TZVP: AMBER level using the reduced $[Fe_4S_4]^+$ and the oxidized $[Fe_4S_4]^{2+}$ states.
17. Table S3: ONIOM optimized parameters of IspH calculated at B3LYP/TZVP:AMBER level using the most stable BS state and the reduced state $[Fe_4S_4]^+$.
18. Table S4: Natural Population Analysis charges and spin densities calculated for the QM region using model M2 (160 atoms) using B3LYP/TZVP calculated for the reduced state and the protonated Glu126.
19. Table S5: Natural Population Analysis charges and spin densities calculated for the QM region using model M2 (160 atoms) using B3LYP/TZVP calculated for the reduced state and the deprotonated Glu126.
20. Table S6: Natural Population Analysis charges and spin densities calculated for the QM region using model M2 (160 atoms) using B3LYP/TZVP calculated for the oxidized state and the protonated Glu126.
21. Table S7: Frontier molecular orbitals of EH126 in the reduced state.
22. Table S8: Frontier molecular orbitals of EH126 in the oxidized state.
23. Table S9: Frontier molecular orbitals of E126 in the reduced state.
24. Table S10: Frontier molecular orbitals of EH126 second step reaction step.
25. Table S11: NPA charges and spin densities of the stationary points of the e^-/H^+ coupled reaction calculated for the QM region and the protonated Glu126 using model M2 and optimized at ONIOM(B3LYP/TZVP:Amber) level of theory for the reduced state.
26. Table S12: Reported the topological analysis of the electron density of selected bonds on the local minima of the rotation and elimination of 4-OH group using NBO analysis.
27. Table S13: ONIOM optimized parameters of IspH calculated at B3LYP/TZVP: AMBER level using the reduced state $[Fe_4S_4]^+$ and in parenthesis the optimized parameters using DFT+U and plane wave model of theory.

28. Appendix: Gas phase and water solution calculations
29. Table S14: DFT/TZVP optimized parameters of the IspH active site model depicted in figure 2 for R and I1
30. Table S15: Optimized parameters of R and I using the simple active site model in figure 2 depicted in the main text.
31. Table S16: NPA charges and spin densities of the reaction profile calculated the oxidized state and the reduced states of the cluster using QM gas phase model depicted in figure 2 of the main text.
32. ONIOM-optimized coordinates of the QM region (M2) of the reaction profile stationary points for the protonated glutamate.

Computational details.

Active site calculations. The initial coordinates of IspH were taken from the crystallographic structure solved to 1.7 Å (PDB code 3KE8). [1] Initial calculations were performed on a simple small model of the active site missing the pyrophosphate group (see Figure 2). This model includes the iron-sulfur cluster, its coordinating thiolates (Cys 12, 96 and 197) and the substrate represented by 2-methylbut-2-enyl alcohol. Cysteines side chains are truncated at the Cβ, and the broken Cα-Cβ bond is capped by a H atom. In the case of the IspH $[Fe_4S_4]$ cluster, the antiferromagnetic (AF) coupled state has parallel spin on two iron atoms aligned opposite to the spin on the other two irons. In practice, BS–DFT[2, 3] states were obtained by partitioning the system into the appropriate fragments to generate a specific AF state. The four iron atoms (Fe1-4) were divided into four separate fragments and the remaining part of the system into a separate fragment. Six AF states were generated, corresponding to the following spin combinations on the Fe atoms: ααββ, αβαβ, αββα, βααβ, βαβα, and ββαα. All the calculations were carried out with the B3LYP functional[4-7] and the all-electron triple-ζ TZVP[8, 9] basis set using the G09 program. [10] Two oxidation state of the cluster were considered, corresponding to the reduced $[Fe_4S_4]^+$ and the oxidized $[Fe_4S_4]^{+2}$ states. All the broken symmetry states were generated using the fragment guess approach using Gaussview 5.[11]

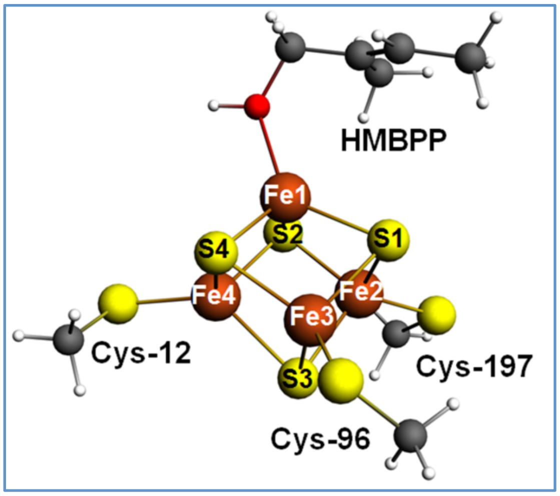

Figure S1: Representation of the $[Fe_4S_4]$ cluster with the carbon skeleton of the substrate, and the numbering used in the discussion.

Since Greco et al [12] have shown that the Mulliken charges of the $[Fe_4S_4]$ clusters are scarcely sensitive to the coupling pattern between the iron sites, we have calculated the restrained electrostatic potential (RESP) charges (necessary for the ONIOM calculations) for the cluster, the coordinated cysteines and the complete HMBPP substrate using the most stable BS wavefunction. Geometry optimizations were performed for all the generated BS states and the most stable state was selected for the reactivity study. Frequency calculations were performed in order to characterize the located stationary points as minimum energy or first order saddle point structures. All the reported energies are the electronic energy of the system.

ONIOM Calculations. Starting from the IspH coordinates (PDB code 3KE8), protons were added using the HBUILD facility implemented in CHARMM[13] with the AMBER force field.[14] Parameters for the HMBPP substrate were constructed using ACPYPE (AnteChamber PYthon Parser interfacE),[15] the General Amber Force Field (GAFF)[16, 17] and the RESP[17] charges calculated with the Antechamber program,[17] based on the ESP charges calculated at DFT/TZVP level using G09. Molecular mechanics (MM) relaxation of the whole system with fixing the substrate and the iron-cluster was performed to remove clashes between atoms. The MM optimized structure was used as the initial structure for the ONIOM calculations. All lysines and arginines were

considered positively charged, while aspartate and glutamate were considered negatively charged, except for glutamate 126 that was considered in two states, namely protonated (neutral) and deprotonated (negatively charged). All the cysteines coordinated to the iron-cluster were considered as deprotonated. In the Quantum Mechanics/Molecular Mechanics (QM/MM) method, the enzyme is divided into two parts: an inner part treated at QM level and an outer part treated at MM level. In our case the inner part or QM region is described at B3LYP/TZVP level of theory and the surrounding protein residues (MM region) is described using the empirical AMBER force field. The QM region is consisting of the iron-sulfur cluster, the substrate HMBPP, the three cysteines coordinating the cluster (Cys 12, 96 and 197), Thr167, Glu126 and 8 crystal waters inside the active site found important for the geometrical optimization from initial trials. The valence of the cutting bonds at the interface between the QM and the MM regions is completed by adding hydrogen link atom. The cutting was done between the Cα-Cβ bonds. Geometry optimization of the enzyme was carried out at the ONIOM (B3LYP/TZVP: AMBER) with a mechanical embedding of the MM charges (ME) level of calculations without any constrains using model 1 (92 atoms) (see figure 3). ONIOM-electronic embedding (EE) single-point energy calculations were then performed to refine the energetics at the same level and at more extended QM model 2 (160 atoms) (see Figure 3) For these calculations we used the M1 optimized geometry and we partitioned differently the system into QM and MM parts, to have a larger QM part. The approach used to generate the broken symmetry spin states in the gas phase was kept. Thus, different BS states were generated and the most stable spin state was used to model reactivity (see Table S1-3). We have investigated the reaction mechanism of IspH with two different state of Glu126 (protonated and deprotonated). Frequency calculations were performed in order to verify the located stationary points to be minimum (free of imaginary frequencies) or maximum (only one imaginary frequency corresponding to the reaction coordinates) only for the QM region. Natural Population Analysis (NPA) was carried out using Natural Bond Orbital 6 (NBO) program.[18]

DFT+U plan wave calculations.

Finally, we have rescored the stationary points localized with the ONIOM model with a DFT+U method using the larger QM model M3 (456 atoms). This larger model allowed to check for the effect of the partitioning of the system into QM and MM regions, and to have a better control of the effect of the surrounding residues on the $[Fe_4S_4]$ cluster along the reaction profile. In the plane wave calculations we performed spin-polarized periodic calculations based on DFT in the generalized gradient approximation (using the PW91 exchange-correlation functional) as implemented in the VASP [19-22] code. For the DFT+U calculations, the Hubbard potential was applied to the d orbital of Fe atoms and taken as U_{eff}=U-J=3.2 – 1.0 = 2.2 eV. [23] A plane-wave basis set (with a kinetic energy cutoff of 400 eV) describes the valence electrons: 1 electron for H, four electrons for C, five electrons for N and P, six electrons for O and S, and eight electrons for Fe, to include the 4s and 3d electrons. The core electrons were replaced by projector augmented wave (PAW) pseudo-potentials.[24, 25] Geometry optimization of key intermediates was performed by constraining the position of the Cα atoms at the boundaries of the cluster. The relaxation of the atomic positions in the supercell took place until energy differences are smaller than 0.001 eV. The clusters are placed in a repeating cubic box with an edge length of 32 Å, large enough to prevent interaction between the cluster with its image.

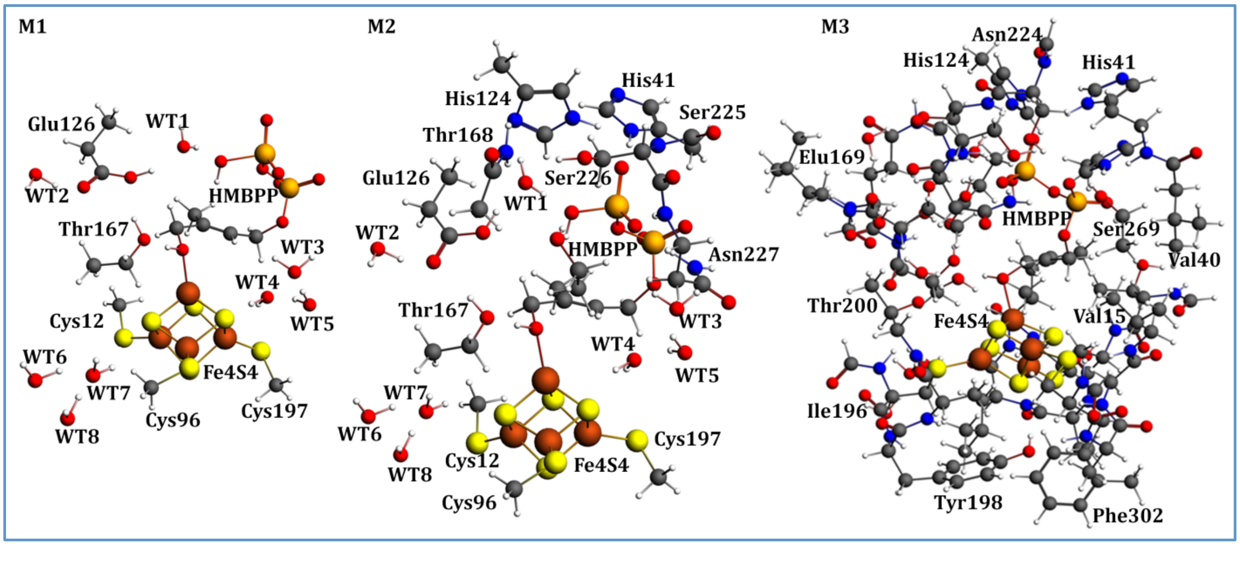

Figure S2: Representation of the different QM models (M1-M3) used in this work. Model M1 (92 atoms) is used for the ONIOM-ME geometry optimizations. Model M2 (160 atoms) is used in the ONIOM-EE calculations to refine the energy of the M1 optimized geometries. Model M3 is used for the DFT+U calculations (456 atoms).

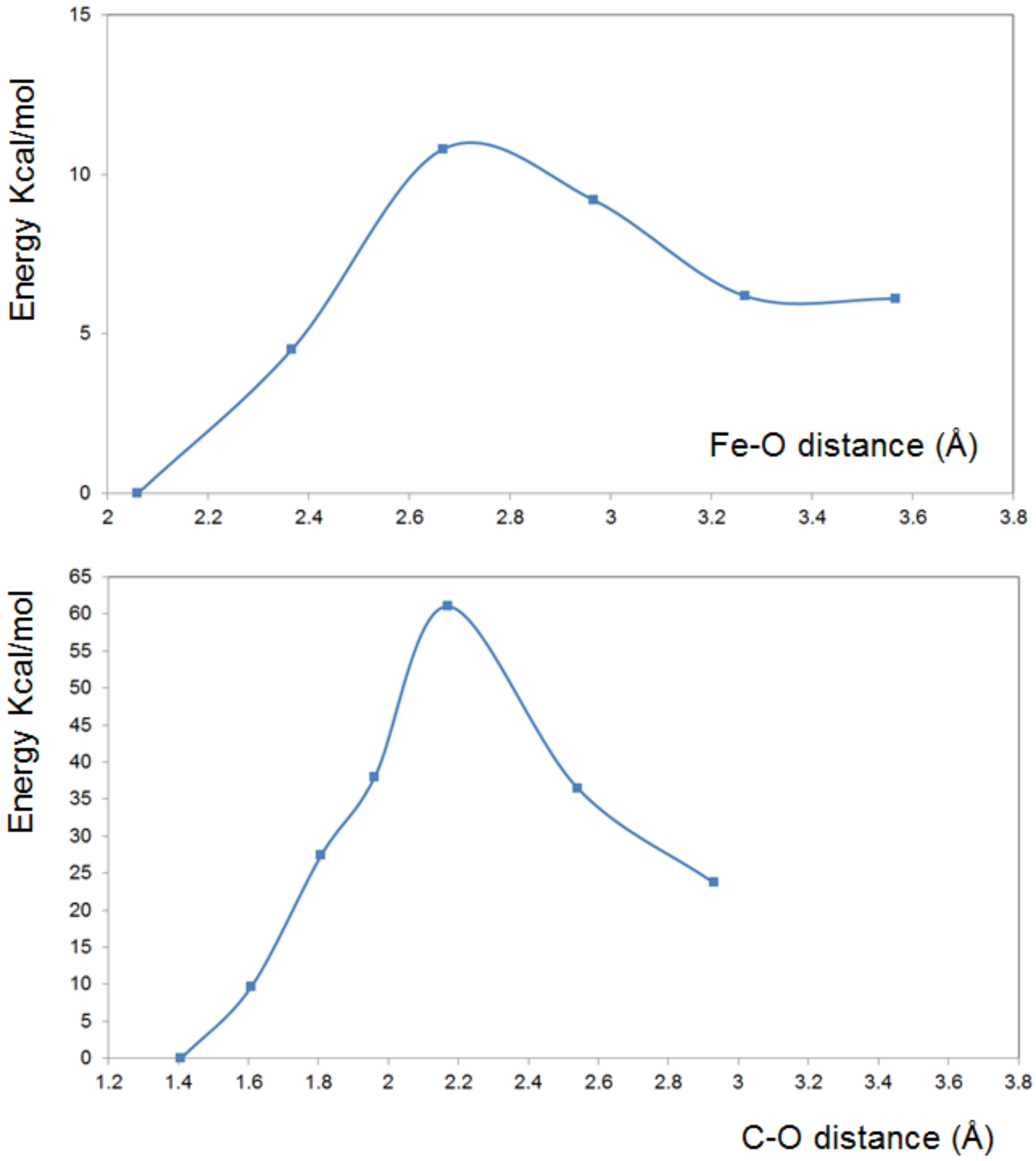

Figure S3. Relaxed scan of Fe-O and C-O to simulate the dissociation of the two bonds for the de-protonated Glu126 using model M1 and ONIOM-MM(B3LYP/TZVP:Amber).

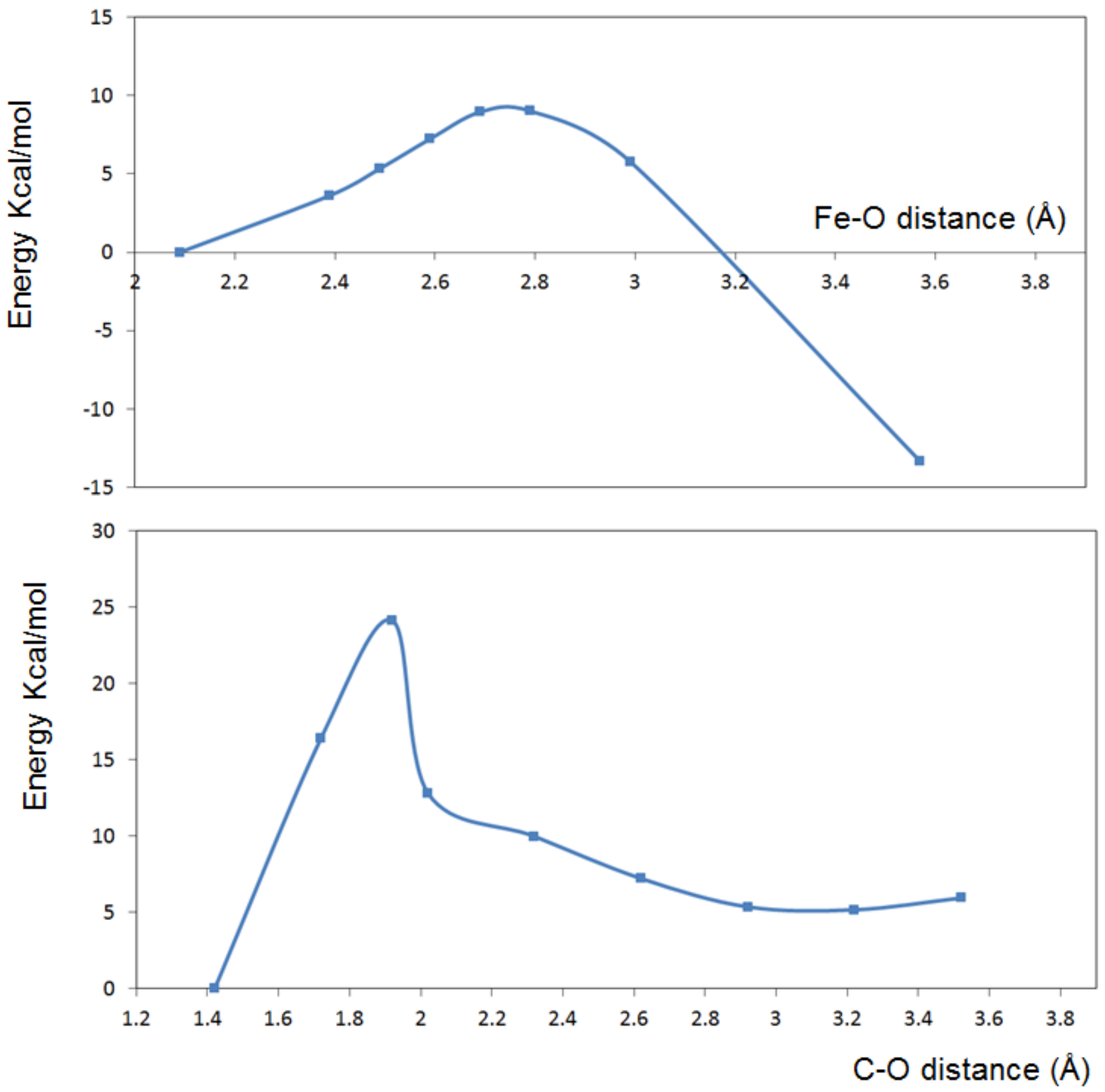

Figure S4. Relaxed scan of Fe-O and C-O to simulate the dissociation of the two bonds for the protonated Glu126 using model M1 and ONIOM-MM(B3LYP/TZVP:Amber).

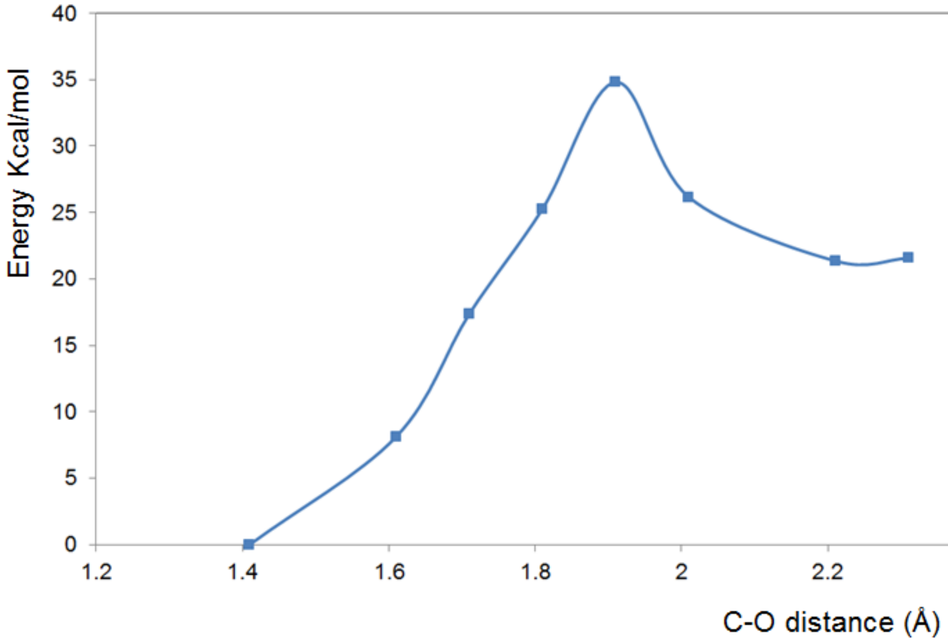

Figure S5. Relaxed scan of C-O to simulate the dissociation of the 4-OH group using protonated phosphate group and model M1

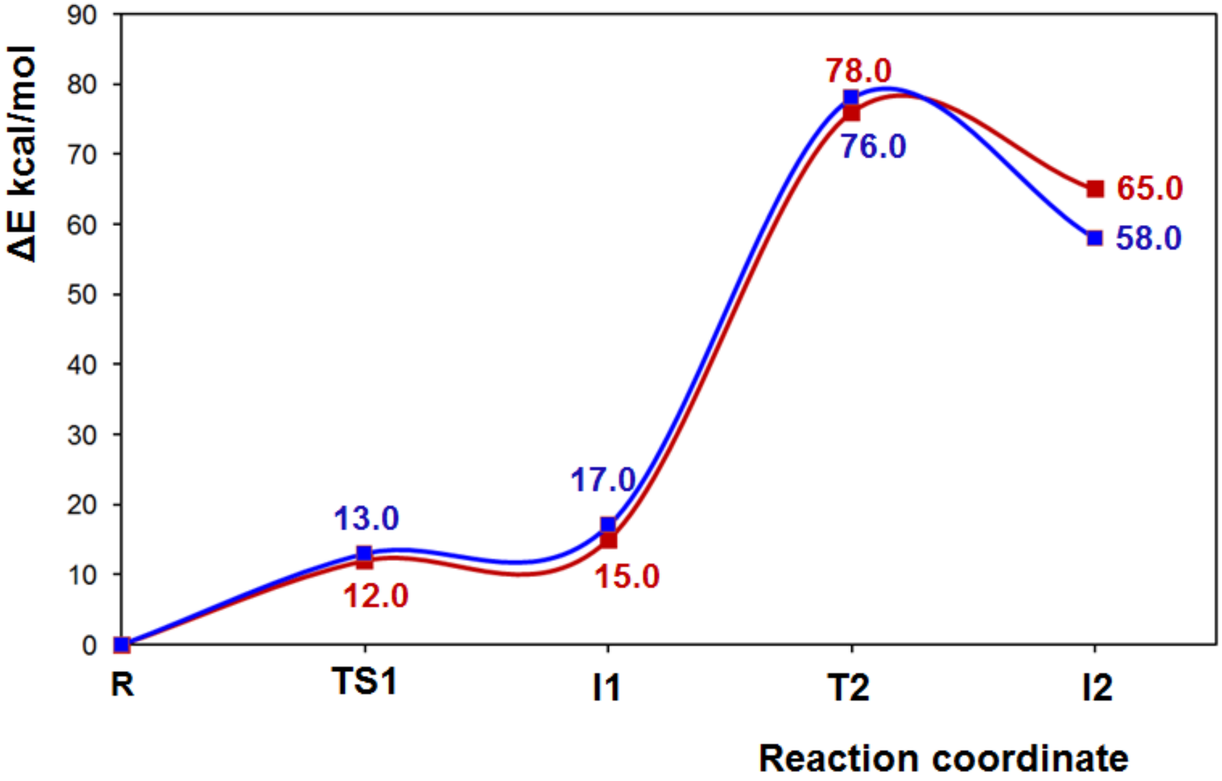

Figure S6. ONIOM(B3LYP:TZVP/Amber)-EE reaction profile of the rotation and dehydroxylation step using different QM models (M1:blue and M2:red) calculated for the deprotonated Glu126.

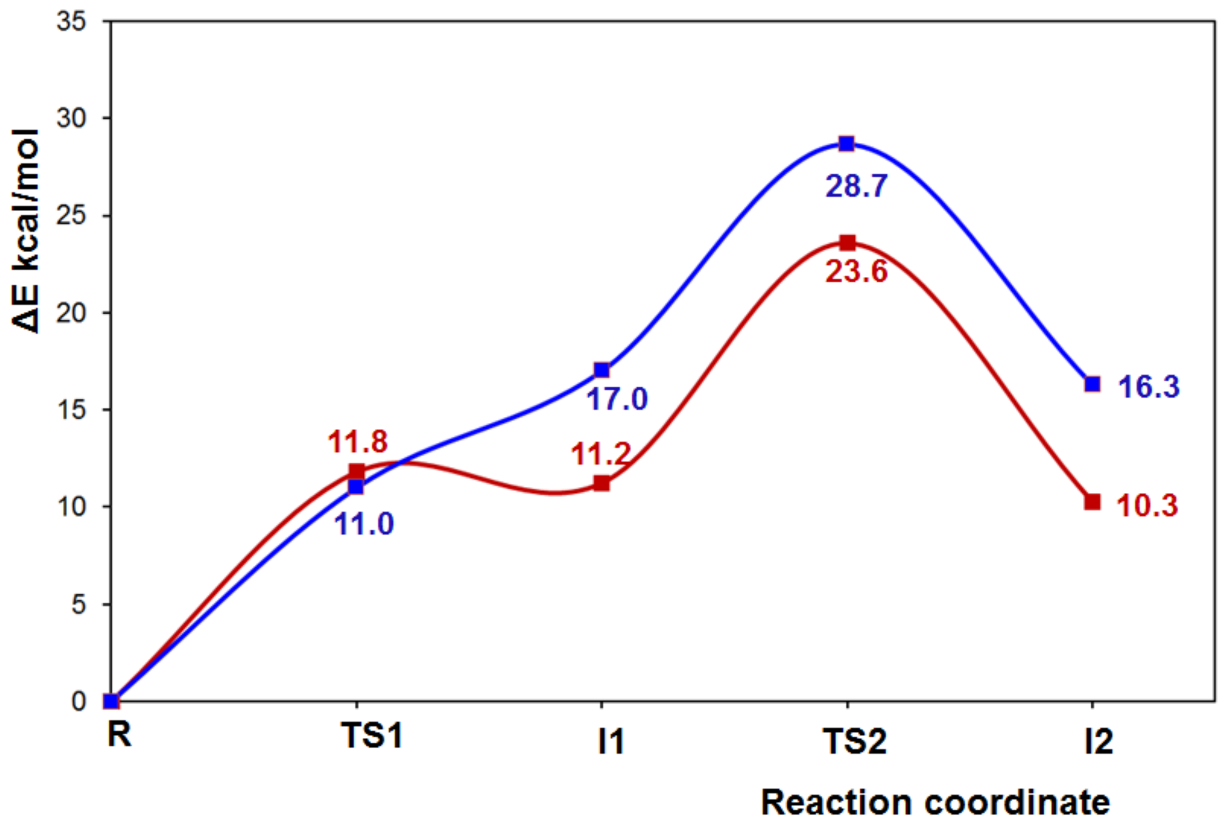

Figure S7. ONIOM(B3LYP:TZVP/Amber)-EE reaction profile of the rotation and dehydroxylation step using different QM models (M1:red and M2:blue) calculated for the protonated Glu126

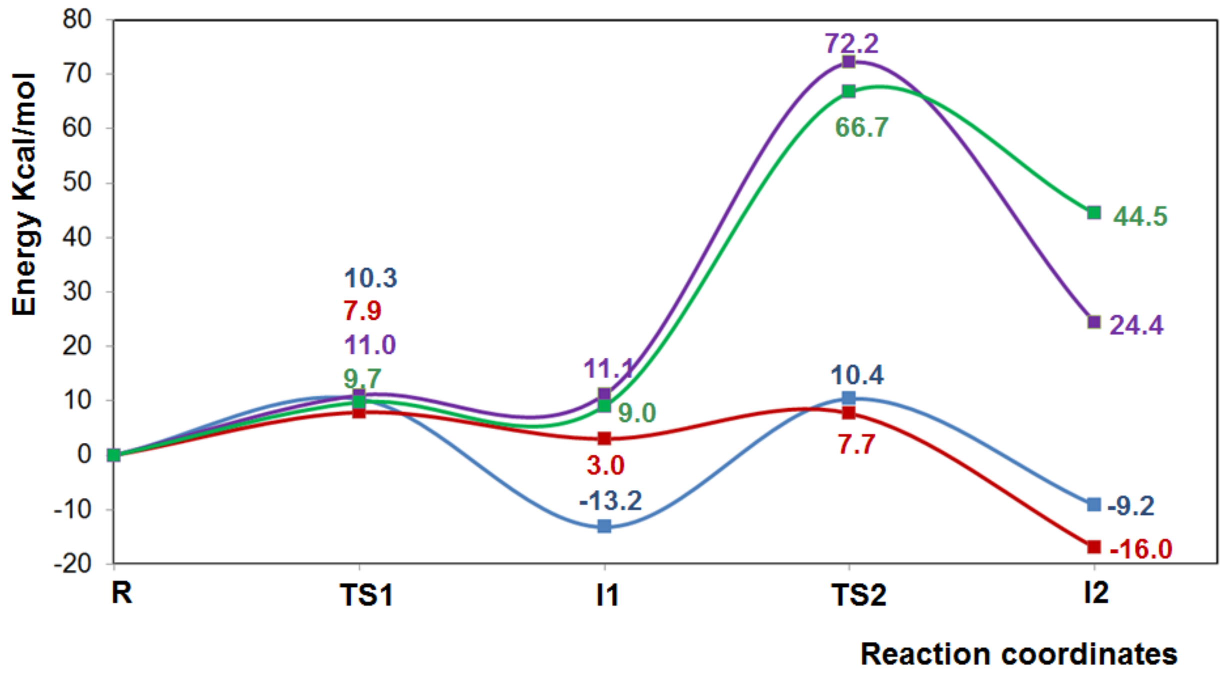

Figure S8. Reaction profile of the 4-OH rotation and elimination calculated using ONIOM (B3LYP/TZVP:Amber) ME level of theory and model M1 and M2 for the protonated and deprotonated Glu126. Color code; light blue: protonated (M1), red: protonated E126 (M2), purple: deprotonated E126 (M1) and green: deprotonated E126 (M2).

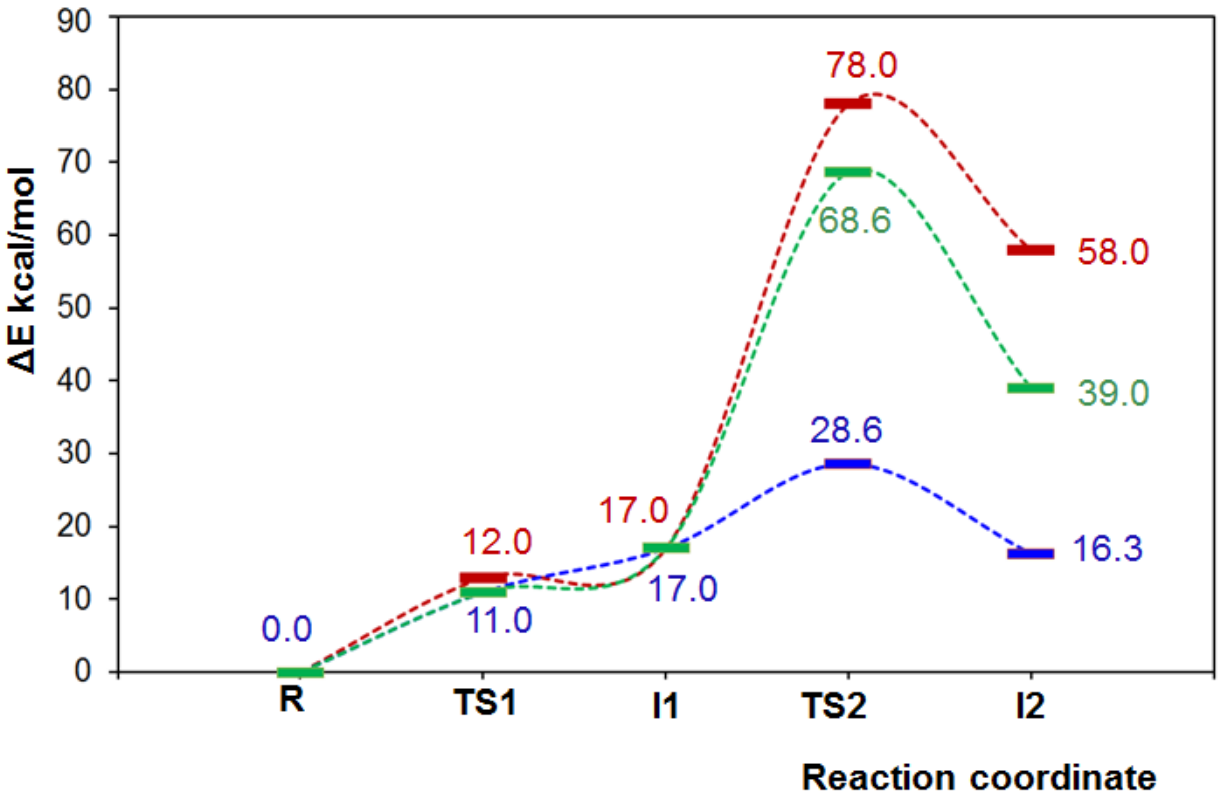

Figure S9. Reaction profile of the 4-OH Rotation and elimination step calculated at ONIOM (B3LYP/TZVP:Amber)-EE using M2. Color code: red is for the deprotonated Glu126, green for the di-protonated pyrophosphate and blue is for the protonated Glu126.

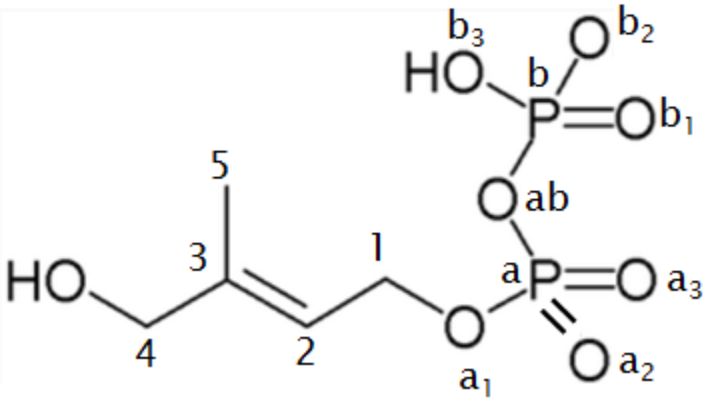

Figure S10. HMBPP substrate with the atom numbering reported in the following tables

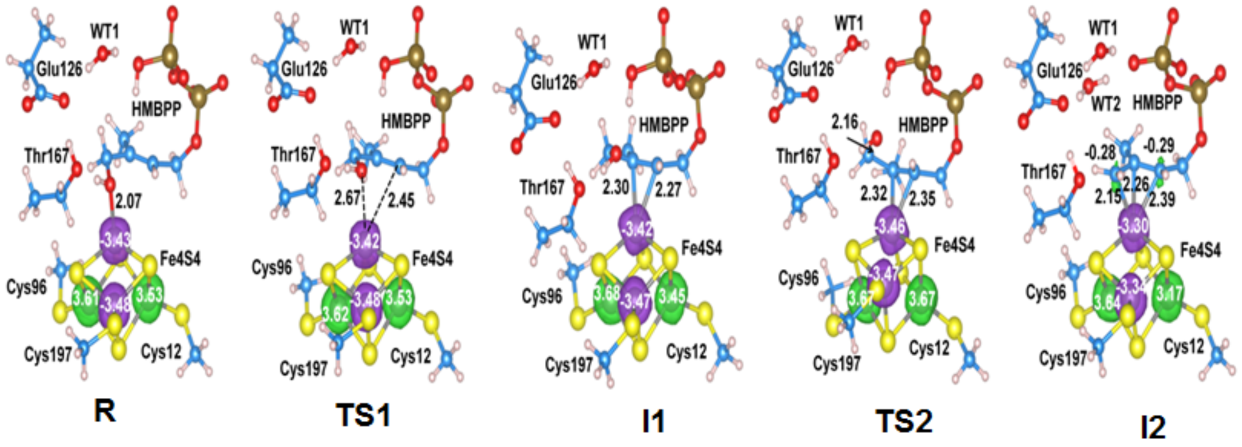

Figure S11. Balls and sticks representation of the reaction profile stationary points for the reduced state [Fe4S4]+ and deprotonated Glu126. Spin densities are represented by transparent spheres (α: green and β: purple), the isosurfaces are at 0.03 a.u. Key bond distances also are reported in Å.

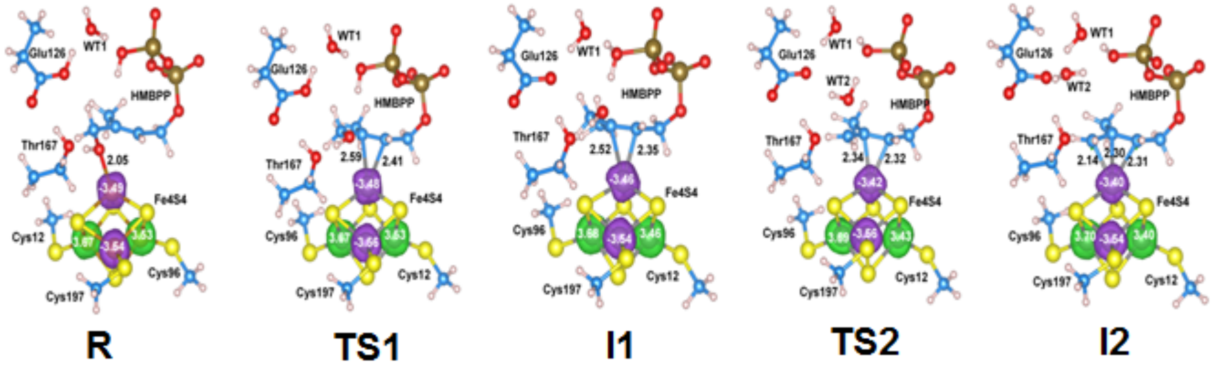

Figure S12. Balls and sticks representation of the dehydroxylation reaction profile stationary points for the oxidized state [Fe4S4]+2 and protonated Glu126. Spin densities are represented by transparent spheres (α: green and β: purple), the iso-surfaces are resolved at 0.03 a.u. Key bond distances also are reported in Å.

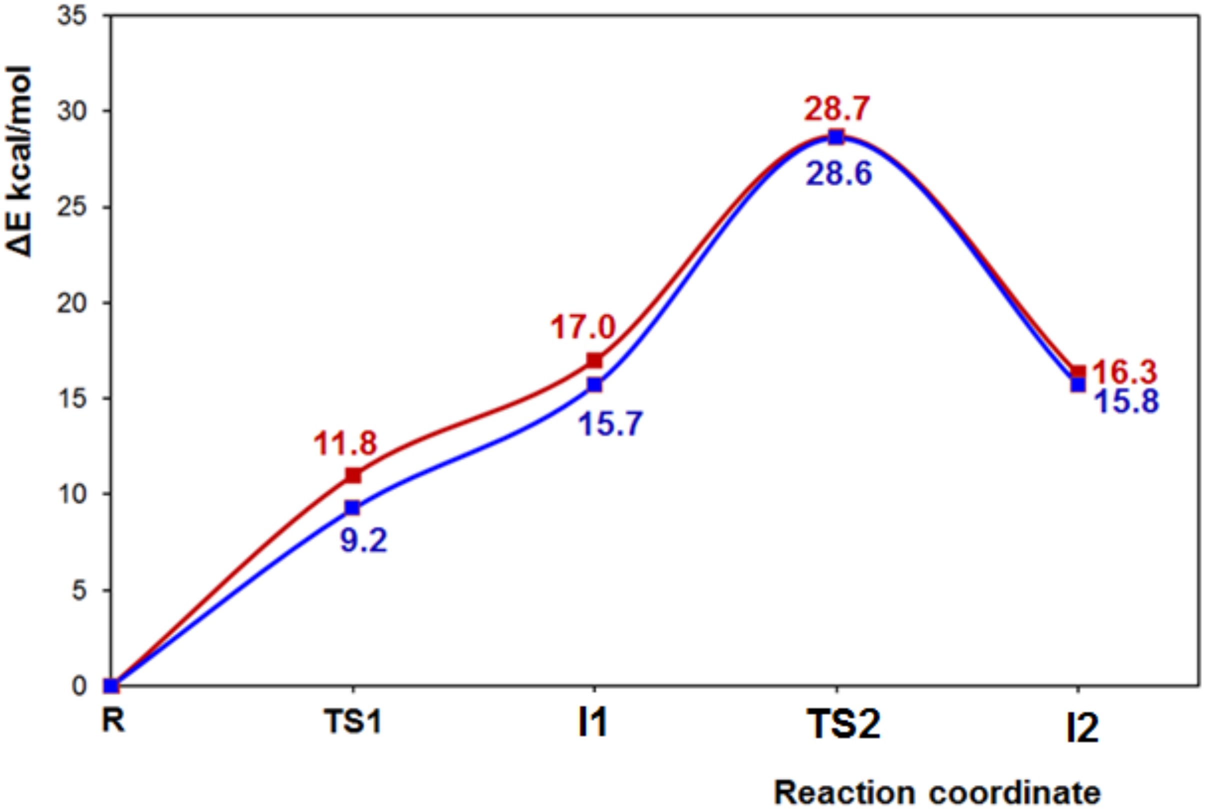

Figure S13. Reaction profile of the 4-OH Rotation and elimination step calculated at ONIOM (B3LYP/TZVP:Amber)-EE using M2 (red) and DFT+U (blue) using M3.

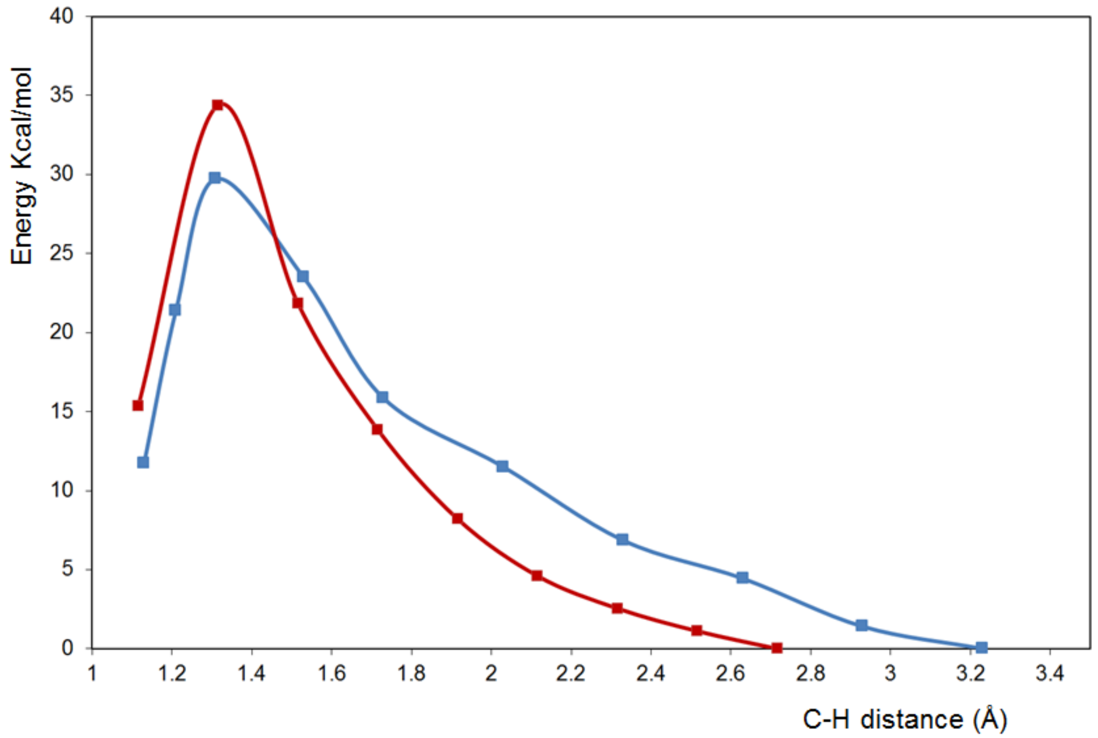

Figure S14. Relaxed scans of the C-H bond distance to simulate the protonation at C2 (blue) and at C4 (red). Relaxed scans are calculated using model M1 and ONIOM-MM (B3LYP/TZVP:Amber) level of theory.

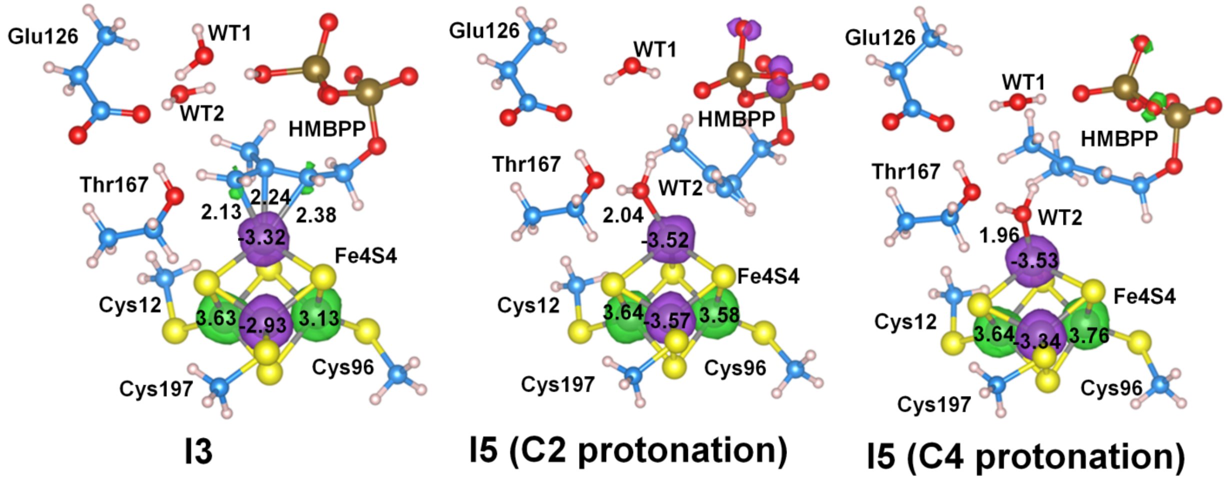

Figure S15. Balls and sticks representation of the reaction profile stationary points of the 2e-/H+ transfer step calculated using the reduced state $[Fe_4S_4]^+$. Spin densities are represented by transparent spheres (α: purple and β: green), the iso-surfaces are at 0.03 a.u. Key bond distances also are reported in Å.

Table S1. ONIOM optimized parameters of IspH calculated at B3LYP/TZVP:AMBER level using different BS states and the oxidized state $[Fe_4S_4]^{2+}$. Values in parenthesis are calculated using ONIOM-EE B3LYP/TZVP: AMBER level of theory.

Cluster distance	BS1	BS2	BS3	BS4	BS5	BS6	Exp.
Fe1-O	2.03(2.02)	2.01	2.02	2.03	2.01	2.02	2.10
Fe1-S1	2.39(2.37)	2.41	2.31	2.39	2.39	2.31	2.35
Fe1-S2	2.30(2.29)	2.40	2.37	2.30	2.38	2.37	2.40
Fe1-S4	2.44(2.42)	2.48	2.43	2.44	2.32	2.43	2.38
Fe2-S1	2.33(2.33)	2.39	2.34	2.33	2.32	2.34	2.22
Fe2-S2	2.31(2.33)	2.42	2.30	2.31	2.30	2.30	2.19
Fe2-S3	2.34(2.36)	2.43	2.37	2.34	2.33	2.36	2.18
Fe3-S1	2.33(2.32)	2.35	2.38	2.33	2.31	2.39	2.32
Fe3-S3	2.34(2.35)	2.39	2.39	2.34	2.35	2.39	2.31
Fe3-S4	2.32(2.34)	2.40	2.32	2.32	2.33	2.32	2.28
Fe4-S2	2.37(2.38)	2.31	2.31	2.37	2.30	2.31	2.32
Fe4-S3	2.42(2.41)	2.42	2.34	2.42	2.44	2.34	2.28
Fe4-S4	2.34(2.35)	2.35	2.33	2.34	2.42	2.33	2.21
Fe2-SC197	2.27(2.26)	2.27	2.27	2.27	2.27	2.27	2.28
Fe3-Sc96	2.28(2.27)	2.28	2.29	2.28	2.28	2.29	2.27
Fe4-Sc12	2.32(2.31)	2.31	2.31	2.32	2.32	2.31	2.26
Fe1-Fe2	2.84(2.84)	3.06	2.83	2.84	2.87	2.83	2.80
Fe2-Fe3	2.78(2.83)	2.77	2.76	2.78	2.81	2.76	2.71
Fe2-Fe4	2.90(2.87)	3.09	2.87	2.90	2.90	2.87	2.70
Fe1-Fe3	2.78(2.73)	3.06	2.86	2.78	2.81	2.76	2.77
Fe1-Fe4	2.90(2.91)	2.92	2.85	2.90	2.90	2.86	2.82
Fe3-Fe4	2.91(2.93)	3.10	2.89	2.90	2.97	2.89	2.74

Table S2. ONIOM-EE relative energy of the BS states calculated at B3LYP/TZVP: AMBER level using the reduced [Fe4S4]+ and the oxidized [Fe4S4]2+ states.

BS-states	$[Fe_4S_4]^+$	$[Fe_4S_4]^{2+}$
1	0.0	0.0
2	1.40	12.40
3	0.0	0.0
4	0.0	0.0
5	4.40	5.73
6	0.0	0.0

Table S3. ONIOM optimized parameters of IspH calculated at B3LYP/TZVP:AMBER level using the most stable BS state and the reduced state [Fe4S4]+.

Cluster distance	BS1	Exp.
Fe1-O	2.09	2.10
Fe1-S1	2.25	2.35
Fe1-S2	2.36	2.40
Fe1-S4	2.39	2.38
Fe2-S1	2.40	2.22
Fe2-S2	2.37	2.19
Fe2-S3	2.46	2.18
Fe3-S1	2.48	2.32
Fe3-S3	2.40	2.31
Fe3-S4	2.39	2.28
Fe4-S2	2.33	2.32
Fe4-S3	2.32	2.28
Fe4-S4	2.35	2.21
Fe2-SC197	2.26	2.28
Fe3-Sc96	2.30	2.27
Fe4-Sc12	2.35	2.26
Fe1-Fe2	2.84	2.80
Fe1-Fe3	3.06	2.71
Fe1-Fe4	2.92	2.70
Fe2-Fe3	2.81	2.77
Fe2-Fe4	2.74	2.82
Fe3-Fe4	2.89	2.74

Table S4. Natural Population Analysis charges and spin densities calculated for the QM region using model M2 (160 atoms) using B3LYP/TZVP calculated for the reduced state and the protonated Glu126. Note: for INT1, TS2 and INT2 the charge of the transferred proton from WT1 is summed to HMBPP substrate. Protein is all the amino acids and water molecules except the three cysteinates bound to Fe4S4 cluster and WT1.

	R		TS1		I1		TS2		I2	
Atom	Charge	Spin	charge	Spin	Charge	Spin	Charge	Spin	Charge	Spin
4-O	-0.77409	0.04363	-0.75551	0.00827	-0.8349	-0.02906	-0.75012	0.00005	-0.98539	-0.00296
4-H	0.52631	0.00007	0.51093	-0.00024	0.52561	0.00048	0.53724	0.00004	0.48704	0.00011
Hb3	0.51904	-0.00005	0.52439	0.00002	0.50427	0.00037	0.53917	-0.00001	0.48343	0.0001
C4	-0.08902	0.00061	-0.0949	0.00038	-0.09701	-0.06873	-0.09397	0.00111	-0.42213	-0.22934
H41	0.15373	0.00145	0.14323	0.0005	0.22056	0.00216	0.15997	-0.00024	0.24216	0.00752
H42	0.18026	0.00077	0.17456	0.00008	0.23422	0.00145	0.21231	0.00061	0.25207	0.00925
C3	-0.0243	0.00316	-0.00778	0.0039	-0.21775	0.01373	-0.13065	-0.00031	-0.07995	0.08833
C2	-0.18877	-0.00132	-0.20681	-0.00116	-0.2222	-0.15525	-0.30244	-0.04381	-0.11543	-0.25619
H21	0.21753	-0.00013	0.21749	-0.00023	0.24219	0.00533	0.24557	0.00205	0.23621	0.00896
C1	-0.10889	0.00017	-0.10653	0.00016	-0.11364	0.00654	-0.10391	0.00255	-0.13572	0.00997
H11	0.20484	0.00011	0.20183	0.00012	0.21099	-0.00586	0.21926	-0.00093	0.21845	-0.01149
H12	0.18254	0	0.17969	0.00002	0.18962	-0.00099	0.18428	-0.00049	0.18845	-0.00182
C5	-0.6467	-0.00017	-0.64982	-0.00019	-0.63058	-0.00064	-0.63528	0.00083	-0.65604	-0.00515
H51	0.24061	0.00001	0.24431	0.00011	0.23348	-0.00052	0.20684	-0.0026	0.24393	0.00193
H52	0.231	0.00012	0.22738	0.00016	0.23071	0.00032	0.23935	0.00011	0.23202	0.00476
H53	0.18237	0.00011	0.18182	0.00006	0.20155	-0.00002	0.20623	-0.0001	0.22987	0.0007
Oa1	-0.78423	0	-0.78575	0	-0.77917	-0.00585	-0.78379	-0.00272	-0.77042	-0.00825
Pa	2.44752	0	2.44887	0	2.44975	-0.00003	2.44627	-0.00011	2.4467	0.00012
Oab	-1.08815	0	-1.08415	0	-1.09476	-0.00044	-1.08187	-0.00005	-1.09985	-0.00564
Pb	2.55002	0	2.55091	0	2.54107	0.00002	2.55122	0.00003	2.5505	-0.00007
Ob3	-1.03397	0	-1.03327	0	-1.01578	-0.00036	-0.93246	0	-1.03282	-0.00005
Ob2	-1.20096	0	-1.20403	0	-1.18692	-0.00006	-1.16847	0	-1.18901	-0.00062
Ob1	-1.14205	0	-1.14513	0	-1.16458	-0.00002	-1.13817	0.00001	-1.17693	-0.00004
Oa2	-1.13334	0	-1.13628	0	-1.12813	-0.00009	-1.11203	0.00001	-1.12749	-0.00017
Oa3	-0.77409	0.04363	-0.75551	0.00827	-0.8349	-0.02906	-0.75012	0.00005	-0.98539	-0.00296
HMBPP	-1.72927	0.04854	-1.7571	0.01196	-1.52021	-0.20994	-1.41583	-0.04424	-1.5822	-0.38811
Fe1	1.056	-3.42423	1.05327	-3.42531	1.02159	-3.38015	1.04075	-3.4133	1.01414	-3.3557
Fe2	1.12109	3.512	1.08835	3.50926	1.12586	3.42255	1.11838	3.42483	1.13395	3.40577
Fe3	1.12024	3.61119	1.1255	3.6282	1.17825	3.68248	1.18236	3.68167	1.17589	3.68406
Fe4	1.10734	-3.48442	1.10449	-3.48621	1.08811	-3.45493	1.09861	-3.46841	1.0735	-3.42994
S1	-1.07948	0.30579	-1.07269	0.31257	-1.03164	0.30488	-1.07968	0.26832	-0.99507	0.3388
S2	-1.20879	0.24887	-1.19563	0.25272	-1.04183	0.29997	-1.13388	0.27325	-0.93603	0.29176
S3	-1.07008	0.13547	-1.06691	0.12566	-0.98764	0.20912	-1.07455	0.11331	-0.91913	0.28178
S4	-1.08001	0.07359	-1.06672	0.08779	-1.01966	0.15344	-1.03596	0.16013	-1.00143	0.15652
Fe4S4(SCH3)3	-2.02862	0.94714	-1.98927	0.98374	-1.54384	1.23667	-1.84058	1.0426	-1.24468	1.38822
Protein	-0.24214	0.00435	-0.25359	0.00432	-1.13093	0.0015	-1.06991	0.00155	-1.1582	0.00263

Table S5. Natural Population Analysis charges and spin densities calculated for the QM region using model M2 (160 atoms) using B3LYP/TZVP calculated for the reduced state and the deprotonated Glu126. Note: for INT1, TS2 and INT2 the charge of the transferred proton from WT1 is summed to HMBPP substrate. Protein is all the amino acids and water molecules except the three cysteinates bound to Fe_4S_4 cluster. .

	R		TS1		I1		TS2		I2	
Atom	Charge	Spin	charge	Spin	Charge	Spin	Charge	Spin	Charge	Spin
4-O	-0.78325	0.0521	-0.76063	0.01396	-0.89674	0.00085	-0.76206	0.00074	-1.00604	-0.00196
4-H	0.52701	0.00004	0.51454	-0.0003	0.49697	0.00004	0.52722	0.00002	0.48119	0.0001
Hb3	0.51228	-0.00004	0.51379	0	0.54452	-0.00001	0.53535	-0.00002	0.50455	0.00005
C4	-0.09906	0.00053	-0.09825	0.00031	-0.00711	0.00045	-0.09642	0.0008	-0.45308	-0.29085
H41	0.18626	0.0018	0.16395	0.00087	0.1959	0.00083	0.16375	0.0027	0.23252	0.00984
H42	0.17623	0.00093	0.16616	0.00011	0.2209	0.00104	0.19158	0.00087	0.25095	0.01045
C3	-0.02505	0.00303	-0.01372	0.00303	-0.21743	0.01299	-0.11356	0.02325	-0.06822	0.08655
C2	-0.19608	-0.00116	-0.20735	-0.0006	-0.23184	-0.04532	-0.25786	-0.03062	-0.14739	-0.2874
H21	0.21727	-0.00013	0.21878	-0.00015	0.27462	0.00189	0.24656	0.00131	0.23232	0.00837
C1	-0.10912	0.00013	-0.10669	0.00007	-0.10967	0.00252	-0.10864	0.00187	-0.12023	0.01187
H11	0.19695	0.0001	0.19371	0.00015	0.20698	-0.00117	0.20534	-0.00054	0.19761	-0.01008
H12	0.18249	0	0.17888	0.00001	0.17938	-0.00034	0.18113	-0.00025	0.1863	-0.00098
C5	-0.64208	-0.00018	-0.64226	-0.00025	-0.62955	0.00013	-0.64397	-0.00056	-0.6531	-0.0034
H51	0.23549	-0.00001	0.23386	0.00003	0.23692	-0.00083	0.22374	0.00032	0.24027	0.00183
H52	0.21917	0.0001	0.21932	0.00013	0.24437	0.0003	0.23718	0.00063	0.21297	0.00424
H53	0.18912	0.00014	0.18632	0.0001	0.19321	0.00003	0.19533	0.00013	0.24509	0.00124
Oa1	-0.78683	0	-0.7878	0.00003	-0.78144	-0.00276	-0.78445	-0.00182	-0.78375	-0.0119
Pa	2.45413	0	2.45426	-0.00001	2.45151	-0.00004	2.45117	-0.00005	2.45976	-0.00013
Oab	-1.09354	0	-1.08935	0.00001	-1.09205	-0.00002	-1.09083	0	-1.1091	-0.00179
Pb	2.54589	0	2.54803	0	2.55108	0	2.55093	0.00001	2.55974	-0.00743
Ob3	-1.03211	0	-1.03064	0.00001	-1.06629	0.00002	-1.05377	0.00002	-1.21334	0.00819
Ob2	-1.19665	0	-1.19763	0	-1.20435	0	-1.20149	0	-1.21897	0.04288
Ob1	-1.15071	0	-1.15407	0	-1.15919	0	-1.15848	0	-1.18412	0.09003
Oa2	-1.14752	0	-1.14922	0	-1.15424	0	-1.14797	0	-1.14907	0.00178
Oa3	-1.1569	-0.00001	-1.1588	-0.00001	-1.15626	-0.00023	-1.15794	-0.00015	-1.14079	0.00018
HMBPP	-1.77661	0.05737	-1.80481	0.0175	-1.9098	-0.02963	-2.16867	-0.00208	-2.44393	-0.33832
Fe1	1.06047	-3.43	1.05294	-3.42439	1.04299	-3.42129	1.05041	-3.4277	1.04058	-3.30196
Fe2	1.14586	3.53394	1.1168	3.52783	1.13404	3.45398	1.13583	3.46402	1.02198	3.17319
Fe3	1.1204	3.60978	1.11988	3.62117	1.18243	3.6814	1.17412	3.67203	1.14744	3.64227
Fe4	1.10571	-3.48465	1.10125	-3.48426	1.09202	-3.46573	1.09764	-3.47203	1.07194	-3.33613
S1	-1.07828	0.29976	-1.06689	0.30403	-1.05493	0.2643	-1.0712	0.27066	-0.97561	0.29953
S2	-1.16986	0.259	-1.14917	0.27008	-1.09944	0.27634	-1.10921	0.27404	-0.88771	0.31281
S3	-1.07378	0.1195	-1.06914	0.11497	-1.09722	0.08926	-1.1065	0.0741	-0.94515	0.16087
S4	-1.11314	0.06073	-1.10014	0.07152	-1.06748	0.13785	-1.07373	0.13648	-0.98261	0.23372
Fe4S4(SCH3)3	-2.05631	0.93908	-2.01479	0.97951	-1.88303	1.02841	-1.93384	0.99972	-1.28403	1.33218
protein	-1.16712	0.00351	-1.18043	0.00302	-1.20716	0.00124	-1.198	0.00163	-1.27199	0.00606

Table S6. Natural Population Analysis charges and spin densities calculated for the QM region using model M2 (160 atoms) using B3LYP/TZVP calculated for the oxidized state and the protonated Glu126. Note: for INT1, TS2 and INT2 the charge of the transferred proton from WT1 is summed to HMBPP substrate. Protein is all the amino acids and water molecules except the three cysteinates bound to Fe4S4 cluster.

	R		TS1		I1		TS2		I2	
Atom	Charge	Spin	charge	Spin	Charge	Spin	Charge	Spin	Charge	Spin
4-O	-0.78616	0.04907	-0.75937	0.01003	-0.74919	0.00032	-0.7982	-0.02005	-0.97514	-0.00383
4-H	0.53156	-0.00029	0.52605	-0.00029	0.53643	0	0.52998	0.00039	0.48365	0.00012
Hb3	0.50991	0	0.52407	0.00003	0.54099	0	0.50244	0.00031	0.48595	0.00013
C4	-0.09139	0.00059	-0.12534	-0.00146	-0.11059	-0.00136	-0.08065	-0.02866	-0.4206	-0.17239
H41	0.16929	0.00156	0.17661	0.00629	0.17519	0.00434	0.24353	0.00082	0.24802	0.00747
H42	0.17705	0.00093	0.19021	0.00247	0.21386	0.00238	0.22584	0.00029	0.27775	0.00849
C3	-0.01625	0.01321	0.00235	0.05813	-0.02454	0.05572	-0.20558	0.00714	-0.03413	0.06827
C2	-0.20135	-0.00514	-0.30398	-0.02049	-0.32873	-0.02957	-0.16981	-0.04407	-0.15071	-0.18934
H21	0.20944	0.00004	0.25007	0.00317	0.25595	0.00251	0.24931	0.00293	0.25555	0.00829
C1	-0.10552	0.00039	-0.10461	0.00205	-0.10451	0.0035	-0.12216	0.00287	-0.12468	0.00911
H11	0.19589	0.00011	0.20726	-0.00036	0.20995	-0.00067	0.21082	-0.00255	0.216	-0.00745
H12	0.18347	0.00005	0.18274	-0.00017	0.18795	-0.00024	0.19452	-0.00054	0.19775	-0.00081
C5	-0.65072	-0.0007	-0.6624	-0.00272	-0.64517	-0.00252	-0.62571	-0.00047	-0.65942	-0.00401
H51	0.25777	0.00019	0.25208	0.0022	0.22121	0.00374	0.22995	0.00032	0.26119	0.00162
H52	0.2229	0.0012	0.24279	0.00285	0.23532	0.00066	0.22432	0.00052	0.23085	0.00388
H53	0.19049	0.00018	0.19607	0.00008	0.21656	0.00113	0.21742	0.00029	0.23821	0.00047
Oa1	-0.77912	-0.00015	-0.77383	-0.00036	-0.77101	-0.00014	-0.76472	-0.00067	-0.76578	-0.0087
Pa	2.44572	0	2.45	-0.00003	2.44366	-0.00003	2.44526	-0.00002	2.45191	0.00036
Oab	-1.08995	0.00002	-1.10577	0.00008	-1.09318	-0.00005	-1.10623	-0.00041	-1.11404	-0.00271
Pb	2.54975	0	2.55546	0.00002	2.55192	-0.00001	2.53929	0.00004	2.56803	0.00016
Ob3	-1.03348	0.00001	-1.03027	0.0001	-0.93079	0.00002	-1.00994	-0.00022	-1.06126	-0.00007
Ob2	-1.19493	0	-1.1887	0.00002	-1.15767	-0.00001	-1.17708	-0.00004	-1.21841	-0.00092
Ob1	-1.14541	0	-1.16747	0.00001	-1.13529	0	-1.15859	-0.00002	-1.2235	-0.00001
Oa2	-1.12951	0	-1.12412	0.00001	-1.10498	0.00005	-1.11725	0	-1.1045	-0.00016
Oa3	-1.14186	-0.00003	-1.13403	0	-1.1268	0.00005	-1.12317	-0.00005	-1.10732	-0.00205
4-O	-0.78616	0.04907	-0.75937	0.01003	-0.74919	0.00032	-0.7982	-0.02005	-0.97514	-0.00383
HMBPP	-1.72241	0.06124	-1.72413	0.06166	-0.95458	0.03982	-1.12622	-0.08184	-1.50868	-0.28408
Fe1	1.03733	-3.49301	1.03496	-3.48308	1.0189	-3.46373	0.99596	-3.419	0.98953	-3.39856
Fe2	1.17915	3.53393	1.18218	3.53011	1.12361	3.45611	1.12871	3.42967	1.14495	3.40406
Fe3	1.1665	3.67434	1.16327	3.66661	1.16655	3.67981	1.17106	3.68935	1.17204	3.6954
Fe4	1.08319	-3.53829	1.10179	-3.55979	1.08655	-3.54424	1.10012	-3.56394	1.0777	-3.53609
S1	-0.99379	0.21254	-0.97866	0.24213	-0.95878	0.23054	-0.9465	0.26551	-0.87125	0.30062
S2	-1.09556	0.17598	-1.04664	0.15979	-1.02335	0.16349	-0.95412	0.15418	-0.85115	0.18843
S3	-0.92975	-0.227	-0.94674	-0.23333	-0.9375	-0.22797	-0.90186	-0.14676	-0.81907	-0.08238
S4	-0.92147	-0.16207	-0.91673	-0.14334	-0.89286	-0.12644	-0.87771	-0.12016	-0.85242	-0.11246
Fe4S4(SCH3)3	-1.09197	-0.06199	-1.06073	-0.06169	-1.00742	-0.03975	-0.78625	0.0815	-0.36256	0.28213
protein	-0.18558	0.00075	-0.21513	-1E-05	-1.03797	-0.0001	-1.08748	0.00033	-1.12877	0.00199

Table S7. Frontier molecular orbitals of **I2 EH126** and reduced state.

Occupied Molecular Orbitals	Virtual Molecular Orbitals
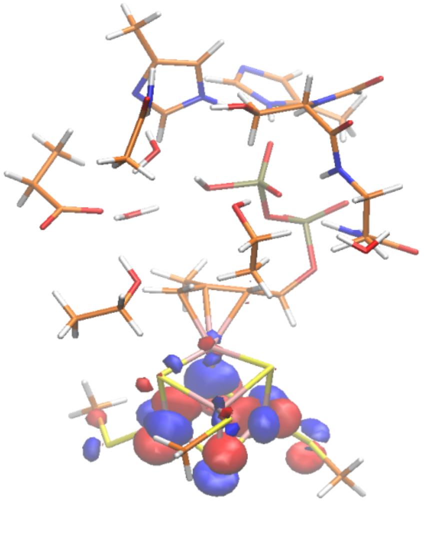<br>SOMO<br>MO_391_A=2.09	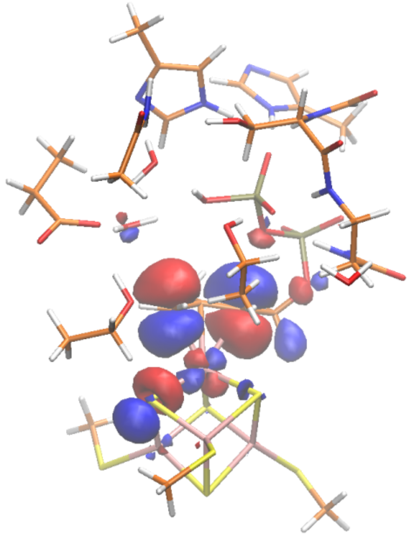<br>LUMO<br>MO_391_B=3.87
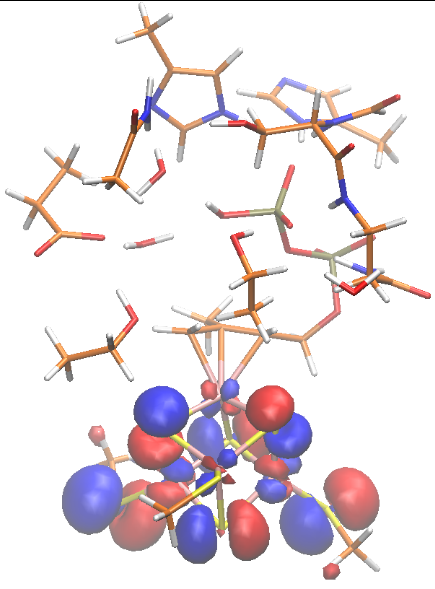<br>SOMO-1<br>MO_390_B=2.08	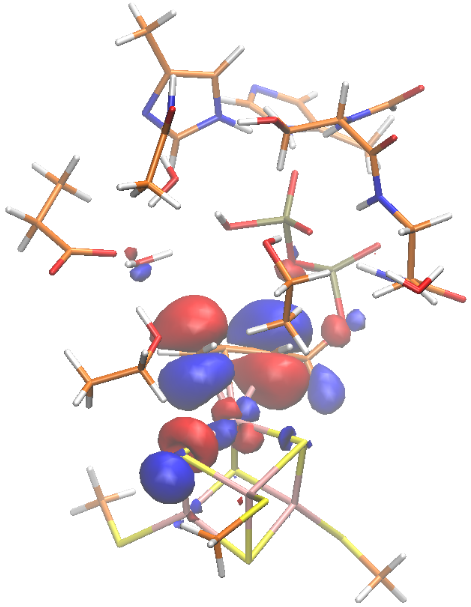<br>LUMO+1<br>MO_392_A=4.05

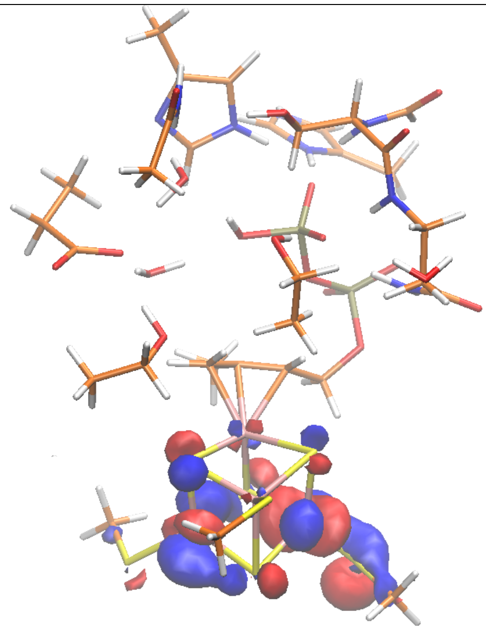

SOMO-2
MO_390_A=1.97

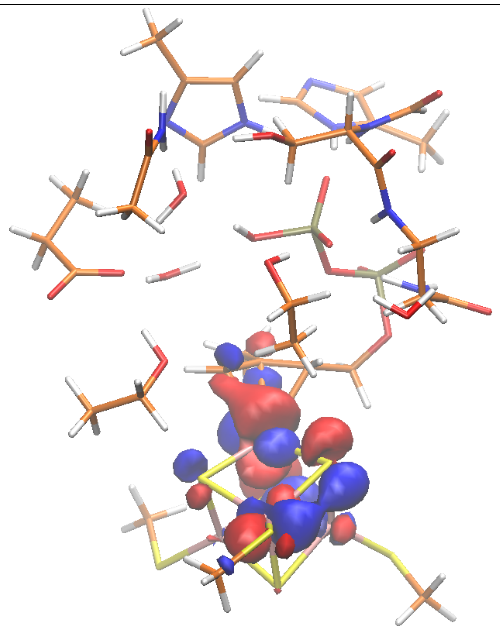

LUMO+2
MO_392_B=4.16

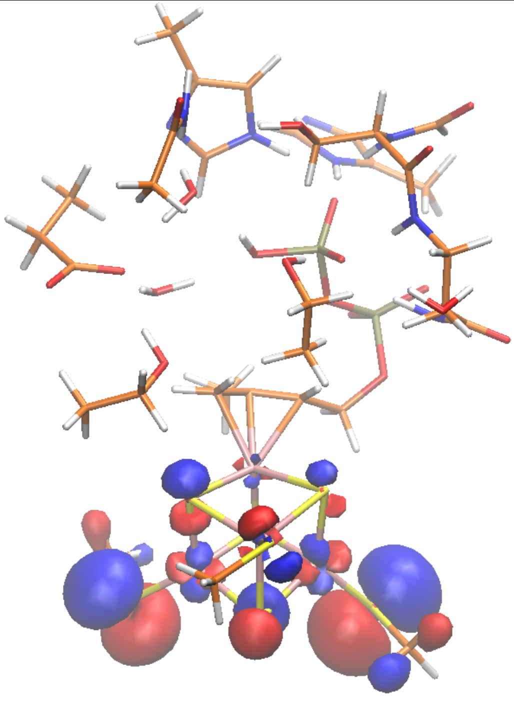

SOMO-3
MO_389_B=1.90

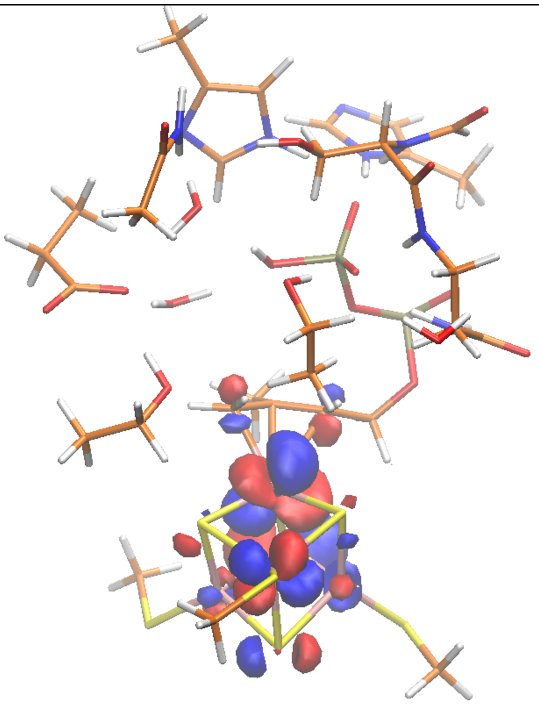

LUMO+3
MO_393_B=4.39

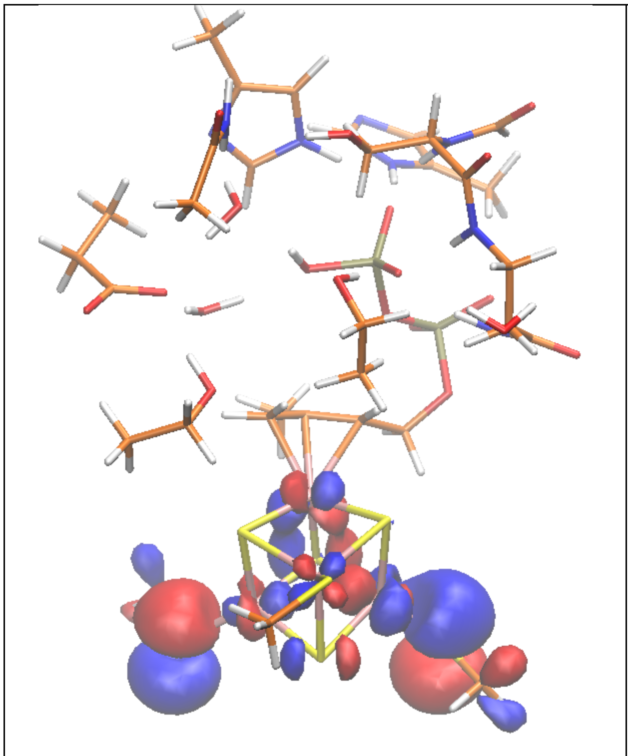

SOMO-4
MO_388_B=1.60

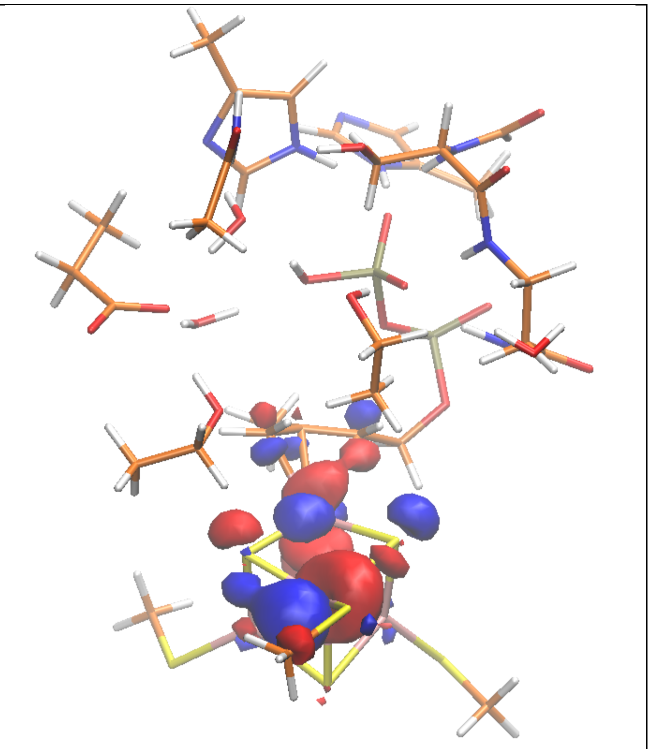

LUMO+4
MO_294_B=4.43

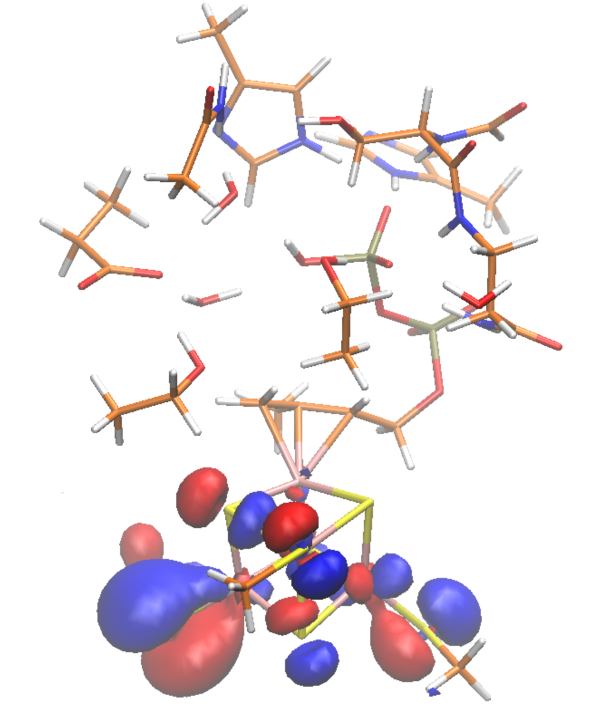

SOMO-5
MO_389_A=0.0571623382

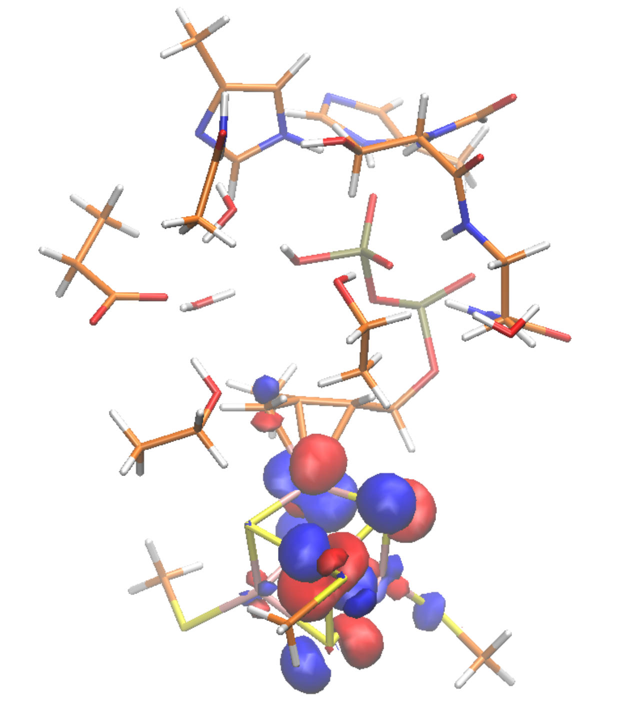

LUMO+5
MO_395_B=0.1717555030

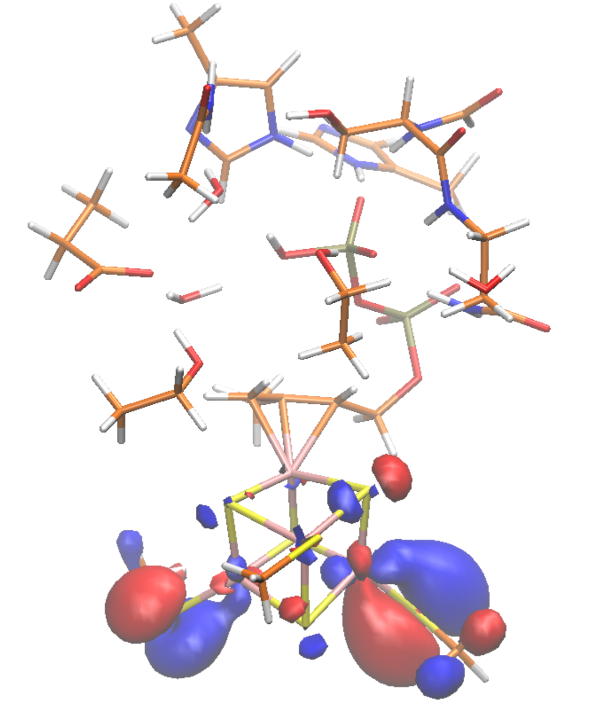

SOMO-6
MO_388_A=1.51

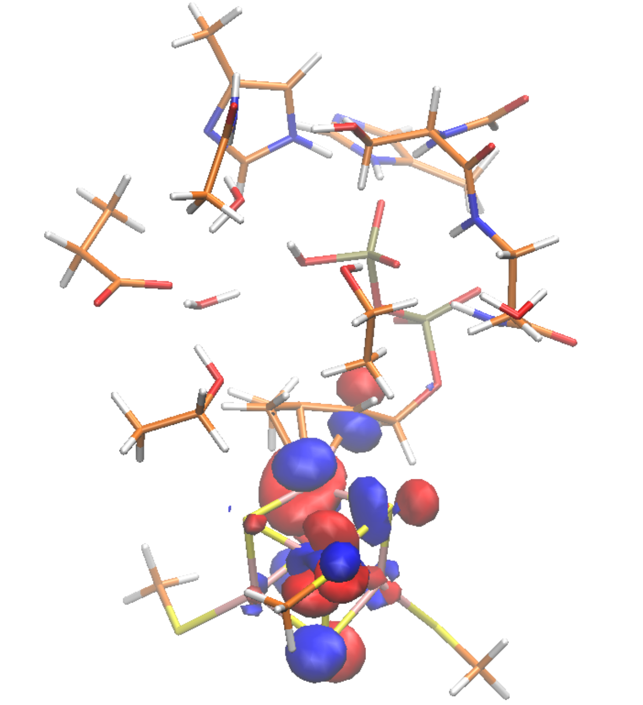

LUMO+6
MO_396_B=4.76

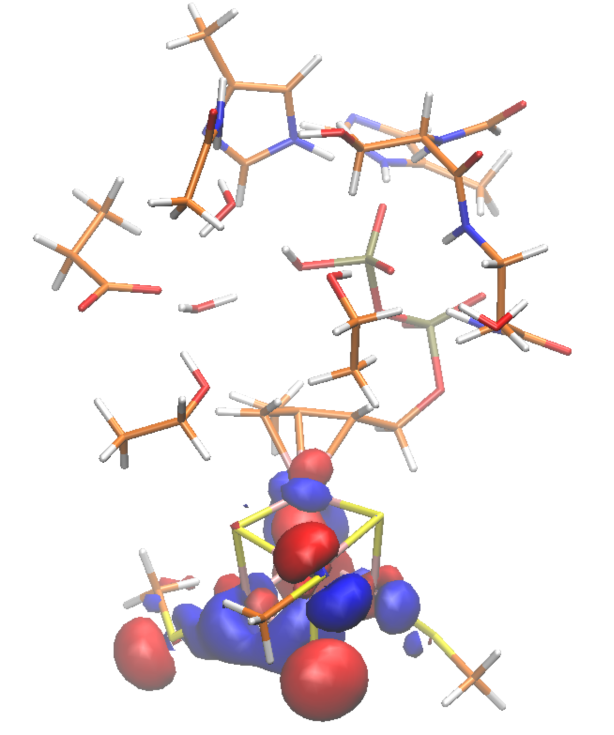

SOMO-7
MO_387_B=1.42

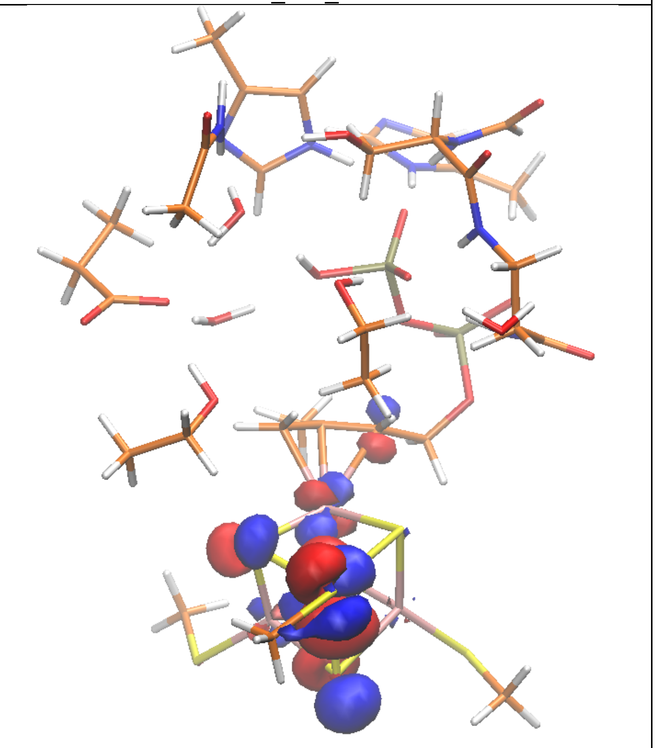

LUMO+7
MO_397_B=4.80

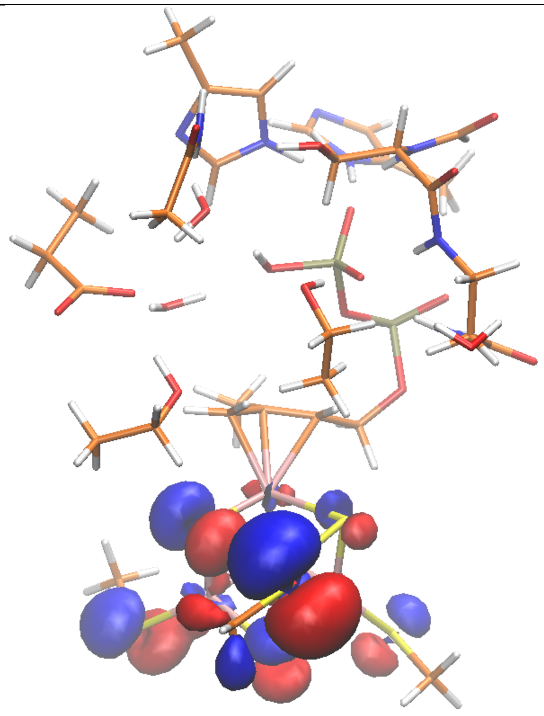

SOMO-8
MO_387_A=1.28

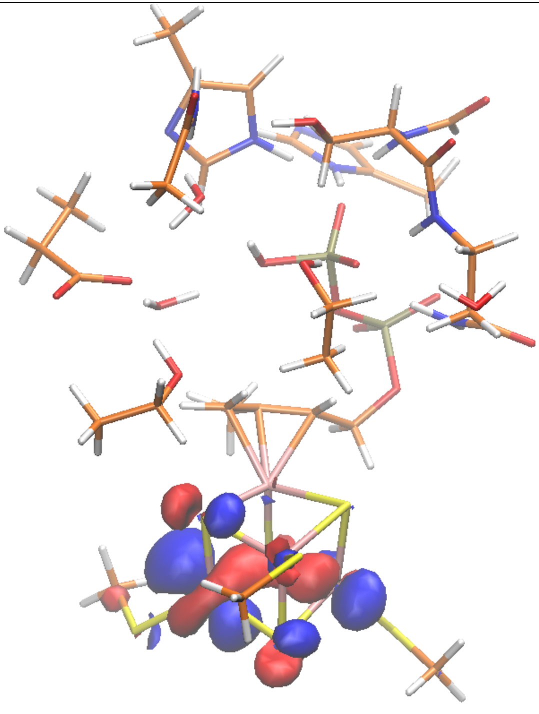

LUMO+8
MO_397_A=4.95

Table S8. Frontier molecular orbitals of **I2 EH126** and the oxidized state.

Occupied Molecular Orbitals	Virtual Molecular Orbitals
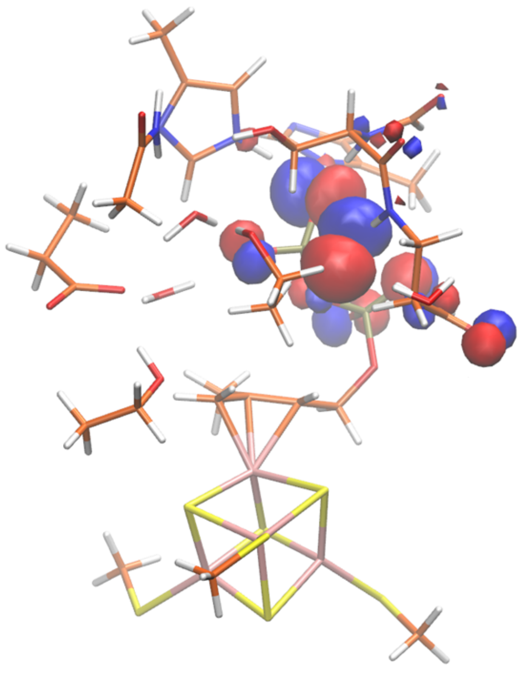 SOMO MO_390_B=-0.26	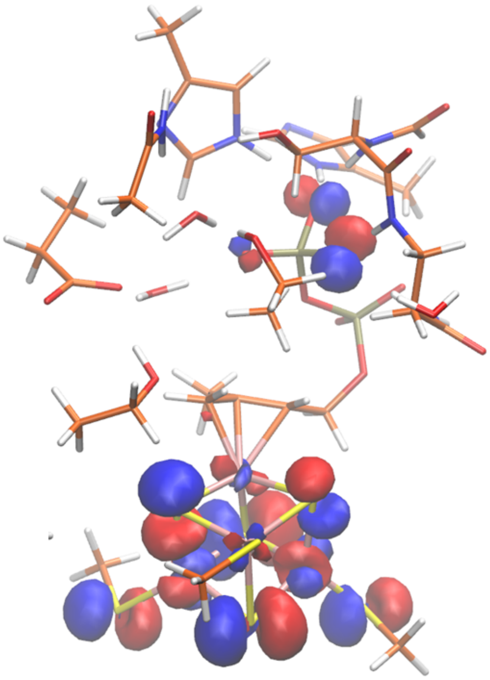 LUMO MO_391_B=0.21
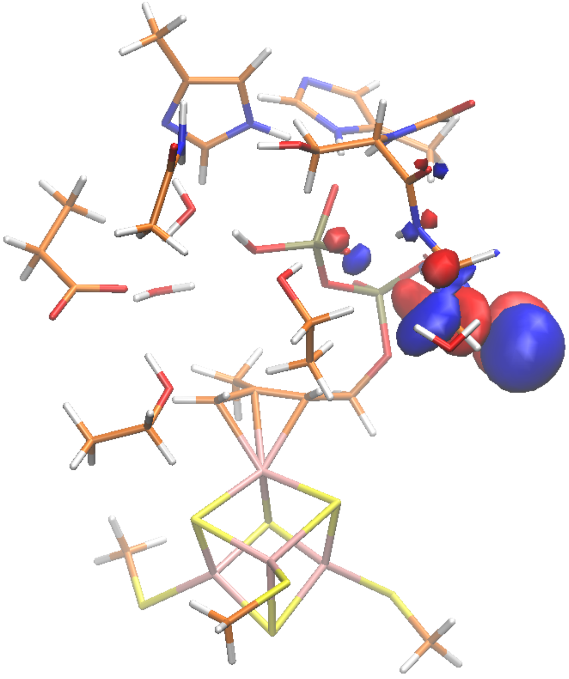 SOMO-1 MO_390_A=-0.26	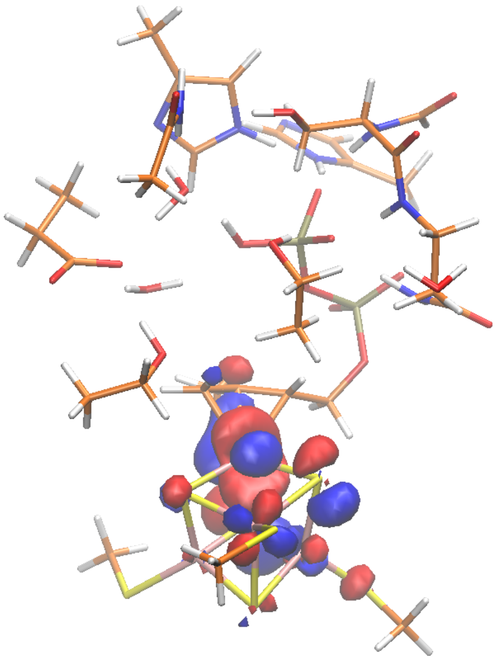 LUMO+1 MO_392_B=0.48

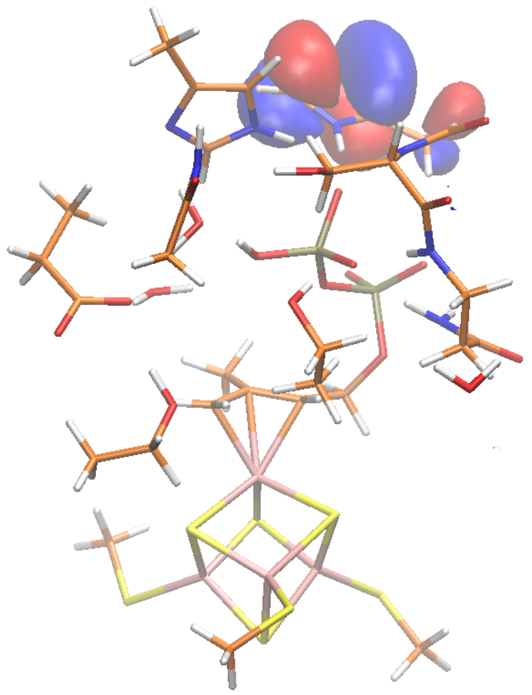

SOMO-2
MO_389_A=-0.46

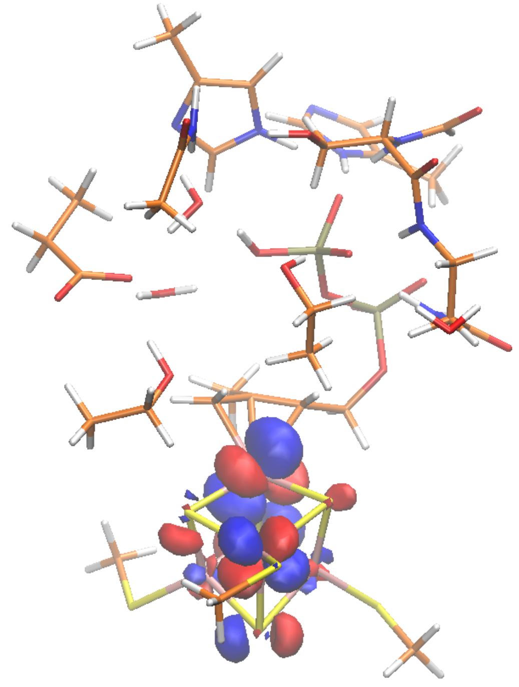

LUMO+2
MO_393_B=0.68

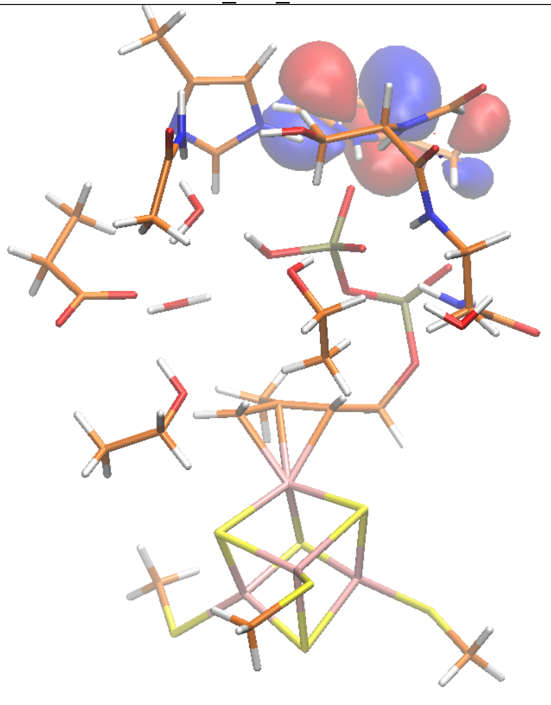

SOMO-3
MO_389_B=-0.46

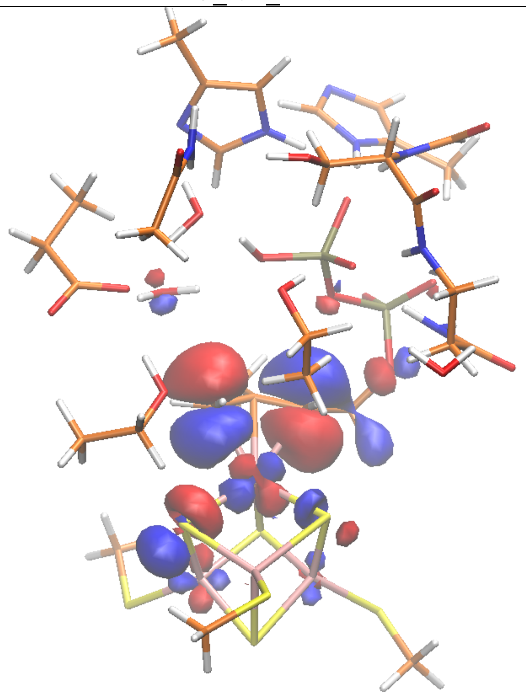

LUMO+3
MO_391_A=0.83

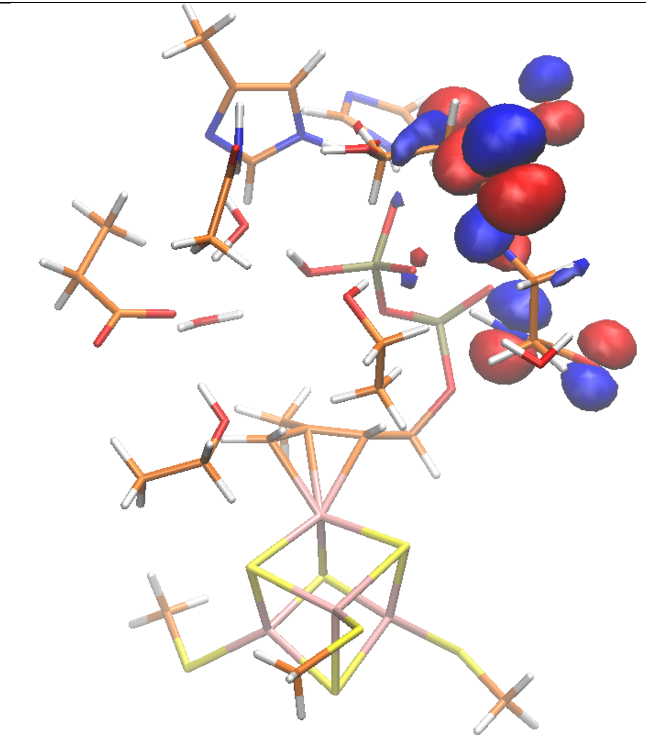

SOMO-4
MO_388_B=-0.51

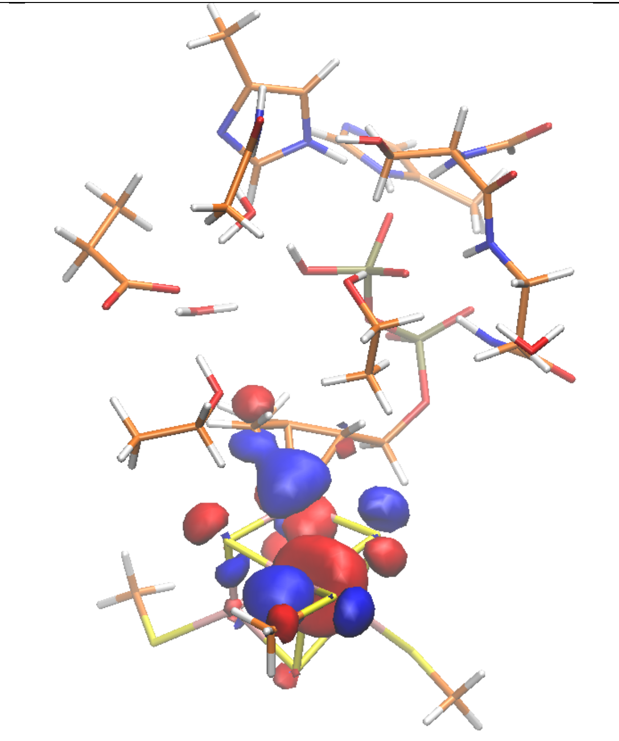

LUMO+4
MO_394_B=0.91

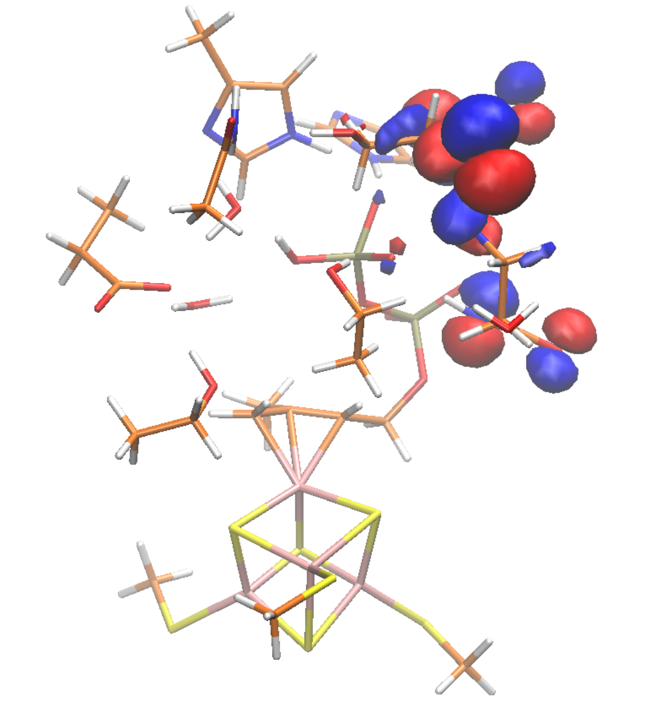

SOMO-5
MO_388_A=-0.51

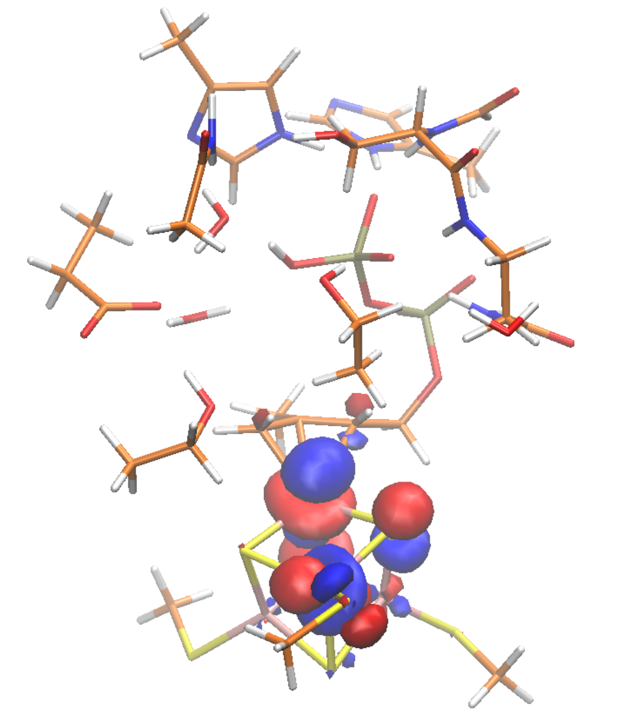

LUMO+5
MO_395_B=1.96

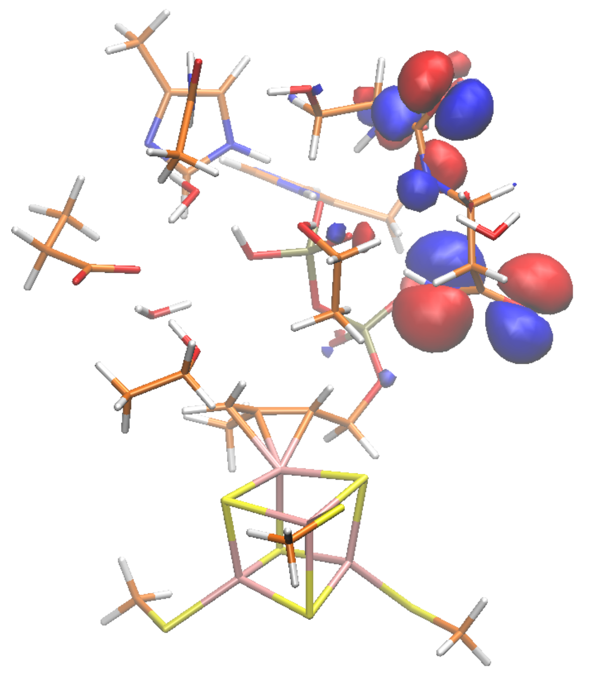

SOMO-6
MO_387_B=-0.63

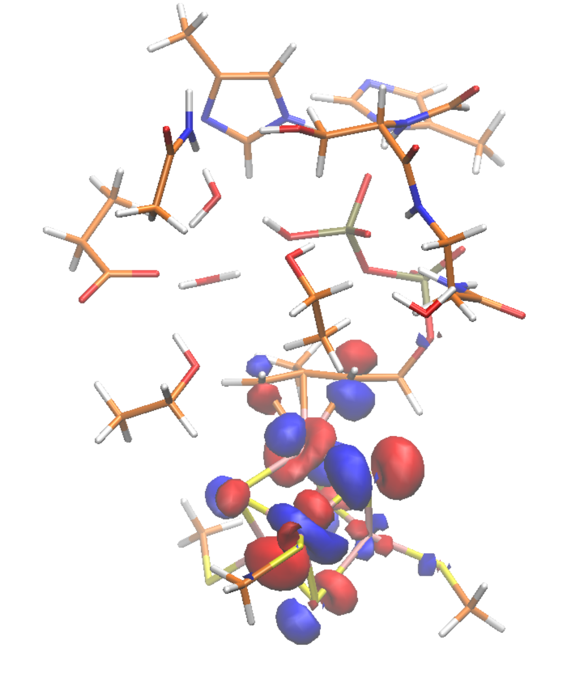

LUMO+6
MO_396_B=1.12

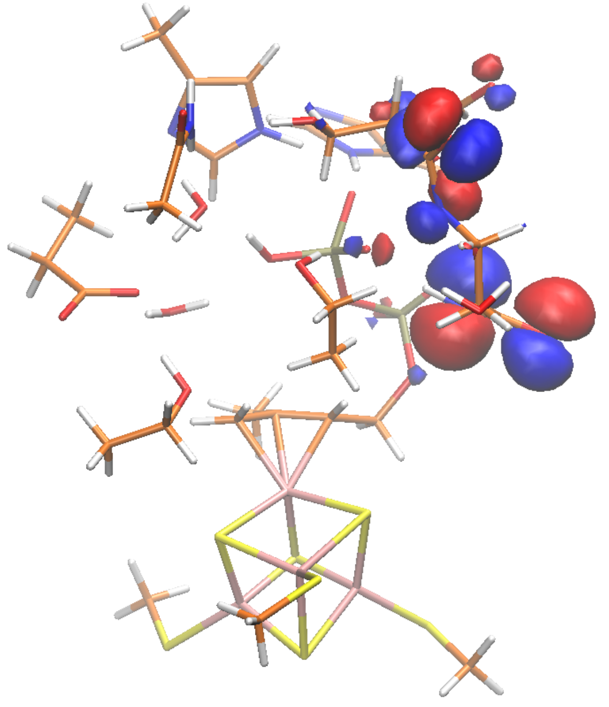

SOMO-7
MO_387_A=-0.63

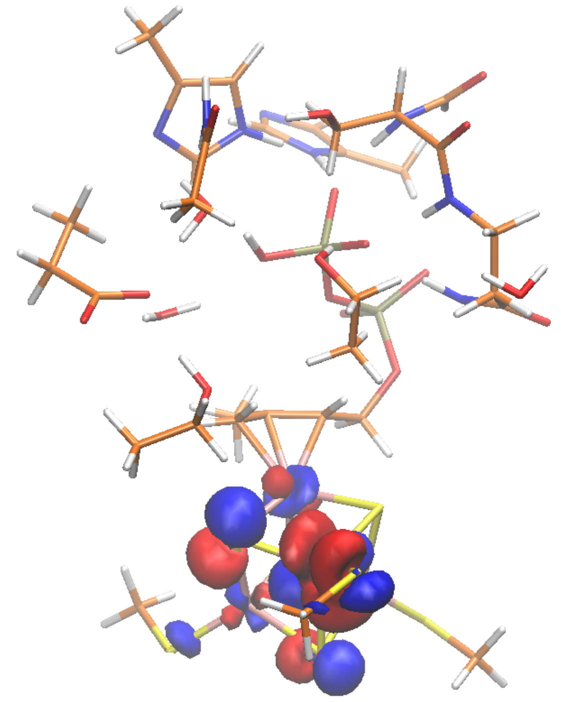

LUMO+7
MO_397_B=1.25

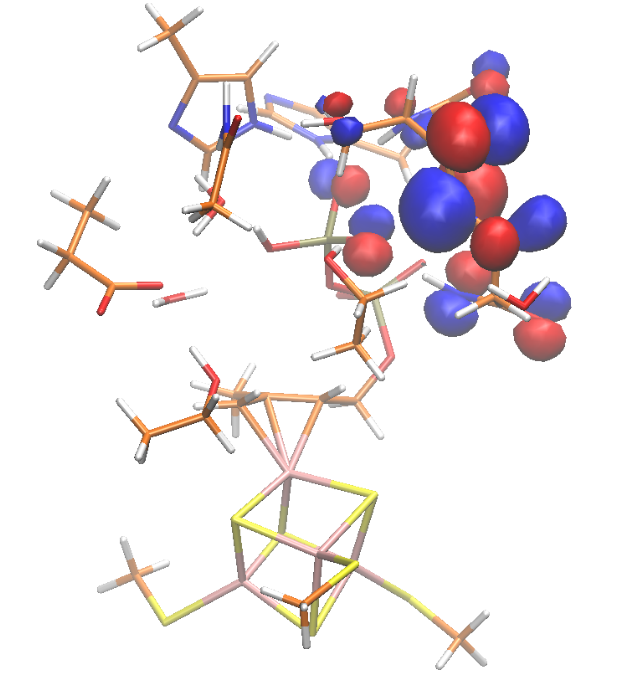

SOMO-8
MO_386_B=-0.81

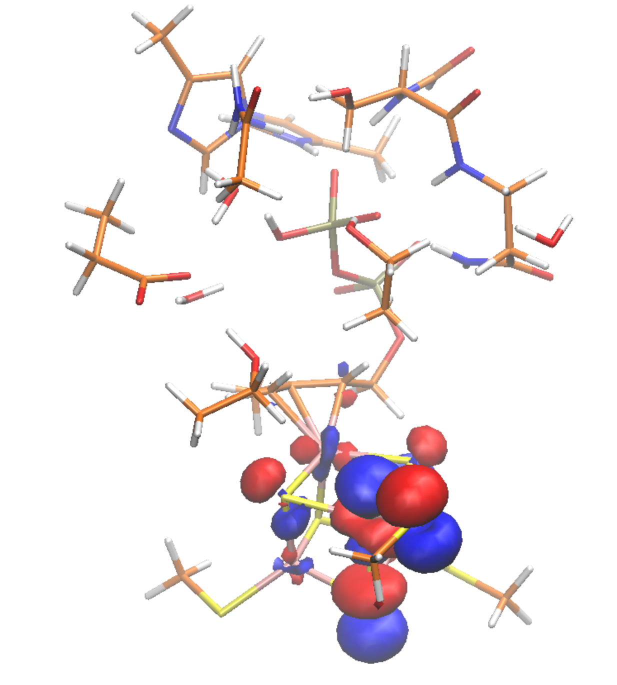

LUMO+8
MO_398_B=1.33

Table S9. Frontier molecular orbitals of **I2 E126** and the reduced state.

Occupied Molecular Orbitals	Virtual Molecular Orbitals
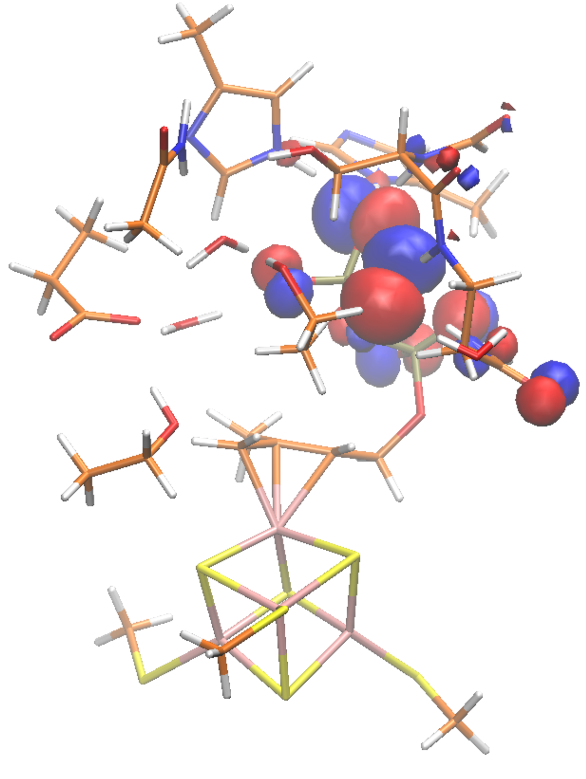<br>SOMO<br>MO_385_B=4.01	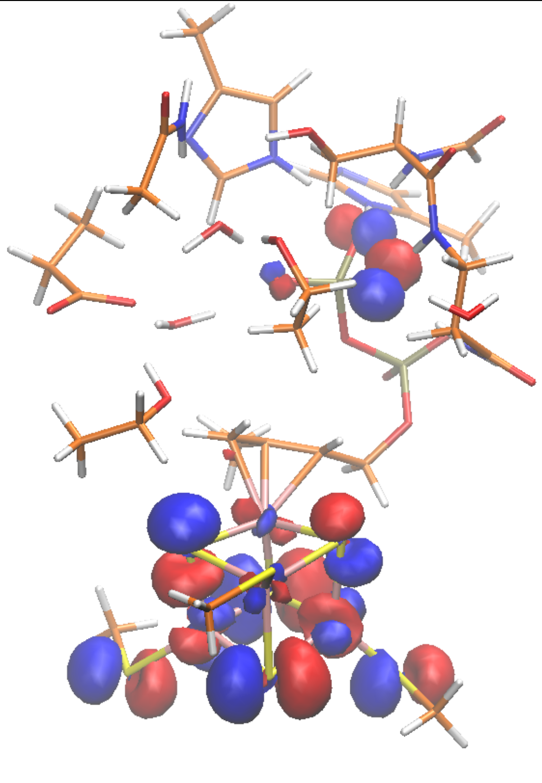<br>LUMO<br>MO_386_B=4.34
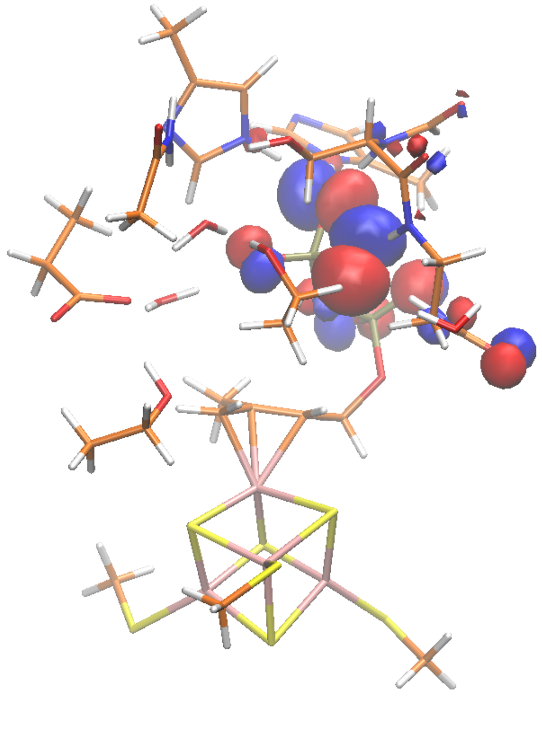<br>SOMO-1	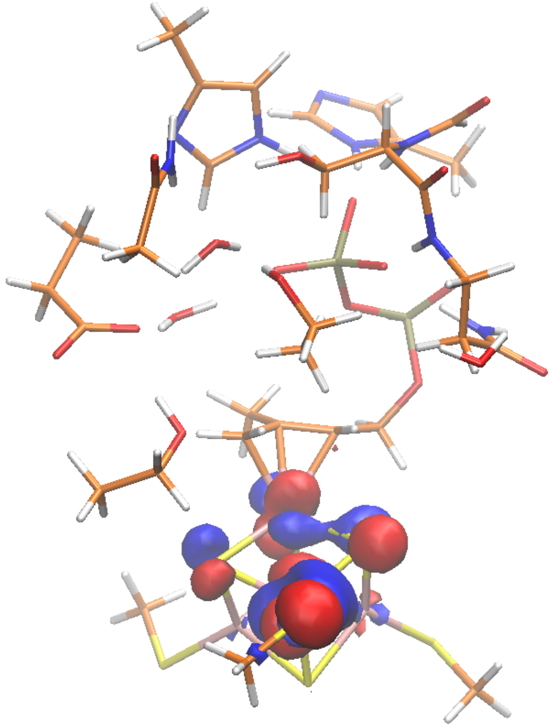<br>LUMO+1

MO_386_A=3.57	MO_387_B=6.13
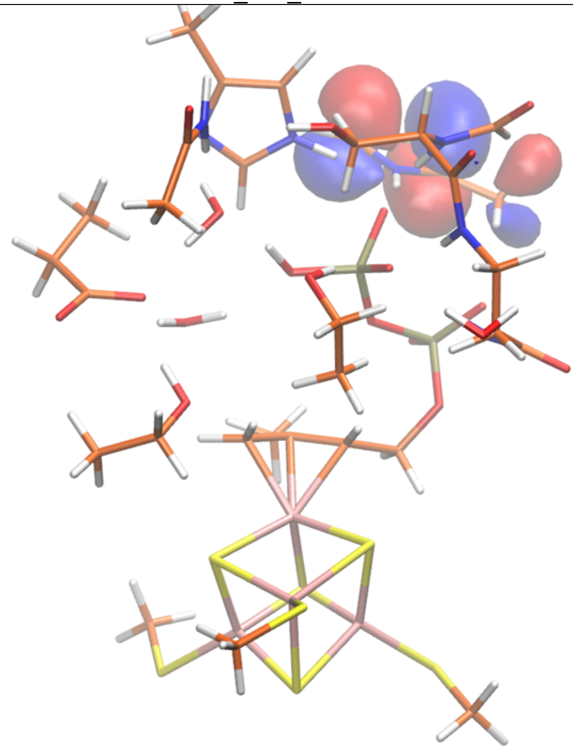<br>SOMO-2<br>MO_384_B=3.44	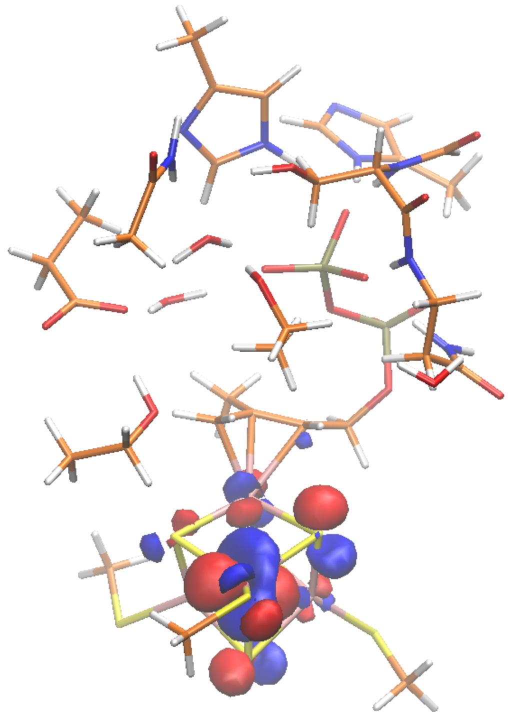<br>LUMO+2<br>MO_388_B=6.31
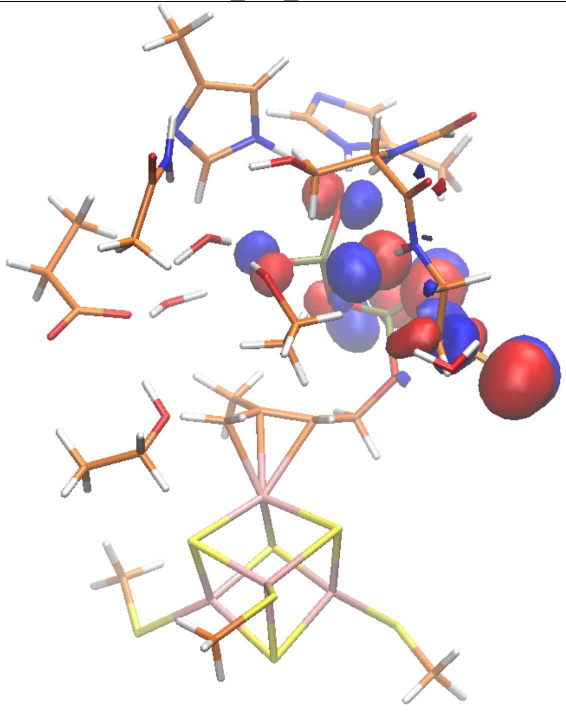<br>SOMO-3<br>MO_385_A=3.41	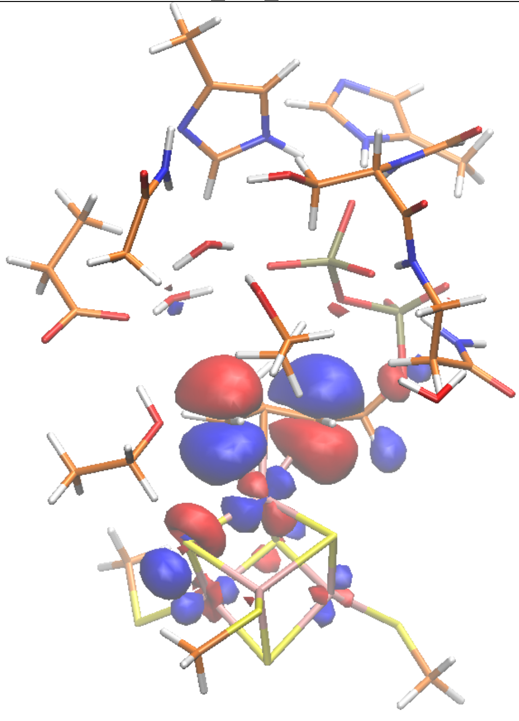<br>LUMO+3<br>MO_387_A=6.33

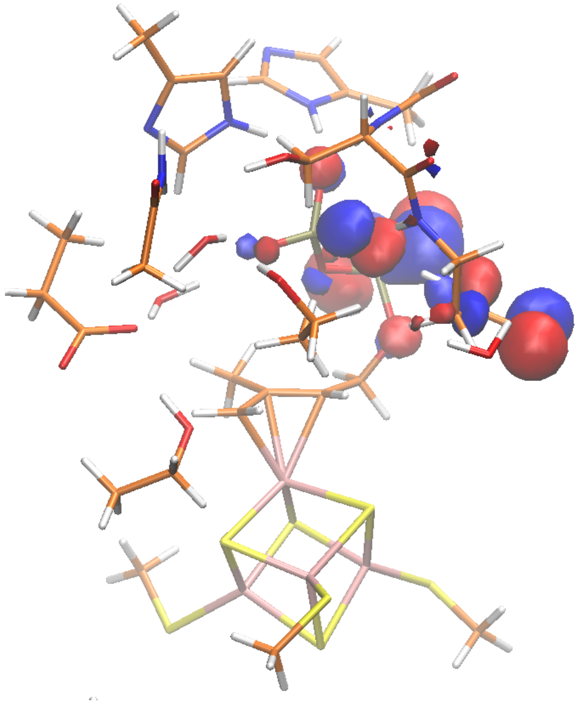

SOMO-4
MO_383_B=3.36

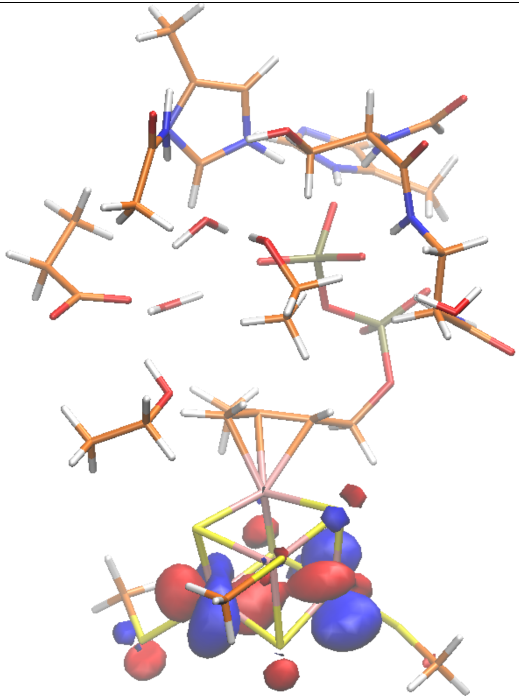

LUMO+3
MO_389_B=6.43

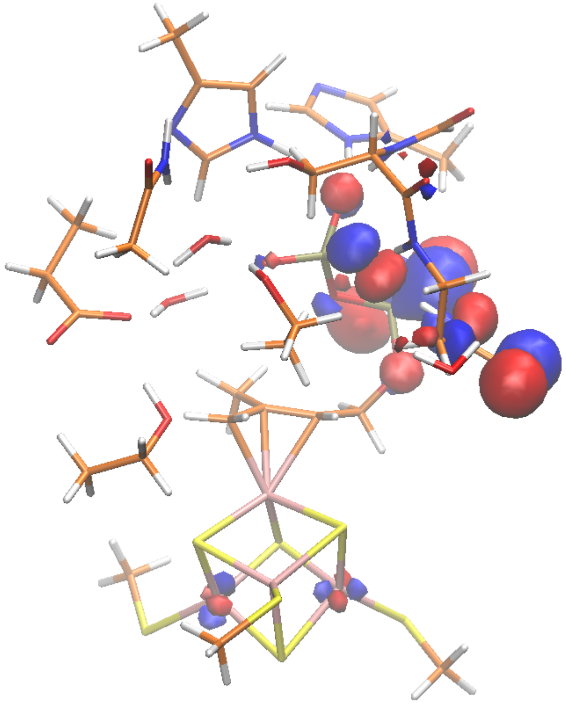

SOMO-5
MO_384_A=3.35

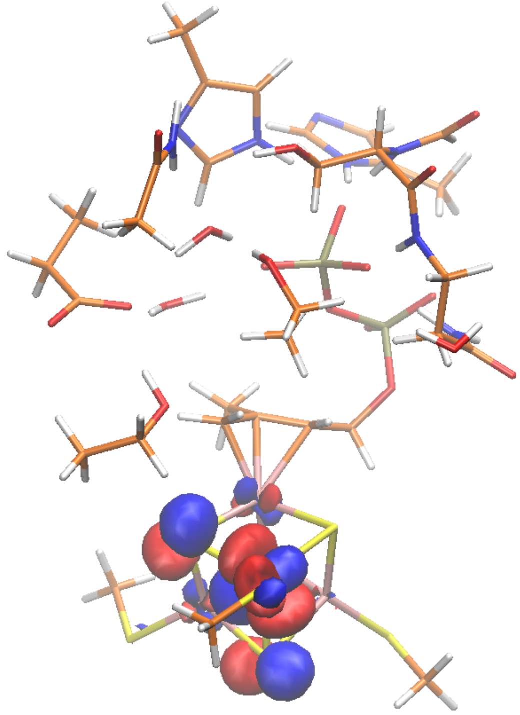

LUMO+5
MO_390_B=6.44

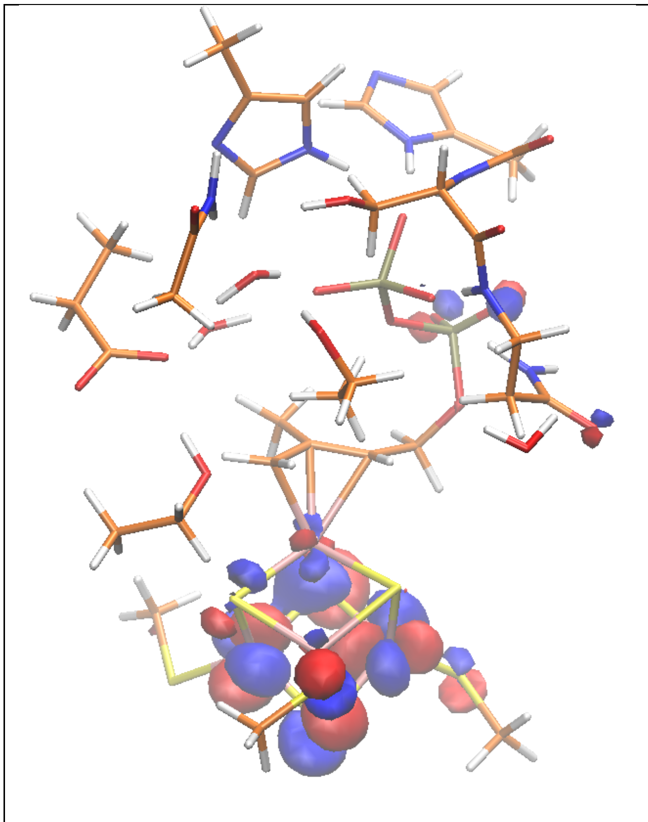

SOMO-6
MO_383_A=3.33

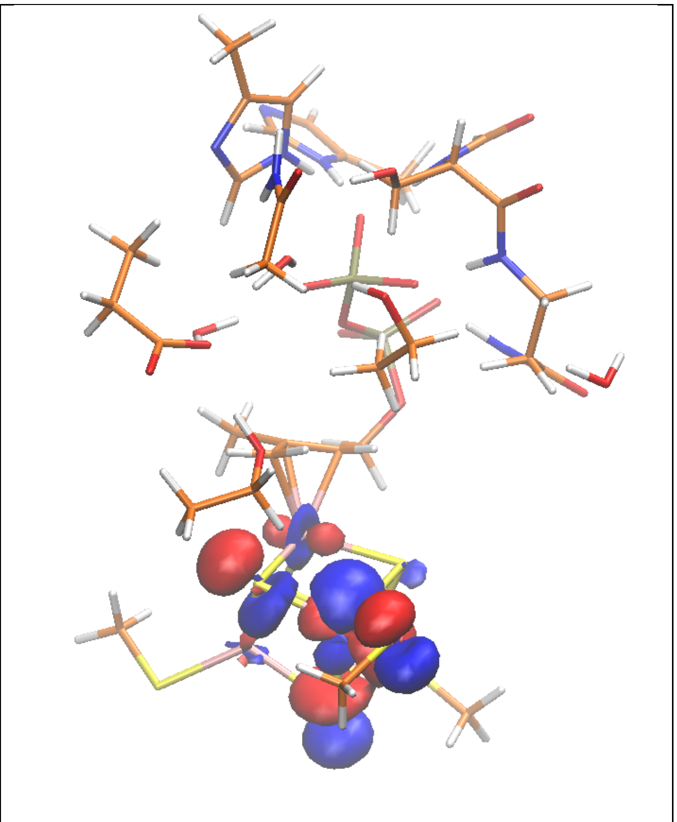

LUMO+6
MO_391_B=6.60

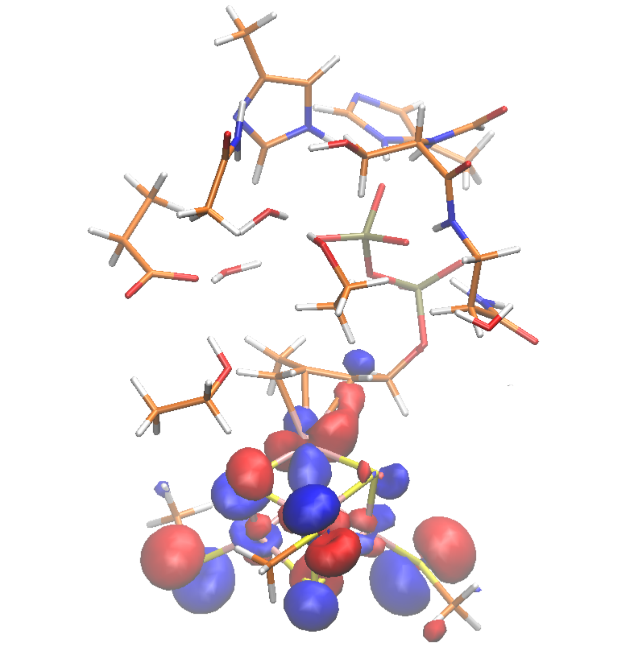

SOMO-7
MO_382_B=3.28

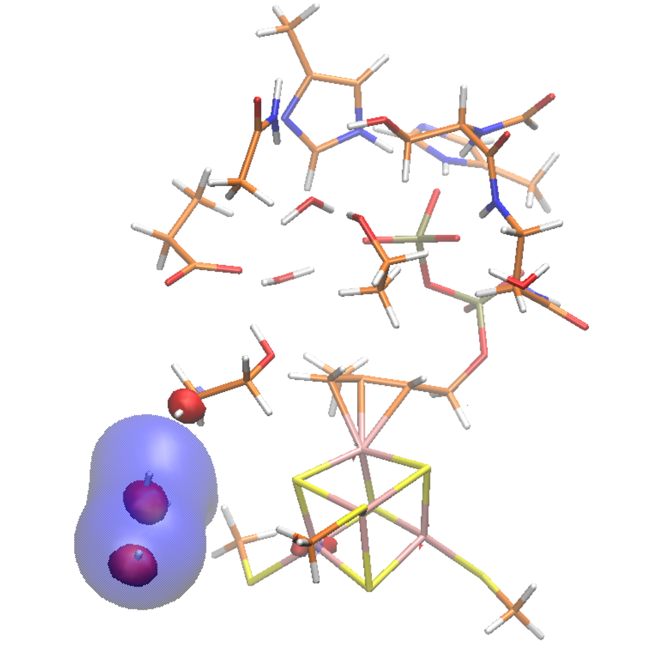

LUMO+7
MO_388_A=6.63

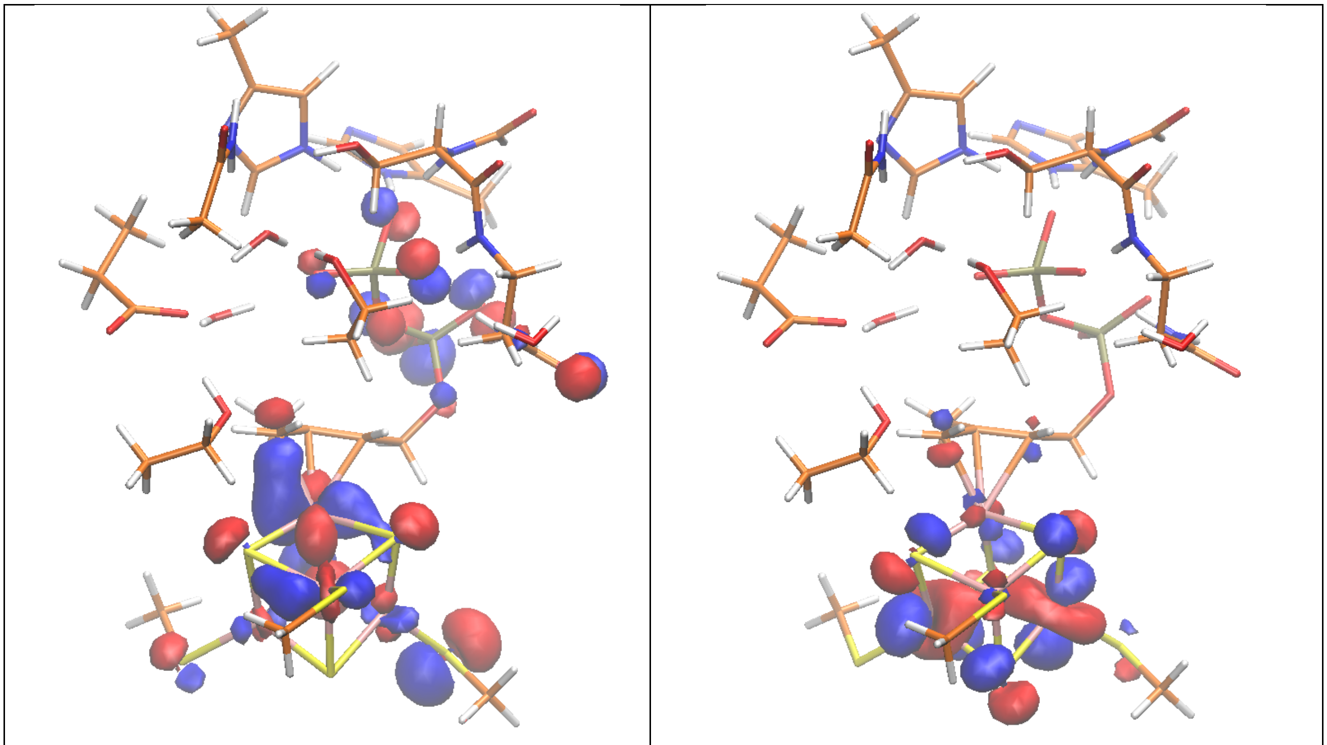

SOMO-8<br>MO_381_B=3.19	LUMO+8<br>MO_392_B=6.64

Table S10. Frontier molecular orbitals of I5 EH126 second step reaction step.

SOMO	LUMO
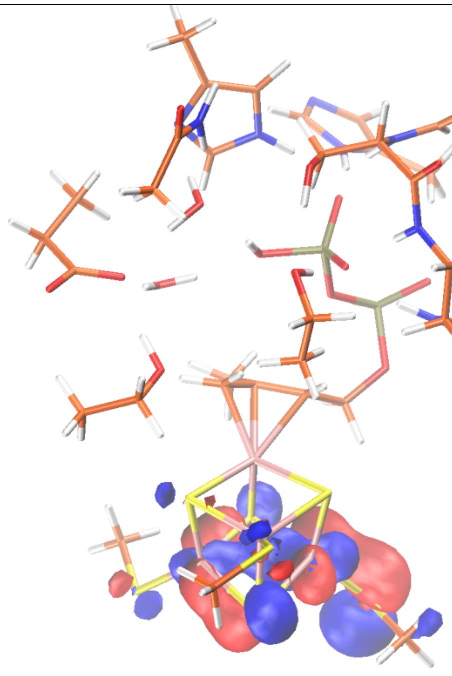<br>I3	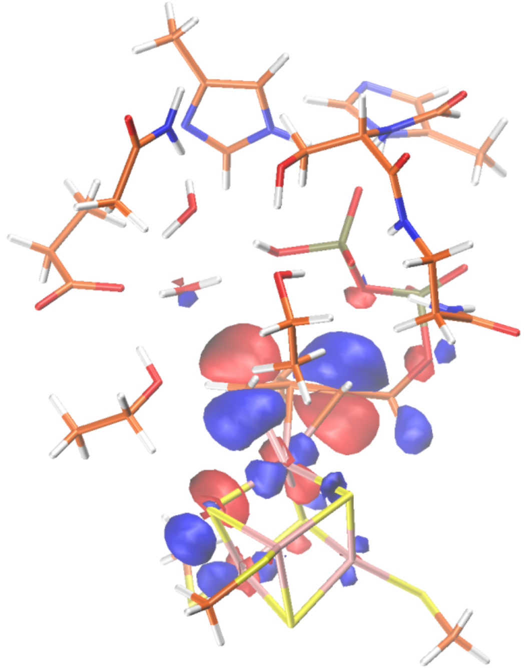<br>I3
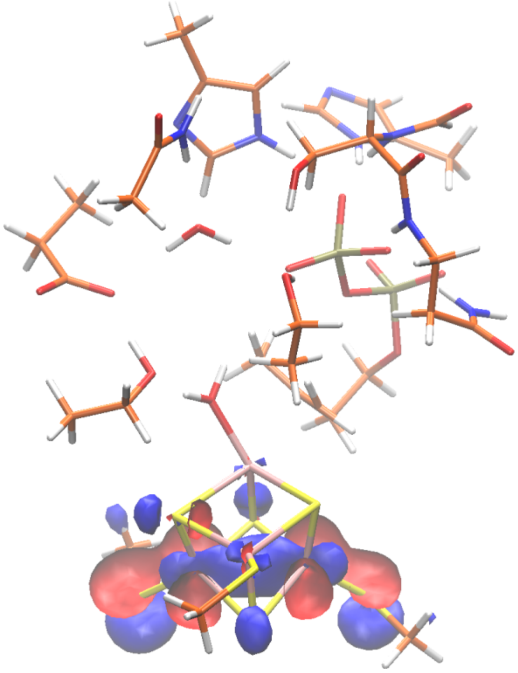<br>I5(C4-protonation)	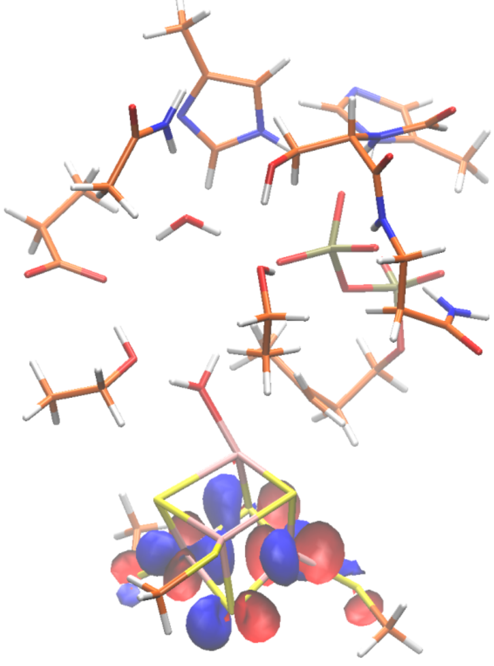<br>I5(C4-protonation)

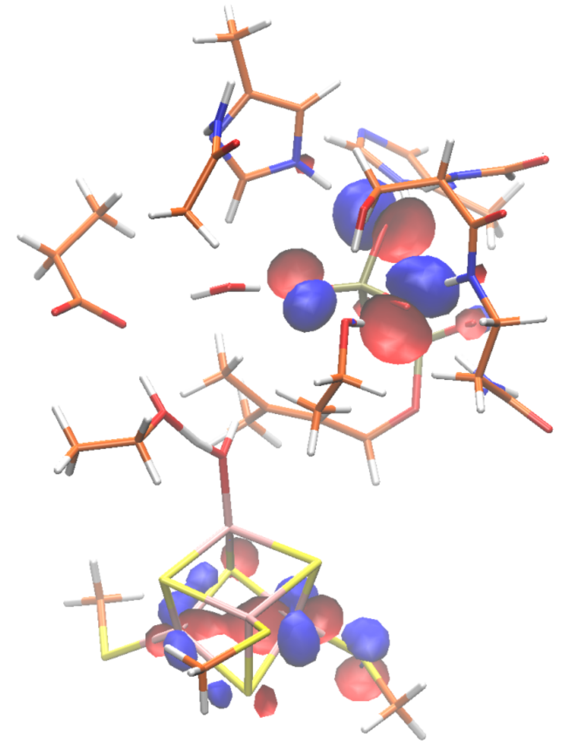

I5 (C2-protonation)

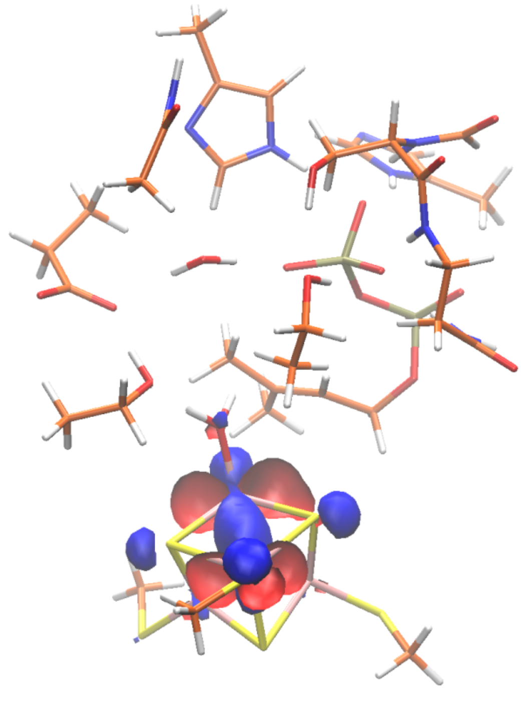

I5 (C2-protonation)

Table S11. NPA charges and spin densities of the stationary points of the e⁻/H+ coupled reaction calculated for the QM region and the protonated Glu126 using model M2 and optimized at ONIOM(B3LYP/TZVP:Amber) level of theory for the reduced state.

	R		TS3(C2-protonation)		TS3 (C4-protonation)		I5(C2-protonation)		I5 (C4-protonation)	
Atom	Charge	Spin	charge	Spin	Charge	Spin	Charge	Spin	Charge	Spin
4-O	-0.98301	-0.00201	-0.99042	0.00397	-0.9751	0.00036	-1.00874	0.05247	-1.00824	0.09815
4-H	0.48781	0.00009	0.49453	-0.00013	0.49181	-0.00001	0.52901	-0.00023	0.52702	0.00011
Hb3	0.51715	0.00002	0.42806	0.01144	0.41806	0.02028	0.21695	0.00067	0.19945	0.00091
WT1	0.02195	-0.0019	-0.06783	0.01528	-0.06523	0.02063	-0.26278	0.05291	-0.28177	0.09917
C4	-0.48104	-0.2577	-0.7155	0.25332	-0.68456	0.17608	-0.53525	-0.0352	-0.67577	-0.00008
H41	0.24204	0.00941	0.24958	-0.00057	0.24407	0.00021	0.21743	0.00281	0.24811	0.00003
H42	0.23579	0.00947	0.25792	0.00011	0.25544	-0.00132	0.2235	0.0007	0.23878	0.00102
C3	-0.07307	0.07469	0.20832	-0.05104	0.1776	-0.03451	0.14022	0.08799	-0.01363	0.00951
C2	-0.15767	-0.27528	-0.48938	0.15686	-0.47847	0.21515	-0.46744	-0.00447	-0.17597	0.01503
H21	0.22894	0.00791	0.23696	-0.00247	0.24694	-0.00024	0.20037	0.00275	0.20783	-0.0005
C1	-0.1257	0.01135	-0.06852	-0.00129	-0.09125	-0.0027	-0.05308	0.00468	-0.1103	-0.00062
H11	0.2086	-0.01088	0.18251	0.01255	0.19162	0.00439	0.15432	0.00014	0.1824	0.00129
H12	0.18208	-0.00158	0.19242	0.00277	0.1843	0.001	0.18943	-0.0002	0.1782	0.00021
C5	-0.65357	-0.00383	-0.67677	0.00296	-0.68248	0.00315	-0.6587	-0.00425	-0.6283	-0.0004
H51	0.22649	0.0038	0.22562	-0.00213	0.21963	-0.00093	0.21166	0.0049	0.19498	0.00087
H52	0.22827	0.00062	0.26086	-0.00201	0.21806	-0.00027	0.21845	0.00059	0.20565	0.00053
H53	0.24215	0.00216	0.21863	-0.00049	0.27267	-0.00218	0.23424	0.00309	0.20712	0.00004
Oa1	-0.77791	-0.00985	-0.78847	0.00011	-0.78662	0.01298	-0.79293	-0.00041	-0.78717	0.00047
Pa	2.44893	0.00011	2.44149	0.00023	2.4653	-0.00065	2.45022	-0.00498	2.45374	0.00159
Oab	-1.09784	-0.00454	-1.11954	0.0003	-1.12715	0.00498	-1.0852	0.01483	-1.10936	-0.00047
Pb	2.55123	-0.00006	2.56206	-0.00245	2.57315	-0.00378	2.51591	-0.03472	2.53464	0.02121
Ob3	-1.02847	-0.00019	-1.04458	0.08141	-1.07869	0.0887	-1.16051	0.03896	-1.16861	-0.04978
Ob2	-1.1884	-0.00046	-1.23119	0.00865	-1.21553	0.01777	-1.063	0.25201	-1.16606	-0.12398
Ob1	-1.1831	-0.00002	-1.24244	0.00375	-1.24235	0.00422	-0.88948	0.44734	-1.12176	-0.19132
Oa2	-1.12605	-0.00017	-1.12869	0.00022	-1.12634	0.00067	-1.12699	0.02526	-1.13394	-0.00783
Oa3	-1.14227	-0.00075	-1.13488	0.00021	-1.12877	0.00233	-1.1083	0.0285	-1.13255	-0.00965
4-O	-0.98301	-0.00201	-0.99042	0.00397	-0.9751	0.00036	-1.00874	0.05247	-1.00824	0.09815
HMBPP	-2.24057	-0.44579	-2.60359	0.461	-2.59343	0.48505	-2.18513	0.83032	-2.57197	-0.33283
Fe1	1.01369	-3.32377	1.03925	-3.47607	1.01827	-3.45559	1.06592	-3.52403	1.05777	-3.52575
Fe2	1.02927	3.12636	1.2061	3.64915	1.21362	3.62535	1.25921	3.57549	1.34701	3.76347
Fe3	1.13563	3.62911	1.17074	3.70358	1.159	3.68364	1.13798	3.63786	1.11321	3.64436
Fe4	0.92255	-2.93277	1.07348	-3.53164	1.08662	-3.55658	1.11791	-3.57482	1.01566	-3.34508
S1	-0.99122	0.2486	-0.92452	0.28809	-0.91533	0.30146	-1.00296	0.19746	-0.85466	0.31616
S2	-0.84441	0.26201	-0.90846	0.25566	-0.88377	0.20079	-1.04387	0.16329	-0.97934	0.42958
S3	-0.82783	0.18581	-0.86088	-0.12293	-0.8465	-0.07614	-0.95513	-0.18691	-0.86074	0.09231
S4	-0.92661	0.11999	-0.91867	-0.07511	-0.90969	-0.07878	-0.92585	-0.0891	-0.94081	-0.0377
Fe4S4(SCH3)3	-1.09311	1.44621	-0.60657	0.52088	-0.59147	0.49066	-0.97107	0.05597	-0.56535	1.23778
protein	-1.17158	0.00135	-1.20728	0.00302	-1.21758	0.00361	-1.08385	0.06055	-1.10641	-0.00476

Table S12. Reported the electron density (ρ(r)) in a.u. and its laplacian ($\nabla^2\rho(r)$) at CPs located at the interatomic distance of selected atoms. ρ(r) and $\nabla^2\rho(r)$ are reported as the sum of the alpha and beta contributions. Results are reported for the **R**, **INT1** and **INT2** minima.

Bond	ρ(r)	$\nabla^2\rho(r)$
	**R**	
Fe1-4O	0.062	0.305
Fe1-S1	0.068	0.142
Fe1-S2	0.085	0.160
Fe1-S4	0.056	0.134
C4-O	0.244	-0.479
C4-C3	0.260	-0.682
C2-C3	0.348	-1.017
	**INT1**	
Fe1-C2	0.027	0.037
Fe1-C3	0.060	0.131
Fe1-S1	0.049	0.110
Fe1-S2	0.084	0.160
Fe1-S4	0.062	0.150
C4-O	0.250	-0.487
C4-C3	0.283	-0.643
C2-C3	0.326	-0.911
	**INT2**	
Fe1-C2	0.023	0.030
Fe1-C3	NA	NA
Fe1-C4	0.067	0.112
Fe1-S1	0.062	0.110
Fe1-S2	0.089	0.163
Fe1-S4	0.068	0.110
C4-O	NA	NA
C4-C3	0.312	-0.885
C2-C3	0.313	-0.888

Table S13. ONIOM optimized parameters of IspH calculated at B3LYP/TZVP: AMBER level using the reduced state $[Fe_4S_4]^+$ and in parenthesis the optimized parameters using DFT+U and plane wave model of theory.

Cluster distance	R	TS1	I1	TS1	I2
Fe1-O	2.09(2.12)	2.79 (2.79)	3.58 (3.63)	4.38 (4.38)	5.28 (5.27)
Fe1-S1	2.25(2.22)	2.34 (2.22)	2.52 (2.43)	2.44 (2.36)	2.42 (2.35)
Fe1-S2	2.36(2.35)	2.24 (2.34)	2.25 (2.21)	2.23 (2.19)	2.23 (2.22)
Fe1-S4	2.39(2.29)	2.40 (2.28)	2.41 (2.37)	2.46 (2.42)	2.38 (2.31)
Fe2-S1	2.40(2.37)	2.37 (2.37)	2.35 (2.25)	2.34 (2.27)	2.34 (2.26)
Fe2-S2	2.37(2.35)	2.44 (2.36)	2.47 (2.42)	2.47 (2.37)	2.43 (2.36)
Fe2-S3	2.46(2.39)	2.43 (2.40)	2.40 (2.33)	2.39 (2.31)	2.40 (2.33)
Fe3-S1	2.48(2.40)	2.36 (2.40)	2.32 (2.27)	2.34 (2.30)	3.35 (2.30)
Fe3-S3	2.40(2.40)	2.31 (2.40)	2.29 (2.26)	2.29 (2.26)	2.28 (2.25)
Fe3-S4	2.39(2.27)	2.38 (2.28)	2.35 (2.31)	2.34 (2.32)	2.35 (2.32)
Fe4-S2	2.33(2.32)	2.44 (2.33)	2.40 (2.34)	2.42 (2.36)	2.42 (2.35)
Fe4-S3	2.32(2.30)	2.44 (2.30)	2.48 (2.40)	2.48 (2.40)	2.45 (2.38)
Fe4-S4	2.35(2.32)	2.43 (2.31)	2.40 (2.32)	2.38 (2.28)	2.38 (2.31)
Fe2-SC12	2.26(2.32)	2.26 (2.32)	2.26 (2.26)	2.24 (2.22)	2.24 (2.25)
Fe3-Sc197	2.30(2.27)	2.32 (2.26)	2.32 (2.29)	2.31 (2.28)	2.30 (2.27)
Fe4-Sc96	2.35(2.27)	2.31 (2.27)	2.32 (2.33)	2.31 (2.35)	2.29 (2.30)
Fe1-Fe2	2.84(2.76)	2.82 (2.81)	2.95 (2.88)	2.90 (2.83)	2.94 (2.87)
Fe1-Fe3	3.06(2.58)	2.63 (2.77)	3.02 (2.97)	3.06 (3.02)	3.06 (2.99)
Fe1-Fe4	2.92(2.79)	2.86 (2.60)	2.89 (2.85)	2.94 (2.91)	2.96 (2.92)
Fe2-Fe3	2.81(2.87)	2.83 (3.10)	2.78 (2.71)	2.80 (2.74)	2.80 (2.72)
Fe2-Fe4	2.74(2.78)	3.19 (2.89)	3.11 (3.01)	3.08 (2.97)	3.08 (2.96)
Fe3-Fe4	2.89(3.08)	2.95 (2.77)	3.00 (2.90)	2.98 (2.90)	2.97 (2.89)

Appendix: Gas phase and water solution calculations.

In the gas phase calculations, the model depicted in figure 2 was used to study the 4-OH group rotation and elimination. We have removed the diphosphate group as we loose the hairpin structure of the substrate upon optimization in vacuum. This shows the importance of the enzyme environment to keep the diphosphate in hairpin fold which is necessary for the catalysis. The idea behind the active site calculations is to check the electronic structures of some key intermediates expected to be involved in the reaction mechanism. So the active site calculations are not to conclude on the IspH catalysis rather than to have a general idea about these intermediates. First, we tried to optimize the protonated initial state (the alkoxide state) in which we added one proton to the 4-OH group coordinated to the apical iron atom of the cluster. Upon optimization the proton was migrated to one of the sulfur atoms bound to the cluster and the dehydration of the substrate did not occur.

Using the oxidized state $[Fe_4S_4]^{+2}$, the rotation of the 4-OH group is easy to happen with small barrier of only 4.3 kcal/mol in vacuum and 4.9 kcal/mol in solution. Overall solvent effect has no considerable effect on the energetics of the reaction profile except for the rotation of 4-OH group which decreases the stability of **I2**. For the dehydroxylation we tried to test the two proposed mechanism pathways; the organometallic and the Birch-like pathway. First, we simulated the organometallic pathway by the dissociation of the C-O bond and the formation of η^3-complex between the allylic moiety and the apical iron atom of the cluster. The dehydroxylation step is rather difficult with considerable high barrier of 63.8 kcal/mol and the reaction is endothermic (37.2 kcal/mol). Second, we simulated Birch-like by the dissociation of the hydroxyl group which then is bound to the cluster and formation of the free radical on the substrate. This step has lower barrier than the organometallic pathway and the reaction is less endothermic.

Concerning the electronic structures depicted in scheme 2, Table S16 reported the NPA charges and spin densities of the reaction profile stationary points. There are no any significant spin densities localized on the substrate from **R** to **I2**. Limited spin densities are located on C2 (-0.2e) and C4 (-0.2e). **I2** and **I3** are η^3-complexes and are agreeing well with the organometallic pathway though which reaction is kinetically unfavorable. This finding evidences the importance of the protein environment on the dehydroxylation step and its role in the stabilization of the reaction intermediates and transition state. **I2'** has significant spin density (-1.0e) transferred from the cluster located mainly on C2 (0.2e), C3 (-0.6e) and C4 (-0.6e). **I2'** is the free radical via which the Birch-like reduction of the substrate occurs using the cluster.

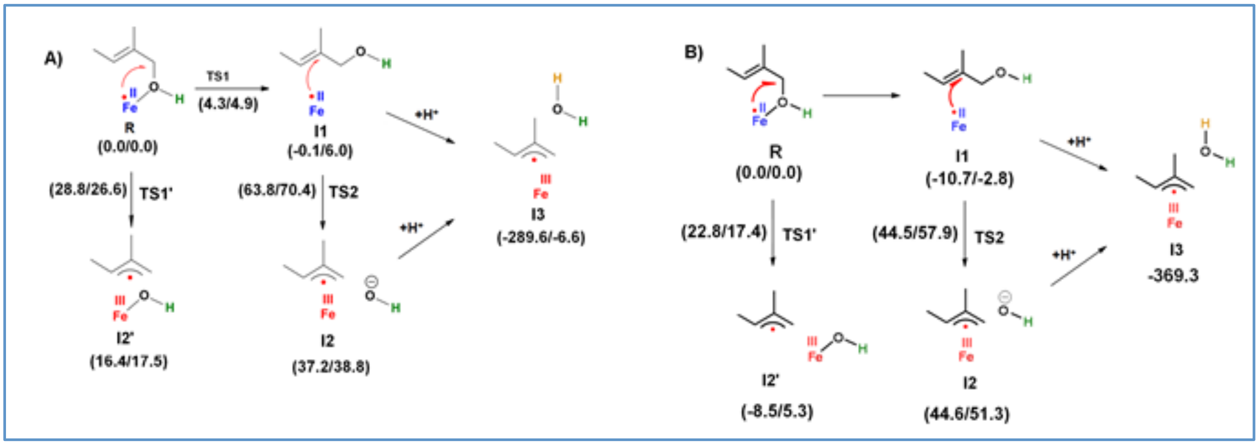

Scheme S1. reaction profile of the IspH catalysis using active site model depicted in figure 2. In A) the reaction profile used the oxidized state $[Fe_4S_4]^{+2}$, the first value in parenthesis is ΔE and second value is ΔE in solution of water. In B) is the reaction profile using the reduced state $[Fe_4S_4]^{+}$. All reported calculations are carried out using B3LYP/TZVP level of theory.

Using the reduced state $[Fe_4S_4]^{+}$, the situation does not change a lot the same relative tendency between the organometallic and Birch-like pathways is again applied. Also, limited spin densities are localized on the substrate in **INT1**, **INT2**, and **INT3** complexes (see Table S5). In the same way the free radical formation on **I2'** is evidenced by the important spin densities localized on C2 and C4 of the substrate (see Table S17).

Further, we considered state $[Fe_4S_4]^0$ in which all the iron atoms have Fe (II) oxidation state. On optimization of **R** using this state, does not provoke any rotation or elimination of the 4-OH group. So, the hypothesis stated that the electron transfer triggers the rotation or dehydroxylation of 4-OH group is ruled out. On conclusion of the simple model calculations; using the oxidized and the reduced states of the cluster, we could not observe any evidence of rupture of C-O bond initiated by the electronic transfer to the substrate. The reaction is easier on the surface of the reduced state than on the oxidized state, notably the rotation of the 4-OH group which is much more favorable using the reduced state.

Table S14: DFT/TZVP optimized parameters of the IspH active site model depicted in figure 2 are reported for R and I1. Values in parenthesis are the optimized parameters for the oxidized state.

Bond	R	I1
Fe1-O	2.29 (2.16)	3.62 (3.73)
Fe1-C2	3.88 (4.25)	2.22 (2.22)
Fe1-C3	3.52 (3.71)	2.26 (2.34)
Fe1-S1	2.34 (2.36)	2.42 (2.38)
Fe1-S2	2.35 (2.43)	2.27 (2.47)
Fe1-S4	2.36 (2.36)	2.42 (2.28)
Fe2-S1	2.47 (2.34)	2.38 (2.35)
Fe2-S2	2.48 (2.37)	2.50 (2.37)
Fe2-S3	2.46 (2.30)	2.47 (2.28)
Fe2-Sc_{197}	2.36 (2.29)	2.33 (2.27)
Fe3-S1	2.38 (2.38)	2.39 (2.38)
Fe3-S3	2.26 (2.35)	2.26 (2.39)
Fe3-S4	2.36 (2.35)	2.36 (2.39)
Fe3-Sc_{96}	2.36 (2.28)	2.33 (2.39)
Fe4-S2	2.49 (2.36)	2.42 (2.34)
Fe4-S3	2.48 (2.41)	2.46 (2.36)
Fe4-S4	2.45 (2.43)	2.47 (2.36)
Fe4-Sc_{12}	2.36 (2.29)	2.33 (2.28)

Table S15. NPA charges and spin densities calculated for the active site model depicted in figure 2 in main text using the oxidized state. For INT3 the charge and the spin of the added proton summed to the oxygen atom of the 4-OH group.

Atom	R		TS1		I1		TS2		I2	
O	-0.75821	0.03118	-0.73125	0.01674	-0.71018	0.00284	-0.67747	-0.74276	-0.83626	0.01569
H	0.50909	0.00023	0.50349	0.00028	0.48309	-0.00004	0.42703	0.01721	0.49795	0.00036
C	-0.08331	0.00346	-0.11033	-0.00084	-0.09023	0.0008	-0.33815	0.14366	-0.48413	-0.18305
H	0.16887	0.0012	0.18894	0.00091	0.17247	-0.00336	0.23853	-0.00642	0.21234	0.00628
H	0.18824	-0.00018	0.17676	0.00151	0.17196	-0.00015	0.19172	-0.00476	0.21649	0.00407
C	-0.04429	0.00064	-0.0249	0.02095	-0.07655	-0.02692	-0.02776	-0.10295	-0.08204	0.02868
C	-0.6443	-0.00058	-0.63917	-0.00083	-0.64286	0.00135	-0.64601	0.00507	-0.62126	-0.00087
H	0.2151	-0.00003	0.21573	0.00002	0.21711	-0.00018	0.21418	-0.00346	0.20537	-0.00074
H	0.24316	-0.0004	0.23561	0.0009	0.2408	-0.0002	0.24781	-0.00028	0.23791	0.00021
H	0.19842	0	0.20654	0.00064	0.20616	-0.00095	0.20254	-0.00452	0.21132	-0.00012
C	-0.13503	0.00057	-0.23847	0.00124	-0.32036	0.00256	-0.47706	0.17822	-0.25572	-0.19272

H	0.18569	-0.00001	0.23072	0.00058	0.22423	0.00219	0.21986	-0.00091	0.21719	0.00526
C	-0.63384	0	-0.63251	0.00056	-0.62751	0.00092	-0.61871	-0.00358	-0.6351	0.00947
H	0.20327	-0.00001	0.20711	0.00016	0.20475	0.0002	0.19426	0.01113	0.1972	-0.01275
H	0.23365	0.00006	0.23539	0.00003	0.22608	0.00003	0.21859	0.00196	0.23379	-0.00262
H	0.20603	0	0.21067	-0.00002	0.21796	-0.00011	0.20951	0.00146	0.22481	-0.0022
SUM	0.05254	0.03613	0.03433	0.04283	-0.10308	-0.02102	-0.42113	-0.51093	-0.46014	0.05254
S12	-0.45415	-0.19943	-0.45799	-0.19524	-0.4109	-0.25314	-0.40385	-0.2386	0.47984	-0.03095
C	-0.70107	-0.0038	-0.70076	-0.00383	-0.70302	-0.004	-0.70279	-0.00425	-0.7462	-0.00504
H	0.20237	-0.00387	0.20236	-0.00384	0.20623	-0.00458	0.20551	-0.00455	0.22817	-0.00107
H	0.19165	-0.00409	0.19202	-0.00389	0.19517	-0.00501	0.19739	-0.00454	0.21113	-0.00162
H	0.20254	-0.00383	0.20187	-0.00385	0.20395	-0.00453	0.20375	-0.00449	0.2291	0.00037
S96	-0.40906	-0.25876	-0.40662	-0.26522	-0.44035	-0.20338	-0.40924	-0.23662	-0.4905	-0.13565
C	-0.70499	-0.00447	-0.70526	-0.0042	-0.70533	-0.00437	-0.70566	-0.00451	-0.7059	-0.00262
H	0.20225	-0.0044	0.20353	-0.00446	0.20004	-0.00359	0.20273	-0.00414	0.1936	-0.00274
H	0.19361	-0.00497	0.19439	-0.00521	0.19377	-0.00484	0.19724	-0.00506	0.18916	-0.00478
H	0.20867	-0.00456	0.20821	-0.00462	0.20364	-0.00409	0.2049	-0.00448	0.19933	-0.00337
S197	-0.40133	0.28555	-0.39018	0.29542	-0.37065	0.31743	-0.35899	0.33061	-0.40211	0.29652
C	-0.70552	0.00337	-0.7052	0.00381	-0.70398	0.00376	-0.70085	0.00459	-0.70517	0.00267
H	0.20829	0.00471	0.207	0.00487	0.20717	0.00555	0.20813	0.00629	0.20105	0.00521
H	0.19423	0.00565	0.19513	0.00572	0.1974	0.00527	0.19937	0.00448	0.19334	0.00509
H	0.20684	0.00463	0.20824	0.0049	0.21121	0.0054	0.21156	0.00577	0.20972	0.00483
Fe1	1.09482	3.47548	1.09398	3.46414	1.11654	3.43137	1.20824	3.63147	1.00837	3.01261
Fe2	1.0965	-3.55748	1.09872	-3.55305	1.10714	-3.5868	1.06891	-3.53431	0.95579	-3.36302
Fe3	1.10957	-3.60037	1.1159	-3.60624	1.08375	-3.53842	1.08099	-3.54985	1.10293	-3.52867
Fe4	1.13098	3.64136	1.14552	3.64407	1.17965	3.68083	1.17707	3.68579	1.20799	3.71275
S1	-0.96182	-0.12972	-0.96684	-0.11527	-0.95068	-0.12923	-0.8901	-0.0548	-0.98872	-0.13821
S2	-1.06289	0.2009	-1.08032	0.18812	-1.02367	0.0962	-0.95062	0.22302	-1.12761	0.27285
S3	-0.97777	0.19218	-0.97295	0.18528	-0.99564	0.24344	-0.93779	0.26363	-0.97967	0.17458
S4	-0.91624	-0.07022	-0.91507	-0.07023	-0.89839	-0.02226	-0.88479	0.00547	-1.00354	0.05533
SUM	-1.05252	-0.03614	-1.03432	-0.04282	-0.89695	0.02101	-0.57889	0.51092	-0.5399	0.32507

Atom	TS1'		I2'		I3	
O	-1.01889	0.19817	-1.06375	0.28749	-0.49427	-0.00085
H	0.45804	0.00133	0.46155	0.00318	0.47971	-0.00062
C	-0.12854	-0.28614	-0.36271	-0.59533	-0.47106	-0.21034
H	0.18881	0.0095	0.17091	0.01712	0.26185	0.00769
H	0.20671	0.00842	0.20009	0.01585	0.21986	0.00711
C	-0.14321	0.09395	-0.0968	0.17696	-0.05603	0.03771
C	-0.62392	-0.00528	-0.62176	-0.01064	-0.64311	-0.00146
H	0.20666	0.00243	0.20545	0.00648	0.22453	0.00027
H	0.22492	0.00393	0.20733	0.00669	0.22586	0.00068

H	0.20425	0.00057	0.20336	0.00093	0.25704	-0.00015
C	-0.01891	-0.27859	-0.11236	-0.60298	-0.20319	-0.20794
H	0.2053	0.00835	0.21096	0.01696	0.22092	0.00584
C	-0.6721	0.01602	-0.65056	0.03067	-0.64295	0.00882
H	0.21853	-0.01273	0.2136	-0.0369	0.22528	-0.01491
H	0.27488	-0.01664	0.2314	-0.02314	0.23595	-0.00279
H	0.20184	-0.00055	0.196	-0.00205	0.22526	-0.00237
SUM	-0.21563	-0.25726	-0.60729	-0.70871	0.06565	-0.00085
S12	-0.43004	-0.2228	-0.41518	-0.22292	-0.24395	0.15367
C	-0.70237	-0.00489	-0.70226	-0.00429	-0.70936	-0.00822
H	0.20523	-0.00408	0.20526	-0.0043	0.22182	0.00376
H	0.19336	-0.00493	0.1964	-0.00426	0.21751	0.00599
H	0.20316	-0.00403	0.20355	-0.00432	0.21613	-0.00295
S96	-0.42886	-0.21591	-0.39412	-0.25211	-0.28568	-0.37263
C	-0.70395	-0.00588	-0.70604	-0.00468	-0.70422	-0.00655
H	0.20076	-0.00384	0.20343	-0.00438	0.214	-0.0065
H	0.19285	-0.00418	0.19772	-0.00505	0.21593	-0.00634
H	0.20601	-0.00407	0.20594	-0.00466	0.21458	-0.00678
S197	-0.40856	0.27875	-0.36061	0.32855	-0.29136	0.39309
C	-0.70367	0.00352	-0.70105	0.00459	-0.7042	0.00462
H	0.20725	0.00403	0.20772	0.00622	0.21345	0.0075
H	0.19565	0.00559	0.19911	0.00451	0.21547	0.00634
H	0.21023	0.00495	0.2112	0.00578	0.2148	0.00679
Fe1	1.26015	3.65048	1.35632	3.76691	1.01467	3.08762
Fe2	1.09194	-3.56238	1.05864	-3.52411	0.93419	-3.02466
Fe3	1.09268	-3.55112	1.07662	-3.55801	1.1148	-3.60404
Fe4	1.13227	3.64475	1.16795	3.68399	1.14666	3.65358
S1	-0.94366	-0.11583	-0.86462	-0.04342	-0.74067	-0.10928
S2	-0.96676	0.18903	-0.92443	0.27131	-0.86086	0.14666
S3	-0.97571	0.23947	-0.93086	0.26947	-0.86394	0.05995
S4	-0.91231	-0.05938	-0.88341	0.0039	-0.81541	-0.00833
SUM	-0.78435	0.25725	-0.39272	0.70872	-0.06564	0.37329

Table S16: NPA charges and spin densities calculated for the active site model depicted in figure 2 in main text using the oxidized and the reduced states of the cluster. For INT3 the charge and the spin of the added proton summed to the oxygen atom of the 4-OH group.

Atom	R		I1		TS2		I2	
O	-0.77249	0.01289	-0.73605	0.00401	-0.71795	0.7137	-0.87652	0.54065
C	-0.08573	0.00218	-0.07999	0.00074	-0.41843	0.14141	-0.51644	-0.19529
H	0.15095	0.00103	0.15163	-0.00228	0.21869	-0.00019	0.24	0.00812
H	0.17397	-0.00004	0.16552	-0.00033	0.18356	-0.00433	0.20596	0.00546
H	0.50887	-0.00106	0.49015	0.00001	0.43048	-0.01678	0.43098	-0.01339
C	-0.03505	0.00141	-0.13957	-0.03536	0.02901	0.04803	-0.07962	0.04537
C	-0.65249	-0.00083	-0.63489	0.0018	-0.63886	-0.00145	-0.63949	-0.00116
H	0.2081	-0.00012	0.17969	-0.00534	0.22161	0.00297	0.20593	0.00008
H	0.26522	0.00015	0.22425	-0.0001	0.21373	-0.0001	0.28534	0.00044
H	0.18685	-0.00006	0.23106	-0.00015	0.18823	0.00183	0.18538	0.00098
C	-0.15101	-0.0002	-0.31636	-0.04726	-0.60585	0.18988	-0.282	-0.15798
H	0.17929	0.00002	0.21712	0.00206	0.20429	0.00289	0.20804	0.00539
C	-0.63782	0.00076	-0.62625	0.003	-0.60968	-0.00348	-0.62673	0.00956
H	0.18603	0.0001	0.1796	-0.00518	0.16102	0.01607	0.18025	-0.01111
H	0.24973	-0.00014	0.22497	-0.00008	0.20618	0.00079	0.22861	-0.00296
H	0.20343	-0.00004	0.2212	-0.00075	0.1986	0.00199	0.21283	-0.0013
SUM	-0.02215	0.01605	-0.24792	-0.08521	-0.73537	1.09323	0.23904	0.23286
S	-0.55203	-0.09745	-0.53858	-0.11076	-0.46719	-0.20019	-0.54527	-0.1106
C	-0.7088	0.00433	-0.70566	0.00108	-0.70769	-0.00412	-0.70771	0.00247
H	0.20039	-0.00245	0.19326	-0.00271	0.20078	-0.00331	0.20912	-0.00281
H	0.16875	-0.00333	0.17171	-0.00288	0.1789	-0.00416	0.17033	-0.0025
H	0.18337	-0.00249	0.19717	-0.00262	0.20009	-0.00388	0.19242	-0.00257
S	-0.48308	0.21723	-0.46782	0.23434	-0.48785	0.19168	-0.46635	0.23189
C	-0.70634	0.00194	-0.70452	0.00221	-0.70323	0.00473	-0.70499	0.00272
H	0.19599	0.00362	0.19402	0.00395	0.20093	0.00357	0.19285	0.00383
H	0.17378	0.00371	0.17564	0.0033	0.17832	0.0038	0.17616	0.00407
H	0.20773	0.00357	0.21083	0.00401	0.20213	0.0033	0.21107	0.0038
S	-0.55649	-0.09872	-0.54587	-0.10587	-0.49121	-0.17999	-0.51956	-0.09432
C	-0.70702	0.00321	-0.70365	-0.00031	-0.70558	-0.00264	-0.70446	-0.00004
H	0.19675	-0.00229	0.19826	-0.00257	0.19689	-0.00342	0.1993	-0.00212
H	0.16953	-0.00277	0.17137	-0.00272	0.17744	-0.00371	0.17304	-0.00284
H	0.18702	-0.00266	0.1938	-0.00257	0.2017	-0.00341	0.19667	-0.00252
Fe1	1.10249	3.51367	1.12503	3.43311	1.17141	3.62439	1.01693	3.00405
Fe2	1.14124	-3.55223	1.12151	-3.49769	1.10327	-3.57762	1.11181	-3.48651
Fe3	1.20336	3.70717	1.2285	3.72973	1.12357	3.60737	1.18496	3.68559
Fe4	1.14157	-3.54877	1.11572	-3.51228	1.09641	-3.567	1.07029	-3.42232
S1	-1.09269	0.3095	-1.13003	0.3001	-0.98862	0.19453	-0.96158	0.22472
S2	-1.23556	0.03739	-1.11382	0.12201	-0.98086	-0.16726	-0.81547	0.37837
S3	-1.1222	0.30583	-1.08363	0.27127	-0.99027	0.17641	-0.99493	0.18198
S4	-1.08563	0.18592	-1.05533	0.2231	-0.97395	-0.18227	-1.04716	0.1728
SUM	-1.97787	0.98393	-1.75209	1.08523	-1.26461	-0.0932	-1.36253	0.76714

Atom	TS1'		I2'		I3	
O	-0.9496	0.11003	-1.14832	0.18301	-0.49437	0.00187
C	-0.17556	-0.31798	-0.39692	0.56465	-0.47857	-0.18442
H	0.16973	0.01039	0.17242	-0.01624	0.22958	0.00707
H	0.20141	0.00954	0.16476	-0.0153	0.21671	0.0049
H	0.45891	0.00099	0.44663	0.00305	0.48053	0.00009
C	-0.13462	0.09136	-0.06818	-0.16734	-0.06615	0.03996

C	-0.62402	-0.00522	-0.64769	0.00943	-0.63451	-0.0006
H	0.19634	0.00139	0.21217	-0.00729	0.21044	0.00056
H	0.21554	0.00358	0.2795	-0.00525	0.22889	0.0006
H	0.18961	0.00078	0.19082	-0.00099	0.2408	-0.00002
C	-0.11312	-0.27099	-0.10179	0.61948	-0.25969	-0.17397
H	0.20953	0.00727	0.15944	-0.01836	0.2171	0.00462
C	-0.65512	0.0132	-0.6822	-0.02677	-0.63398	0.00946
H	0.19445	-0.01252	0.19469	0.00126	0.20141	-0.012
H	0.26898	-0.01342	0.28986	0.02455	0.23231	-0.00254
H	0.18585	-0.00046	0.21412	0.03754	0.22236	-0.00216
S	-0.53798	-0.10779	-0.47424	-0.1998	-0.47106	-0.10478
C	-0.70698	0.0021	-0.70714	-0.00328	-0.70286	0.00131
H	0.19229	-0.00282	0.19818	-0.00325	0.20611	-0.00298
H	0.17081	-0.00304	0.17705	-0.00434	0.19185	-0.00267
H	0.19516	-0.00249	0.20374	-0.00349	0.19891	-0.00159
SUM	-0.36169	-0.37206	-0.72069	1.18543	-0.55103	-0.30616
S	-0.47001	0.23121	-0.49189	0.17211	-0.38565	0.30783
C	-0.7052	0.00216	-0.70727	0.00325	-0.70565	0.00434
H	0.19367	0.004	0.20201	0.00325	0.2038	0.0053
H	0.17509	0.00354	0.17636	0.00423	0.19672	0.00613
H	0.21061	0.00393	0.19588	0.00288	0.20924	0.00484
S	-0.55297	-0.10028	-0.50704	-0.16003	-0.34206	0.00078
C	-0.70346	0.0007	-0.70384	-0.0026	-0.71128	-0.00519
H	0.20399	-0.00235	0.1996	-0.00307	0.20925	0.00324
H	0.1711	-0.00233	0.17547	-0.00357	0.19401	-0.00164
H	0.19091	-0.00258	0.19849	-0.00309	0.20835	-0.00173
Fe1	1.24938	3.64386	1.25803	3.64172	0.99354	3.00143
Fe2	1.10815	-3.4951	1.12057	-3.61241	1.02105	-3.29783
Fe3	1.2116	3.72112	1.11283	3.59026	1.16507	3.67395
Fe4	1.11217	-3.51485	1.10623	-3.57606	0.9909	-3.10875
S1	-1.06746	0.36787	-0.98414	0.14861	-0.88817	0.24457
S2	-1.12282	0.13395	-0.99962	-0.18474	-0.90438	0.20678
S3	-1.09143	0.29491	-1.02614	0.17108	-0.92781	0.14625
S4	-1.06491	0.19634	-1.00243	-0.16309	-0.86274	0.22696
SUM	-1.63829	1.37206	-1.27931	-0.18543	-0.91286	1.30655

ONIOM-optimized coordinates of the QM region (M2) of the reaction profile stationary points for the protonated glutamate.

R

H	-6.296118	-6.805729	0.487272
C	-5.908076	-5.778680	0.638839
H	-6.757160	-5.122364	0.834126
H	-5.286553	-5.780981	1.538523
S	-4.956435	-5.140613	-0.810642
H	6.058562	-5.394910	-3.389849
N	6.405882	-2.004431	-3.292768
H	5.935654	-1.719583	-2.431470
C	6.704169	-3.293319	-3.660870
C	6.446204	-4.517911	-2.826369
H	5.748526	-4.261181	-2.034212
H	7.389840	-4.789917	-2.357844
N	7.451141	-1.900022	-5.235406
C	7.355679	-3.243994	-4.863345
H	7.756605	-4.088138	-5.405196
C	6.866207	-1.191909	-4.257248
H	6.780481	-0.115669	-4.253959
H	-6.128712	0.201279	-6.256406
C	-5.797418	0.018749	-5.205211
H	-5.331113	-0.966395	-5.162372
H	-5.011755	0.742661	-4.987137
S	-7.086203	0.147149	-3.870165
H	8.306832	4.676920	-2.812886
N	6.395264	1.158480	-1.861274
H	6.369078	0.171661	-1.588495
C	7.300395	2.108424	-1.465657
H	8.227140	1.944517	-0.928188
N	5.665693	3.060963	-2.684014
C	6.852661	3.283272	-1.982036
C	5.450130	1.736142	-2.614888
H	4.611127	1.202625	-3.047397
C	7.622257	4.567344	-1.941925
H	8.203229	4.572783	-1.017312
H	6.929262	5.408794	-1.905131
H	3.253233	5.229951	-4.054279
C	2.923295	4.807164	-3.081175
H	2.849146	3.724910	-3.194614
H	3.693633	5.014863	-2.335865
C	1.576461	5.390729	-2.650403
H	1.627578	6.470471	-2.486012
H	0.829744	5.253604	-3.439718
C	0.928699	4.809382	-1.403140
O	-0.133278	5.283934	-0.991259
O	1.437328	3.790969	-0.775640
H	2.431917	3.492434	-0.889396
H	2.194088	6.530114	2.319480
C	2.564383	5.489743	2.192811
H	2.158522	4.857692	2.981650
H	2.238949	5.109463	1.228931
C	4.088736	5.445980	2.259826
O	4.714603	6.037597	3.118670
N	4.765231	4.781813	1.349590
H	5.755543	4.990024	1.362195
H	4.307160	4.242911	0.624121
H	-2.206204	4.163773	1.827011
C	-2.628408	3.683131	0.914624
H	-3.312107	2.892426	1.223750
O	-1.557971	3.033639	0.208897
H	-1.052581	3.719177	-0.256582
C	-3.344909	4.645773	-0.023415
H	-3.863915	4.079153	-0.796203
H	-4.091404	5.253319	0.498103
H	-2.641068	5.333186	-0.496696
H	0.680190	1.736026	3.646793
C	1.364687	1.228616	2.932354
H	1.964506	0.492142	3.460508
O	2.222680	2.160286	2.309282
H	2.561411	1.721494	1.519909
C	0.581842	0.537668	1.817836
H	1.252644	-0.046379	1.195420
H	-0.166227	-0.130540	2.245409
H	0.083808	1.275187	1.193874
H	-7.441853	1.883600	3.871069
C	-7.262972	1.252242	2.981214
H	-8.185053	0.720288	2.742073
H	-7.047508	1.909279	2.139629
S	-5.877478	0.058527	3.228075
H	7.248107	-2.876268	0.857100
C	7.167917	-2.183115	1.717562
O	7.632422	-2.466666	2.825869
N	6.519929	-1.044249	1.497395
H	6.151882	-0.936440	0.555309
C	6.228978	-0.027476	2.509241
H	7.176295	0.334913	2.908907
C	5.522678	1.149372	1.836683
H	6.086306	1.446430	0.952396
H	4.517236	0.859248	1.534753
O	5.457005	2.244700	2.719873
H	5.110188	3.005979	2.232250
C	5.412975	-0.585258	3.686419
O	5.809406	-0.432320	4.843185
N	4.279643	-1.244033	3.420788
H	4.019784	-1.359477	2.443954
C	3.403886	-1.821124	4.438168
H	3.168137	-1.001506	5.115614
C	2.060996	-2.302201	3.848052
H	1.323723	-2.317324	4.645548
H	1.707173	-1.610716	3.092057

C	2.065315	-3.686194	3.236842
O	1.712924	-4.666595	3.867887
N	2.452870	-3.797171	1.996706
H	2.544374	-4.702243	1.577465
H	2.742539	-2.962972	1.486285
H	3.921602	-2.586122	5.055344
O	3.695768	2.829952	-0.521434
H	4.413768	3.009190	-1.161293
H	3.476589	1.825778	-0.680345
O	1.377115	-1.109338	7.089081
H	1.647678	-0.330613	6.574607
H	2.132601	-1.716189	7.028832
O	-0.568244	-3.369364	6.284692
H	-0.070716	-2.585920	6.549128
H	-0.736203	-3.253079	5.332724
O	-0.244679	8.060795	-1.462875
H	-0.755044	8.448953	-0.728682
H	-0.388150	7.101259	-1.355524
O	-6.657113	4.271009	0.824593
H	-5.973315	4.589039	1.445105
H	-6.264472	3.452535	0.451144
O	-1.218987	-2.412212	3.664056
H	-1.701856	-1.634578	3.998689
H	-1.725596	-2.608504	2.858621
O	-9.056646	5.097077	2.255470
H	-8.706209	5.565255	3.038989
H	-8.253072	4.738557	1.829545
O	-7.699689	6.894413	-0.159466
H	-7.173910	7.664263	0.146450
H	-7.111106	6.132250	-0.008222
C	-0.916507	0.459731	-1.980600
O	-1.610238	0.542468	-0.727277
C	-0.482866	-0.950444	-2.284876
C	-0.349050	-1.877672	-1.339419
C	-0.053866	-3.331001	-1.585978
O	1.142784	-3.776878	-0.949663
P	2.612715	-3.579665	-1.692539
O	2.804870	-1.915895	-1.620232
P	3.712392	-1.111410	-0.543702
O	3.009031	0.446604	-0.929478
O	5.147355	-1.025529	-0.986979
O	3.319467	-1.408658	0.869741
O	2.517142	-3.841643	-3.175131
O	3.605451	-4.245133	-0.779015
C	-0.305821	-1.209054	-3.752399
H	-0.048808	1.134200	-1.939516
H	-1.574031	0.810019	-2.781611
H	-1.615076	1.470613	-0.392941
H	-0.507526	-1.606370	-0.301776
H	-0.868463	-3.912330	-1.142728
H	-0.028062	-3.562057	-2.653696
H	0.433423	-1.983459	-3.950244
H	-1.269968	-1.503533	-4.181592
H	0.004308	-0.298350	-4.277450
H	2.054317	0.331707	-1.041941
Fe	-4.838259	-2.916754	-0.406351
Fe	-3.450528	-0.451597	-0.641603
Fe	-5.497027	-0.557440	1.014933
Fe	-6.056551	-0.389925	-1.870980
S	-3.527630	-1.852064	1.247360
S	-5.118510	1.309427	-0.412329
S	-4.078538	-1.720067	-2.387339
S	-7.012222	-1.848741	-0.169161

TS1

H	-6.349661	-6.778336	0.523431
C	-5.954808	-5.753627	0.670982
H	-6.799736	-5.090872	0.862134
H	-5.334765	-5.756835	1.571538
S	-4.994955	-5.130065	-0.779269
H	5.970393	-5.448255	-3.380126
N	6.287742	-2.058148	-3.290372
H	5.803827	-1.772527	-2.436731
C	6.608842	-3.344659	-3.650310
C	6.358972	-4.571488	-2.816660
H	5.663994	-4.318147	-2.021085
H	7.305683	-4.842250	-2.353391
N	7.350793	-1.948716	-5.224800
C	7.270108	-3.291791	-4.847478
H	7.685526	-4.132721	-5.383620
C	6.745577	-1.244889	-4.255930
H	6.641990	-0.170511	-4.262120
H	-6.136525	0.246825	-6.237978
C	-5.795479	0.076367	-5.188140
H	-5.324438	-0.906013	-5.139295
H	-5.013005	0.807365	-4.983643
S	-7.075478	0.210501	-3.844921
H	8.304239	4.588567	-2.863832
N	6.328086	1.107080	-1.885152
H	6.281240	0.123829	-1.602581
C	7.253325	2.042190	-1.500568
H	8.177507	1.864917	-0.962930
N	5.632976	3.015702	-2.720497
C	6.826762	3.221230	-2.025147
C	5.391294	1.696241	-2.640132
H	4.538613	1.176271	-3.062829
C	7.617540	4.492968	-1.992753
H	8.198469	4.493880	-1.068111
H	6.938005	5.345477	-1.959952
H	3.280552	5.233197	-4.090183
C	2.958407	4.833666	-3.105051
H	2.846308	3.753189	-3.201628
H	3.749182	5.026948	-2.377119

C	1.641364	5.468243	-2.657763
H	1.730801	6.548188	-2.511309
H	0.873843	5.343281	-3.428895
C	1.004256	4.929351	-1.388139
O	-0.025916	5.456191	-0.956224
O	1.480496	3.898133	-0.761883
H	2.453345	3.525599	-0.892997
H	2.248151	6.548204	2.351243
C	2.599335	5.499325	2.239958
H	2.206626	4.891465	3.053952
H	2.241207	5.100269	1.296151
C	4.123390	5.433302	2.265029
O	4.780372	6.018063	3.105695
N	4.763103	4.755647	1.337758
H	5.757769	4.941032	1.328406
H	4.279344	4.221862	0.624817
H	-2.127672	4.196500	1.793647
C	-2.515390	3.714694	0.864294
H	-3.168951	2.890143	1.147365
O	-1.416503	3.135579	0.149106
H	-0.915462	3.867614	-0.241406
C	-3.264357	4.676788	-0.051138
H	-3.756078	4.115895	-0.845515
H	-4.036608	5.244209	0.478723
H	-2.580959	5.401377	-0.499013
H	0.694891	1.789314	3.728224
C	1.353411	1.244146	3.018748
H	1.959270	0.517808	3.555110
O	2.200819	2.140752	2.331933
H	2.479452	1.680222	1.531333
C	0.514318	0.521913	1.970710
H	1.153338	-0.037411	1.293140
H	-0.174294	-0.170346	2.455220
H	-0.061606	1.242696	1.398271
H	-7.386207	1.946790	3.855390
C	-7.212979	1.307467	2.969735
H	-8.141857	0.787322	2.731428
H	-6.985177	1.956763	2.125495
S	-5.848294	0.092898	3.225649
H	7.182470	-2.908605	0.833059
C	7.116872	-2.209659	1.689699
O	7.602155	-2.484754	2.791345
N	6.461700	-1.074184	1.473625
H	6.072414	-0.971739	0.539407
C	6.189972	-0.053077	2.485806
H	7.144242	0.306419	2.871329
C	5.479030	1.123882	1.819877
H	6.030310	1.415944	0.926185
H	4.467673	0.837494	1.534605
O	5.433217	2.220810	2.701680
H	5.074071	2.979136	2.218635
C	5.387186	-0.603718	3.674914
O	5.797719	-0.447246	4.826503
N	4.248973	-1.260225	3.424909
H	3.974340	-1.376694	2.452061
C	3.384073	-1.829026	4.455751
H	3.160923	-1.005070	5.131989
C	2.031566	-2.305410	3.885199
H	1.303109	-2.310737	4.690922
H	1.676358	-1.613997	3.130506
C	2.018525	-3.692431	3.280207
O	1.670946	-4.667737	3.921600
N	2.382171	-3.811756	2.033477
H	2.465348	-4.718490	1.616348
H	2.665694	-2.979453	1.516218
H	3.906550	-2.592291	5.070293
O	3.665447	2.798336	-0.537384
H	4.389870	2.947987	-1.177169
H	3.409064	1.797047	-0.679541
O	1.390666	-1.049110	7.114496
H	1.683002	-0.273735	6.607113
H	2.133852	-1.671394	7.060659
O	-0.552790	-3.309256	6.396189
H	-0.061870	-2.508844	6.617963
H	-0.736001	-3.238541	5.443130
O	-0.107071	8.205500	-1.450984
H	-0.619132	8.594474	-0.718425
H	-0.237603	7.244635	-1.327770
O	-6.594342	4.333408	0.819984
H	-5.907523	4.665845	1.429746
H	-6.192138	3.522628	0.447305
O	-1.274350	-2.465686	3.763796
H	-1.744613	-1.664226	4.056331
H	-1.723483	-2.653858	2.925000
O	-8.987531	5.171843	2.267029
H	-8.632227	5.658480	3.036783
H	-8.186597	4.814119	1.836624
O	-7.641376	6.975355	-0.147169
H	-7.107946	7.746054	0.143379
H	-7.046308	6.215439	-0.014040
C	-0.703446	0.391630	-2.277869
O	-0.911168	0.671011	-0.901047
C	-0.456571	-1.078641	-2.474897
C	-0.356756	-1.927858	-1.455941
C	-0.137941	-3.407401	-1.582547
O	1.060592	-3.841532	-0.936605
P	2.532866	-3.616656	-1.668482
O	2.704680	-1.952790	-1.588941
P	3.602085	-1.126682	-0.522359
O	2.888570	0.430178	-0.885039
O	5.033124	-1.033975	-0.981102
O	3.225623	-1.426076	0.896047

O	2.447280	-3.878785	-3.152592
O	3.527349	-4.278417	-0.753272
C	-0.369267	-1.490629	-3.913048
H	0.168533	0.952569	-2.654938
H	-1.573101	0.691888	-2.877667
H	-1.124173	1.607007	-0.726041
H	-0.419238	-1.553443	-0.441408
H	-0.956995	-3.923829	-1.072461
H	-0.144455	-3.728746	-2.627582
H	0.393332	-2.253865	-4.070510
H	-1.339677	-1.872529	-4.247301
H	-0.127149	-0.631077	-4.548345
H	1.922177	0.359095	-0.836657
Fe	-4.844616	-2.914035	-0.379419
Fe	-3.440720	-0.480819	-0.658664
Fe	-5.455288	-0.540101	1.026641
Fe	-6.034976	-0.358738	-1.864754
S	-3.491391	-1.836742	1.247177
S	-5.018655	1.309575	-0.412407
S	-4.099018	-1.742246	-2.386176
S	-6.996457	-1.801580	-0.142744

I1

H	-6.813519	-6.766090	0.564532
C	-6.382074	-5.763104	0.749346
H	-7.189898	-5.084324	1.023750
H	-5.708683	-5.831186	1.608502
S	-5.510101	-5.120057	-0.743998
H	5.401349	-5.441331	-3.735768
N	5.865336	-2.072978	-3.442883
H	5.422799	-1.819903	-2.557771
C	6.104651	-3.347617	-3.896011
C	5.824964	-4.612054	-3.128679
H	5.143653	-4.384482	-2.311381
H	6.769057	-4.938397	-2.696294
N	6.864997	-1.888397	-5.403621
C	6.723869	-3.248021	-5.112988
H	7.075136	-4.069879	-5.720053
C	6.336279	-1.220065	-4.366878
H	6.294445	-0.143272	-4.294689
H	-6.234520	0.840691	-5.876199
C	-5.812786	0.598630	-4.871201
H	-5.324276	-0.374622	-4.944012
H	-5.029528	1.332438	-4.672739
S	-6.966001	0.584798	-3.409006
H	8.184364	4.395183	-2.737658
N	5.798564	1.126595	-1.966314
H	5.651231	0.133036	-1.771238
C	6.838542	1.905565	-1.526679
H	7.739366	1.571993	-1.024897
N	5.350759	3.167960	-2.640957
C	6.564028	3.167107	-1.951033
C	4.944339	1.887874	-2.662598
H	4.035627	1.518332	-3.122494
C	7.515604	4.320568	-1.850971
H	8.118333	4.176719	-0.952080
H	6.952803	5.247378	-1.732795
H	3.159113	5.538895	-3.980005
C	2.770739	5.153328	-3.012781
H	2.549968	4.091889	-3.126229
H	3.557178	5.254381	-2.260535
C	1.512107	5.901418	-2.563863
H	1.697187	6.970029	-2.428680
H	0.733109	5.829357	-3.332104
C	0.873967	5.387651	-1.266304
O	0.013586	6.112701	-0.711534
O	1.203935	4.249734	-0.825143
H	2.667027	3.532622	-0.599967
H	2.561700	6.452310	2.563002
C	2.874856	5.413713	2.314369
H	2.414950	4.710575	3.008455
H	2.553086	5.177452	1.301756
C	4.393709	5.273610	2.406146
O	5.042074	5.801410	3.289863
N	5.040885	4.585262	1.494465
H	6.041297	4.735067	1.506669
H	4.544927	4.100850	0.754880
H	-1.736083	4.129348	2.008148
C	-2.040284	3.574677	1.090381
H	-2.658972	2.730674	1.396993
O	-0.892324	3.001967	0.459043
H	-0.282675	3.677372	0.092867
C	-2.839048	4.450304	0.128764
H	-3.285194	3.826737	-0.645308
H	-3.656876	4.951380	0.669262
H	-2.215381	5.223956	-0.323056
H	0.724031	1.464864	3.927929
C	1.186694	0.725650	3.242497
H	1.713626	-0.017204	3.831606
O	2.102226	1.324383	2.359197
H	2.351835	0.614583	1.742052
C	0.119286	0.036352	2.389500
H	0.574504	-0.664135	1.695155
H	-0.558620	-0.518369	3.029201
H	-0.456479	0.766133	1.824945
H	-6.667480	1.990561	3.863884
C	-6.415314	1.331915	3.012607
H	-7.296314	0.732699	2.779755
H	-6.217971	1.969829	2.149389
S	-4.987791	0.199207	3.279511
H	6.865542	-3.156875	0.486319
C	6.873166	-2.501449	1.377768
O	7.419634	-2.846220	2.430374

N	6.233207	-1.342254	1.263753
H	5.795005	-1.168041	0.362745
C	6.062249	-0.384586	2.356134
H	7.054655	-0.135912	2.733261
C	5.454558	0.905188	1.805910
H	5.986682	1.185900	0.898364
H	4.407602	0.760371	1.562102
O	5.589744	1.951220	2.737747
H	5.274592	2.763543	2.314925
C	5.270139	-0.979116	3.535276
O	5.735323	-0.900516	4.674285
N	4.111906	-1.618167	3.301812
H	3.761252	-1.616702	2.347503
C	3.346903	-2.293458	4.358564
H	3.158650	-1.523722	5.104085
C	1.960312	-2.820558	3.929692
H	1.475410	-3.235875	4.809847
H	1.330764	-2.017379	3.579546
C	1.937722	-3.901440	2.877446
O	1.801543	-5.074446	3.166378
N	2.010242	-3.521206	1.634176
H	2.089509	-4.214539	0.902816
H	2.149614	-2.540282	1.413964
H	3.933736	-3.070257	4.888285
O	3.494262	3.021163	-0.363062
H	4.103024	3.225560	-1.099839
H	3.041041	1.416017	-0.760216
O	1.490036	-1.268495	7.030272
H	1.816417	-0.465108	6.587532
H	2.225775	-1.895913	6.976412
O	-0.463490	-3.491227	6.537187
H	-0.018302	-2.645734	6.672669
H	-0.784588	-3.468784	5.619779
O	0.433478	8.760510	-0.924724
H	-0.062926	9.116658	-0.164776
H	0.202717	7.795646	-0.887956
O	-6.354558	5.726507	0.872634
H	-5.683408	5.952371	1.548652
H	-5.868549	5.100080	0.321023
O	-1.523941	-2.837731	3.990482
H	-1.796678	-1.945196	4.262379
H	-1.886377	-2.912130	3.090988
O	-8.883137	6.059254	2.325758
H	-8.671212	6.884964	2.815974
H	-8.007094	5.794752	1.993079
O	-7.632033	8.124492	0.055644
H	-7.107528	8.888279	0.392487
H	-7.003107	7.391869	0.203639
C	-0.578226	0.740194	-2.044795
O	0.097759	0.866787	-0.797956
C	-0.950766	-0.701302	-2.347618
C	-0.731835	-1.703817	-1.446546
C	-0.628413	-3.184644	-1.755901
O	0.550793	-3.746941	-1.177210
P	2.055857	-3.493438	-1.810016
O	2.189190	-1.825098	-1.487402
P	3.242946	-1.058723	-0.563516
O	2.602558	0.596181	-1.127768
O	4.641313	-1.136381	-1.092046
O	2.891038	-1.026271	0.890564
O	2.069695	-3.594423	-3.309013
O	2.998435	-4.239972	-0.905787
C	-1.171261	-0.938824	-3.816396
H	0.095921	1.074722	-2.845017
H	-1.451063	1.396110	-2.053616
H	-0.327308	1.575831	-0.258810
H	-0.362172	-1.431722	-0.467422
H	-1.459325	-3.723762	-1.300358
H	-0.651970	-3.367169	-2.833753
H	-0.201439	-0.981938	-4.323409
H	-1.709651	-1.862272	-4.008591
H	-1.753655	-0.126456	-4.262912
H	1.595472	0.717571	-0.953094
Fe	-4.867052	-3.013176	-0.249621
Fe	-2.902525	-1.034554	-1.222650
Fe	-4.782235	-0.577507	1.098923
Fe	-5.670401	-0.413681	-1.758856
S	-3.090519	-2.162865	1.025089
S	-4.098840	0.949090	-0.555911
S	-4.295851	-2.233969	-2.522574
S	-6.679725	-1.518884	0.220054

TS2

H	-6.867669	-6.796666	0.628323
C	-6.452248	-5.785352	0.798627
H	-7.274086	-5.106128	1.024991
H	-5.805334	-5.821877	1.679237
S	-5.535616	-5.186568	-0.685841
H	5.539924	-5.565131	-3.466315
N	6.041362	-2.207098	-3.365758
H	5.568028	-1.923126	-2.505892
C	6.291646	-3.504012	-3.733786
C	5.975738	-4.714967	-2.899626
H	5.291090	-4.427372	-2.103542
H	6.907899	-5.036059	-2.439080
N	7.104964	-2.137785	-5.299461
C	6.952535	-3.478115	-4.931966
H	7.322071	-4.335908	-5.474727
C	6.543384	-1.409078	-4.322241
H	6.496948	-0.329979	-4.319456
H	-6.105855	0.637268	-5.991939
C	-5.716639	0.411019	-4.971485
H	-5.220907	-0.560033	-5.015867

H	-4.945987	1.152129	-4.752375
S	-6.928744	0.409270	-3.558861
H	8.371120	4.258586	-2.763034
N	6.005396	0.933684	-1.994554
H	5.851650	-0.054763	-1.777379
C	7.026803	1.726317	-1.533246
H	7.901388	1.407767	-0.977874
N	5.592590	2.960541	-2.748286
C	6.772767	2.977042	-2.004200
C	5.189598	1.680046	-2.754546
H	4.308556	1.301164	-3.257470
C	7.704376	4.144336	-1.879240
H	8.311765	3.990561	-0.985137
H	7.123094	5.056489	-1.736629
H	3.322351	5.328159	-4.134174
C	2.932580	4.944456	-3.168349
H	2.737183	3.877531	-3.274035
H	3.711808	5.060381	-2.411252
C	1.654673	5.658520	-2.719585
H	1.821774	6.720377	-2.520906
H	0.893995	5.620730	-3.508810
C	1.008384	5.041465	-1.470829
O	0.095379	5.688427	-0.886748
O	1.399263	3.905037	-1.099047
H	3.018607	3.418553	-0.728212
H	2.519146	6.550188	2.395368
C	2.862889	5.508545	2.200622
H	2.377193	4.821362	2.893244
H	2.604942	5.227778	1.179794
C	4.378221	5.412603	2.390643
O	4.941852	5.917697	3.342700
N	5.119255	4.804202	1.493971
H	6.107353	5.014750	1.565277
H	4.700164	4.324988	0.704987
H	-1.947234	4.347231	1.898072
C	-2.334882	3.824500	0.991373
H	-2.976991	3.007632	1.325358
O	-1.264530	3.227978	0.274939
H	-0.713263	3.950834	-0.072889
C	-3.128748	4.740013	0.059513
H	-3.624897	4.133315	-0.699604
H	-3.891790	5.313437	0.602705
H	-2.470662	5.456700	-0.435880
H	0.604014	1.690724	3.848778
C	1.119032	0.955974	3.195650
H	1.625040	0.223728	3.816563
O	2.072283	1.563126	2.357683
H	2.330849	0.873824	1.722670
C	0.106021	0.243983	2.301535
H	0.610421	-0.447643	1.630869
H	-0.597683	-0.314346	2.913098

H	-0.447236	0.972690	1.714986
H	-6.886859	2.123998	3.730184
C	-6.637650	1.450500	2.889890
H	-7.532964	0.875865	2.651267
H	-6.405623	2.074040	2.024869
S	-5.253440	0.275099	3.196648
H	6.927427	-3.110221	0.721658
C	6.892662	-2.418409	1.585875
O	7.394425	-2.720804	2.673251
N	6.260208	-1.262756	1.401505
H	5.848636	-1.139439	0.479963
C	6.059940	-0.255642	2.447508
H	7.041856	-0.001279	2.847590
C	5.480523	1.022298	1.836159
H	6.013339	1.245536	0.912779
H	4.427557	0.895366	1.607945
O	5.642128	2.112501	2.714047
H	5.373597	2.913247	2.238848
C	5.224188	-0.797602	3.620962
O	5.653028	-0.687307	4.771377
N	4.065507	-1.427698	3.369678
H	3.751610	-1.448712	2.402419
C	3.255751	-2.061818	4.419244
H	3.041792	-1.265775	5.129770
C	1.885073	-2.592695	3.947424
H	1.353597	-2.975611	4.816180
H	1.283487	-1.793998	3.541902
C	1.903335	-3.702009	2.924389
O	1.716454	-4.864171	3.234062
N	2.071070	-3.354348	1.679694
H	2.175945	-4.074849	0.975908
H	2.247879	-2.385171	1.433572
H	3.814717	-2.824140	5.000062
O	3.874355	3.062237	-0.386494
H	4.475406	3.144093	-1.152803
H	3.421058	1.229232	-0.912467
O	1.316328	-1.015082	7.033853
H	1.620650	-0.211896	6.575420
H	2.074502	-1.617417	7.002370
O	-0.612248	-3.265784	6.606422
H	-0.142719	-2.431379	6.730025
H	-0.887574	-3.267504	5.674820
O	0.198191	8.386715	-1.283229
H	-0.296901	8.745669	-0.524325
H	0.091408	7.408386	-1.178334
O	-6.524994	5.815544	0.573728
H	-5.899712	6.023922	1.298188
H	-5.973120	5.258237	0.009641
O	-1.517077	-2.640957	3.996144
H	-1.805252	-1.743306	4.234545
H	-1.945905	-2.787501	3.138257

O	-9.058092	6.168772	1.996413
H	-8.848087	7.008502	2.463496
H	-8.180082	5.891855	1.681093
O	-7.739344	8.181707	-0.338030
H	-7.241744	8.955310	0.014784
H	-7.119735	7.455654	-0.128742
C	-1.052695	0.603839	-1.237633
O	0.670357	1.283306	-0.731852
C	-0.902814	-0.636026	-1.933598
C	-0.785403	-1.810162	-1.195186
C	-0.500707	-3.190788	-1.749469
O	0.629010	-3.770363	-1.127386
P	2.181951	-3.490773	-1.712096
O	2.260277	-1.857002	-1.407613
P	3.384202	-0.992178	-0.586149
O	2.959237	0.479664	-1.340831
O	4.781865	-1.326732	-1.036580
O	2.993367	-0.873982	0.866025
O	2.206666	-3.638007	-3.211981
O	3.067236	-4.274403	-0.781294
C	-0.764564	-0.631526	-3.438912
H	-1.367188	1.481548	-1.776117
H	-1.323990	0.602859	-0.197864
H	0.711464	2.257537	-0.793421
H	-0.612594	-1.725009	-0.128277
H	-1.352198	-3.834901	-1.515922
H	-0.394292	-3.169278	-2.839029
H	0.134152	-1.170884	-3.738616
H	-1.621133	-1.110685	-3.914449
H	-0.703638	0.391405	-3.816794
H	1.530644	0.892748	-1.110513
Fe	-4.974391	-3.063668	-0.235525
Fe	-2.939484	-1.183795	-1.083953
Fe	-5.035345	-0.582041	1.059117
Fe	-5.709330	-0.539491	-1.845910
S	-3.300835	-2.153635	1.121406
S	-4.231597	0.850826	-0.606548
S	-4.226113	-2.347464	-2.480043
S	-6.836569	-1.606116	0.088735

I2

H	-6.629226	-6.788847	0.397590
C	-6.201748	-5.785506	0.588119
H	-7.017436	-5.099196	0.815046
H	-5.566067	-5.846182	1.474854
S	-5.259251	-5.160088	-0.868878
H	5.585346	-5.434345	-3.652341
N	5.945887	-2.066064	-3.359437
H	5.430838	-1.843380	-2.505250
C	6.253636	-3.329847	-3.797300
C	5.980589	-4.598143	-3.037756
H	5.278667	-4.383225	-2.235063
H	6.921503	-4.905555	-2.585644
N	7.053896	-1.846817	-5.258085
C	6.937498	-3.210730	-4.976966
H	7.349839	-4.020546	-5.561711
C	6.446790	-1.196685	-4.252574
H	6.363693	-0.121875	-4.187307
H	-5.973552	0.740785	-5.934845
C	-5.570777	0.491862	-4.925020
H	-5.073601	-0.476449	-4.995072
H	-4.802237	1.230110	-4.693693
S	-6.781774	0.456834	-3.511488
H	8.115291	4.334797	-2.466026
N	5.627027	1.079022	-1.929562
H	5.453292	0.079469	-1.797341
C	6.688642	1.798319	-1.439106
H	7.574215	1.407221	-0.951348
N	5.247791	3.172671	-2.476446
C	6.454414	3.093815	-1.779337
C	4.804368	1.909820	-2.584407
H	3.895671	1.599416	-3.086676
C	7.434177	4.215957	-1.593621
H	8.024983	4.000707	-0.701391
H	6.890573	5.146116	-1.421663
H	3.114827	5.552359	-3.891354
C	2.700726	5.161879	-2.936190
H	2.504475	4.097044	-3.064137
H	3.454667	5.285031	-2.153863
C	1.405970	5.885237	-2.546399
H	1.552185	6.962046	-2.433507
H	0.659624	5.762579	-3.341169
C	0.729111	5.387944	-1.261497
O	-0.044542	6.174902	-0.664325
O	0.941509	4.202258	-0.870481
H	2.548891	3.545055	-0.464503
H	2.452424	6.503930	2.629542
C	2.786067	5.475330	2.366068
H	2.315352	4.750240	3.029948
H	2.495496	5.260294	1.338535
C	4.304132	5.354032	2.501934
O	4.908670	5.825031	3.446808
N	4.996637	4.746281	1.566341
H	5.991547	4.929266	1.612441
H	4.532569	4.304182	0.781806
H	-1.895772	4.204417	1.999982
C	-2.247479	3.681482	1.078457
H	-2.980253	2.935363	1.393742
O	-1.208819	2.952002	0.455168
H	-0.527926	3.546892	0.078374
C	-2.922899	4.652784	0.107487
H	-3.457479	4.091478	-0.661348
H	-3.640112	5.293330	0.643951

H	-2.194793	5.315249	-0.363522
H	0.523075	1.485333	3.964823
C	1.002974	0.734007	3.304697
H	1.466228	-0.023993	3.927287
O	1.989744	1.305803	2.483065
H	2.279997	0.586159	1.895456
C	-0.016925	0.073645	2.374750
H	0.468378	-0.653107	1.729427
H	-0.769700	-0.443432	2.961449
H	-0.507896	0.820162	1.756759
H	-6.912076	1.867842	3.843274
C	-6.646565	1.215362	2.991886
H	-7.532594	0.637171	2.728548
H	-6.409759	1.863390	2.147257
S	-5.251181	0.049528	3.282672
H	6.898592	-3.042224	0.551359
C	6.867522	-2.368739	1.429076
O	7.434325	-2.664050	2.485749
N	6.159250	-1.249802	1.301671
H	5.686919	-1.128590	0.409166
C	5.960027	-0.288006	2.389338
H	6.944015	-0.021473	2.776513
C	5.336280	0.993517	1.836630
H	5.874428	1.282884	0.937538
H	4.294428	0.834765	1.580368
O	5.445925	2.044628	2.766656
H	5.175492	2.861328	2.320944
C	5.168235	-0.894552	3.561789
O	5.615741	-0.806099	4.707107
N	4.026647	-1.554871	3.311548
H	3.696453	-1.562533	2.350096
C	3.249788	-2.239669	4.352576
H	3.020910	-1.471251	5.088515
C	1.891015	-2.797151	3.876621
H	1.371168	-3.205263	4.741170
H	1.268424	-2.010611	3.479461
C	1.938776	-3.891181	2.839517
O	1.820497	-5.062435	3.140967
N	2.049264	-3.524206	1.594285
H	2.186355	-4.227911	0.879263
H	2.178652	-2.543660	1.366765
H	3.840080	-3.001069	4.902545
O	3.382759	3.108505	-0.174390
H	3.946840	3.154224	-0.968845
H	2.772584	1.217182	-0.628848
O	1.317490	-1.264563	7.029496
H	1.614108	-0.444936	6.595192
H	2.082777	-1.856562	6.981858
O	-0.558994	-3.519638	6.574388
H	-0.107424	-2.678397	6.717529
H	-0.850416	-3.497425	5.648793
O	0.388530	8.806569	-0.851350
H	-0.136651	9.136649	-0.099368
H	0.183472	7.833057	-0.830759
O	-6.243008	5.681650	0.595414
H	-5.667786	5.893353	1.359123
H	-5.609237	5.259899	-0.000325
O	-1.592272	-2.845394	4.033749
H	-1.875509	-1.952484	4.293393
H	-1.872096	-2.911074	3.108790
O	-8.723455	5.807779	2.074297
H	-8.542413	6.707554	2.419462
H	-7.876148	5.574636	1.657576
O	-7.720261	8.015762	-0.034303
H	-7.232704	8.796558	0.318898
H	-7.035124	7.322560	0.044447
C	-1.122842	0.409366	-1.491357
O	1.053890	2.169342	-2.887178
C	-0.733179	-0.776937	-2.121383
C	-0.583045	-1.916891	-1.323521
C	-0.459032	-3.331099	-1.824907
O	0.649238	-3.998838	-1.271001
P	2.194715	-3.549114	-1.797942
O	2.062644	-1.948592	-1.371254
P	3.130729	-0.967922	-0.569438
O	2.558495	0.468051	-1.217603
O	4.524625	-1.259851	-1.071295
O	2.799873	-0.954037	0.905566
O	2.257653	-3.601253	-3.304438
O	3.109429	-4.328024	-0.894875
C	-0.667367	-0.860969	-3.619679
H	-1.314815	1.298952	-2.067628
H	-0.917645	0.576698	-0.439302
H	0.898347	2.847854	-2.208758
H	-0.411860	-1.785165	-0.261405
H	-1.353787	-3.866923	-1.493226
H	-0.444564	-3.359273	-2.919633
H	0.177823	-1.483681	-3.913798
H	-1.579275	-1.305496	-4.020544
H	-0.544239	0.137645	-4.038285
H	1.541188	1.469578	-2.414841
Fe	-4.720357	-3.048764	-0.336516
Fe	-2.786837	-0.945170	-1.032285
Fe	-4.944818	-0.662178	1.117564
Fe	-5.613574	-0.478615	-1.772497
S	-3.098286	-2.117968	1.064809
S	-4.178184	0.896093	-0.465008
S	-4.000801	-2.145942	-2.474134
S	-6.675929	-1.712664	0.061779

I3

H	-6.471586	-6.840539	0.477906
C	-6.037339	-5.832255	0.635029

H	-6.839354	-5.161650	0.941018
H	-5.326284	-5.888720	1.462318
S	-5.234089	-5.167930	-0.887173
H	5.585452	-5.462933	-3.740253
N	5.915647	-2.087126	-3.349705
H	5.415404	-1.883698	-2.481980
C	6.204663	-3.334589	-3.844986
C	5.971560	-4.628899	-3.116508
H	5.280161	-4.448566	-2.297141
H	6.927730	-4.925197	-2.687998
N	6.996480	-1.796503	-5.254007
C	6.862949	-3.170925	-5.033575
H	7.261775	-3.959830	-5.655207
C	6.417281	-1.185865	-4.207825
H	6.359418	-0.115875	-4.079037
H	-5.978965	0.442935	-5.766267
C	-5.573915	0.202985	-4.757401
H	-5.031307	-0.738441	-4.831480
H	-4.849681	0.975340	-4.499220
S	-6.798836	0.062423	-3.362975
H	8.005720	4.399852	-2.258286
N	5.501357	1.159179	-1.857657
H	5.334119	0.153454	-1.769354
C	6.542756	1.870067	-1.315705
H	7.419246	1.469788	-0.819813
N	5.120985	3.265957	-2.352430
C	6.312091	3.173006	-1.630093
C	4.687840	2.003955	-2.504722
H	3.796773	1.699071	-3.040915
C	7.293267	4.288358	-1.410575
H	7.851232	4.071256	-0.498267
H	6.750954	5.221777	-1.262373
H	2.986674	5.536227	-3.951852
C	2.503436	5.135178	-3.035575
H	2.287412	4.079102	-3.196903
H	3.212661	5.214084	-2.207243
C	1.216455	5.892612	-2.688381
H	1.361912	6.975062	-2.646837
H	0.462680	5.717766	-3.464861
C	0.574625	5.480809	-1.358858
O	-0.135456	6.323587	-0.757281
O	0.766192	4.303770	-0.931244
H	2.355846	3.579461	-0.414901
H	2.671698	6.686925	2.428567
C	3.005595	5.676035	2.108235
H	2.508104	4.913642	2.707656
H	2.733272	5.537643	1.062259
C	4.512471	5.484014	2.257126
O	5.175824	6.062915	3.104295
N	5.113057	4.623200	1.474083
H	6.105865	4.527222	1.597540

H	4.560908	3.992972	0.901943
H	-1.822236	4.377327	2.006883
C	-2.270917	3.869729	1.118151
H	-3.001551	3.150594	1.499617
O	-1.324170	3.108123	0.406551
H	-0.627954	3.678324	0.014409
C	-3.006948	4.866583	0.220365
H	-3.586500	4.325985	-0.532486
H	-3.688479	5.482314	0.830735
H	-2.314608	5.545597	-0.280736
H	0.601504	1.585680	3.944774
C	0.992253	0.770681	3.300941
H	1.339784	-0.029438	3.947028
O	2.055139	1.196576	2.484816
H	2.212012	0.456571	1.867494
C	-0.061275	0.220981	2.342712
H	0.362872	-0.563926	1.721389
H	-0.890560	-0.199391	2.897460
H	-0.437691	1.019669	1.711448
H	-6.765410	1.862332	3.882888
C	-6.494548	1.193771	3.044861
H	-7.371852	0.592295	2.802215
H	-6.278903	1.823233	2.180750
S	-5.073815	0.065322	3.353235
H	6.957851	-2.944244	0.409563
C	6.961541	-2.249395	1.272798
O	7.567381	-2.530142	2.310701
N	6.246221	-1.129764	1.158601
H	5.734425	-1.030972	0.285386
C	6.003009	-0.206720	2.273970
H	6.968396	0.117357	2.663320
C	5.271656	1.049014	1.788487
H	5.970424	1.655022	1.214879
H	4.442896	0.776969	1.140743
O	4.778988	1.825806	2.860251
H	3.813902	1.697985	2.874317
C	5.282674	-0.907374	3.432658
O	5.794642	-0.892133	4.553324
N	4.125839	-1.541802	3.193032
H	3.752108	-1.512618	2.248290
C	3.361496	-2.216235	4.244046
H	3.137926	-1.435954	4.968574
C	1.997839	-2.759337	3.773009
H	1.448823	-3.101271	4.647058
H	1.415745	-1.973336	3.312689
C	2.032331	-3.910608	2.800138
O	1.955106	-5.065342	3.169954
N	2.082989	-3.608867	1.533775
H	2.222205	-4.335846	0.844519
H	2.189006	-2.634379	1.273354
H	3.961620	-2.966068	4.801059

O	3.164130	3.110426	-0.111956
H	3.754985	3.181396	-0.884670
H	1.441834	1.502186	-2.460649
O	1.636681	-1.212189	7.018751
H	1.879753	-0.393996	6.546985
H	2.412170	-1.785891	6.922739
O	-0.346694	-3.415110	6.556817
H	0.169738	-2.617020	6.727098
H	-0.655903	-3.331221	5.639963
O	0.357073	8.954332	-0.793744
H	-0.185495	9.203962	-0.028092
H	0.148887	7.985612	-0.881232
O	-6.330236	5.717787	0.762630
H	-5.928915	6.172720	1.532331
H	-5.542724	5.331164	0.351684
O	-1.341026	-2.623514	4.021309
H	-1.639823	-1.738384	4.290265
H	-1.780697	-2.766790	3.168976
O	-8.995533	5.825305	1.825053
H	-8.741730	6.743540	2.093640
H	-8.122639	5.509366	1.527570
O	-7.736198	7.868428	-0.603787
H	-7.581451	8.703940	-0.107219
H	-7.063744	7.278200	-0.214800
C	-1.054745	0.358338	-1.326921
O	0.974623	2.215583	-2.930592
C	-0.757929	-0.842284	-1.992665
C	-0.626179	-1.999552	-1.220234
C	-0.504972	-3.402015	-1.758230
O	0.657196	-4.057071	-1.295741
P	2.178643	-3.594617	-1.872742
O	2.060101	-1.991609	-1.463293
P	3.073388	-1.002475	-0.619706
O	2.478306	0.433871	-1.256079
O	4.489625	-1.240379	-1.086782
O	2.715191	-1.003105	0.852047
O	2.213805	-3.653458	-3.381342
O	3.127500	-4.358689	-0.989145
C	-0.749525	-0.895830	-3.496008
H	-1.211951	1.269415	-1.878328
H	-0.744198	0.489661	-0.299987
H	0.791821	2.877762	-2.243233
H	-0.381169	-1.894901	-0.170819
H	-1.354332	-3.975298	-1.374952
H	-0.563201	-3.411099	-2.852632
H	-1.674100	-1.330379	-3.877314
H	-0.640371	0.110077	-3.901109
H	0.086542	-1.510883	-3.830710
H	2.623443	1.159485	-0.622666
Fe	-4.708365	-3.037962	-0.337605
Fe	-2.714980	-0.908685	-0.911563
Fe	-4.774235	-0.691662	1.200436
Fe	-5.622423	-0.616353	-1.571081
S	-3.079227	-2.243406	1.118777
S	-4.237251	0.848604	-0.436834
S	-4.088630	-2.069789	-2.426708
S	-6.640730	-1.730129	0.218732

TS3 (C2 protonation)

H	-4.739124	3.199956	8.591657
C	-3.969899	2.638291	8.022932
H	-3.326481	2.127501	8.736856
H	-4.472824	1.872461	7.428408
S	-2.935063	3.725800	6.953043
H	-4.283683	5.988962	-4.030605
N	-1.491093	4.204428	-4.912755
H	-1.537681	3.344878	-4.356981
C	-2.449003	5.185288	-5.019261
C	-3.863871	5.078379	-4.510539
H	-3.911457	4.257730	-3.800245
H	-4.483270	4.814415	-5.363208
N	-0.645786	5.789484	-6.190611
C	-1.929105	6.185575	-5.794999
H	-2.444243	7.084294	-6.102422
C	-0.433596	4.581015	-5.642976
H	0.464649	3.991119	-5.757703
H	4.158530	6.054581	6.609379
C	3.437932	5.231832	6.399389
H	2.515418	5.684648	6.037786
H	3.846353	4.641257	5.579706
S	3.022970	4.072601	7.794836
H	3.799432	0.463923	-7.662888
N	1.229337	1.241864	-4.531327
H	0.322045	1.570704	-4.188877
C	1.462610	0.573746	-5.706313
H	0.749591	0.357546	-6.489870
N	3.396242	0.836453	-4.591949
C	2.796810	0.316273	-5.736242
C	2.395237	1.388834	-3.886656
H	2.499771	1.882622	-2.931501
C	3.519128	-0.291109	-6.899025
H	2.864570	-1.037516	-7.351982
H	4.413114	-0.799218	-6.538406
H	6.295608	1.850899	-2.878044
C	5.695394	1.208290	-2.200593
H	4.813971	1.767608	-1.891786
H	5.349324	0.333849	-2.755519
C	6.506643	0.770632	-0.970118
H	7.463096	0.315418	-1.237368
H	6.735130	1.646938	-0.351669
C	5.749355	-0.225419	-0.087785
O	6.344996	-1.239102	0.347016

O	4.527963	0.017351	0.153771
H	3.343095	-0.544850	-1.165517
H	4.976902	-4.317472	-1.972389
C	4.227221	-3.537249	-2.215071
H	3.381943	-3.615803	-1.531698
H	4.700855	-2.563322	-2.092450
C	3.697201	-3.645869	-3.643596
O	3.725237	-4.692547	-4.280083
N	3.141233	-2.589726	-4.185274
H	2.842884	-2.648589	-5.140251
H	3.014153	-1.741054	-3.629882
H	3.720820	-2.884408	2.672793
C	3.703388	-1.853594	3.105355
H	3.086703	-1.903156	4.007714
O	3.060429	-0.939230	2.243630
H	3.670009	-0.677937	1.517090
C	5.101479	-1.365543	3.485761
H	5.018759	-0.457976	4.089488
H	5.648492	-2.130311	4.055113
H	5.702023	-1.154563	2.600083
H	0.055184	-3.415464	0.749438
C	-0.393526	-2.511649	0.283533
H	-1.375840	-2.750916	-0.113577
O	0.416064	-2.014982	-0.752745
H	-0.142075	-1.398464	-1.266246
C	-0.531817	-1.381280	1.299079
H	-0.856886	-0.468318	0.803551
H	-1.265248	-1.660060	2.051427
H	0.421358	-1.208328	1.792784
H	1.736977	-3.483668	8.186806
C	1.437483	-2.443058	7.966479
H	1.191756	-1.959138	8.913246
H	2.309458	-1.930879	7.556570
S	0.013826	-2.267641	6.803145
H	-4.020377	1.202053	-5.163108
C	-3.793902	0.117973	-5.175244
O	-4.683267	-0.702199	-5.439802
N	-2.551152	-0.285574	-4.889986
H	-1.881672	0.420122	-4.582448
C	-2.227906	-1.721246	-4.881509
H	-2.643105	-2.130957	-5.801320
C	-0.733152	-2.031151	-4.965402
H	-0.620005	-3.038834	-5.366332
H	-0.265572	-1.346035	-5.666728
O	-0.075936	-1.983244	-3.720888
H	-0.271473	-1.117911	-3.309238
C	-2.931364	-2.453665	-3.736593
O	-3.429150	-3.553829	-3.953682
N	-3.063111	-1.835270	-2.555891
H	-2.538809	-0.968463	-2.450246
C	-3.831713	-2.356877	-1.423354
H	-3.351663	-3.298243	-1.158576
C	-3.709419	-1.430695	-0.186097
H	-3.512414	-2.056869	0.682958
H	-2.864151	-0.757935	-0.303333
C	-4.938367	-0.611738	0.164637
O	-5.871222	-1.094929	0.779021
N	-4.975358	0.645168	-0.192839
H	-5.856633	1.140548	-0.082199
H	-4.218648	1.094525	-0.690316
H	-4.873066	-2.622589	-1.718888
O	2.725659	-0.848088	-1.856145
H	1.980458	-1.222735	-1.364411
H	2.297376	2.078475	-0.653480
O	-3.916993	-5.126031	0.672283
H	-3.039779	-5.069200	0.252777
H	-4.536377	-4.814815	-0.007641
O	-5.342864	-3.698835	2.855153
H	-4.789446	-4.155346	2.208912
H	-4.935365	-2.824721	2.960647
O	8.594434	-2.248273	-0.742370
H	8.727132	-3.007603	-0.145945
H	7.797384	-1.815440	-0.333541
O	6.319508	-1.936817	6.625982
H	6.101575	-2.740449	6.109260
H	5.975477	-1.249139	6.038806
O	-3.469994	-1.663682	3.509157
H	-2.777582	-2.319583	3.696857
H	-3.055073	-0.826832	3.768624
O	6.387666	-3.101630	9.239074
H	6.919610	-3.869591	8.934422
H	6.125746	-2.704754	8.386846
O	9.072390	-2.115524	7.350693
H	9.513007	-2.822336	6.826667
H	8.242496	-1.992094	6.849655
C	1.354502	1.833749	2.290115
O	3.184644	2.388164	-0.430756
C	0.708933	2.976594	1.769163
C	-0.598105	2.916477	1.246566
C	-1.508286	4.134751	1.124359
O	-2.785445	3.796156	0.570912
P	-2.951755	3.537238	-1.051938
O	-2.417361	1.916080	-1.147119
P	-1.054407	1.508778	-1.892229
O	0.123510	2.112726	-1.103869
O	-1.179439	1.867514	-3.413099
O	-1.035098	-0.021564	-2.105716
O	-1.977991	4.394477	-1.822279
O	-4.408528	3.413307	-1.378543
C	1.510191	4.237942	1.649787
H	2.436703	1.820936	2.272976
H	0.922784	0.877047	2.007903

H	3.664825	1.591937	-0.135018
H	-1.158956	2.033508	1.552560
H	-1.728886	4.539987	2.118668
H	-1.043773	4.927422	0.529835
H	1.629166	4.689227	2.638898
H	2.496401	4.002160	1.248719
H	1.029813	4.956677	0.990919
H	-0.195187	2.563430	0.062068
Fe	-1.272400	2.373847	6.251689
Fe	0.771673	1.707698	4.314453
Fe	0.273429	0.004113	6.478200
Fe	1.525879	2.602337	6.936758
S	-1.266484	0.608516	4.767819
S	2.368063	0.785617	5.734342
S	0.355399	3.782048	5.305180
S	-0.173668	1.588994	8.208370

I4 (C2 protonation)

H	-6.251302	-7.040596	0.815265
C	-5.895057	-5.999992	0.948844
H	-6.740012	-5.390081	1.264255
H	-5.163372	-5.986993	1.759450
S	-5.168286	-5.322908	-0.603782
H	6.034155	-5.385060	-3.578887
N	6.156055	-1.963220	-3.201797
H	5.644037	-1.775582	-2.334764
C	6.480514	-3.197659	-3.712402
C	6.333906	-4.502027	-2.973400
H	5.605943	-4.364582	-2.176377
H	7.298107	-4.707267	-2.513314
N	7.180263	-1.617129	-5.126555
C	7.097777	-2.998972	-4.917496
H	7.510931	-3.767847	-5.554371
C	6.610754	-1.036676	-4.056701
H	6.518456	0.028850	-3.905326
H	-6.096011	0.252310	-5.647183
C	-5.690778	0.119000	-4.619023
H	-5.138744	-0.819613	-4.595701
H	-4.978873	0.924168	-4.438693
S	-6.932460	0.129640	-3.232119
H	7.925204	4.684734	-2.372891
N	5.432432	1.425424	-1.739859
H	5.272042	0.422983	-1.605063
C	6.533835	2.136409	-1.331051
H	7.438023	1.742741	-0.883562
N	5.038518	3.525328	-2.269098
C	6.284174	3.434850	-1.651502
C	4.559644	2.271916	-2.304116
H	3.604814	1.977154	-2.718217
C	7.260016	4.563492	-1.491091
H	7.867808	4.361603	-0.607652
H	6.709687	5.488632	-1.319542
H	2.705913	5.469846	-4.019895
C	2.211757	5.070932	-3.104840
H	2.034293	4.003338	-3.235061
H	2.892720	5.176121	-2.258578
C	0.895380	5.806574	-2.825560
H	1.017354	6.893093	-2.808180
H	0.173157	5.600216	-3.625022
C	0.208451	5.425614	-1.509667
O	-0.547746	6.281808	-0.976937
O	0.414377	4.282148	-1.020760
H	2.128856	3.612261	-0.298838
H	2.406529	6.887727	2.132722
C	2.794010	5.901173	1.800631
H	2.321017	5.104968	2.375499
H	2.544920	5.775584	0.747167
C	4.307238	5.769393	1.967429
O	4.937387	6.383394	2.817131
N	4.951859	4.931321	1.194354
H	5.946957	4.870130	1.306749
H	4.426418	4.292176	0.597559
H	-2.058435	4.411802	1.911117
C	-2.555852	3.892113	1.055795
H	-3.285167	3.200490	1.489520
O	-1.660868	3.093377	0.324584
H	-0.959123	3.639749	-0.095551
C	-3.307497	4.887969	0.166151
H	-3.943415	4.343660	-0.539161
H	-3.931028	5.551408	0.788963
H	-2.618005	5.522651	-0.393451
H	0.551899	1.690702	3.738056
C	0.999352	0.908939	3.087786
H	1.362431	0.113969	3.730598
O	2.066828	1.415207	2.326097
H	2.113388	0.873716	1.517707
C	0.014162	0.315753	2.078917
H	0.480365	-0.517653	1.555023
H	-0.872014	-0.047753	2.593918
H	-0.284358	1.067181	1.349537
H	-6.881460	1.789272	3.910875
C	-6.597599	1.118883	3.079058
H	-7.452326	0.479422	2.855113
H	-6.414431	1.741909	2.203169
S	-5.127233	0.055735	3.396397
H	7.123628	-2.710794	0.495641
C	7.092403	-2.018236	1.362659
O	7.663544	-2.296643	2.423134
N	6.399609	-0.889201	1.213441
H	5.859818	-0.835356	0.350227
C	6.171042	0.101909	2.273996

H	7.133337	0.482924	2.614806
C	5.365084	1.285023	1.726323
H	6.027508	1.912450	1.135373
H	4.565070	0.918120	1.087423
O	4.797309	2.075966	2.750356
H	3.831452	1.977628	2.669265
C	5.483264	-0.530011	3.483228
O	5.993850	-0.443575	4.600577
N	4.341352	-1.186678	3.274152
H	4.038884	-1.301470	2.308458
C	3.546691	-1.812583	4.318864
H	3.301357	-1.009229	5.011064
C	2.211205	-2.348908	3.761530
H	1.453327	-2.232498	4.531756
H	1.900695	-1.768540	2.898919
C	2.202257	-3.805159	3.359510
O	1.868891	-4.673078	4.145674
N	2.558812	-4.112522	2.141942
H	2.688785	-5.088937	1.904542
H	2.874441	-3.376293	1.500464
H	4.142010	-2.552371	4.894109
O	2.898318	3.214050	0.139767
H	2.840814	2.265720	-0.056411
H	1.446036	1.254990	-1.631651
O	1.740571	-0.973022	7.059517
H	1.918727	-0.172298	6.532342
H	2.543423	-1.509813	6.971927
O	-0.165592	-3.224812	6.582663
H	0.375801	-2.437186	6.720206
H	-0.394120	-3.220307	5.639765
O	-0.160911	8.905700	-1.091209
H	-0.674025	9.135242	-0.298763
H	-0.325423	7.923512	-1.156393
O	-6.575525	5.697885	0.600947
H	-6.207448	6.155389	1.385543
H	-5.762417	5.332934	0.218082
O	-1.136726	-2.504604	3.977331
H	-1.488092	-1.645816	4.266438
H	-1.627827	-2.692418	3.163996
O	-9.222744	5.671910	1.704188
H	-9.003476	6.600310	1.967504
H	-8.341394	5.394335	1.392760
O	-8.073154	7.770541	-0.776769
H	-7.945555	8.606821	-0.274112
H	-7.379626	7.201809	-0.391474
C	-0.667459	-1.478217	-1.188345
O	0.957664	1.764512	-2.305902
C	-0.862651	-1.701418	-2.510411
C	-1.002387	-3.095395	-3.062740
C	0.359233	-3.732961	-3.388064
O	1.077751	-4.021925	-2.208144
P	2.644406	-3.447195	-2.035202
O	2.231086	-1.909815	-1.489113
P	3.154685	-1.011432	-0.507254
O	2.473940	0.336083	-0.330989
O	4.628994	-1.065815	-1.012680
O	3.417581	-1.817117	0.799635
O	3.234614	-3.263228	-3.407700
O	3.308141	-4.217168	-0.928151
C	-0.797634	-0.586818	-3.508921
H	-0.311289	-0.506304	-0.853001
H	-0.514315	-2.311726	-0.515713
H	0.707845	2.601351	-1.880694
H	-1.601353	-3.078675	-3.977630
H	0.143707	-4.672673	-3.914534
H	0.944913	-3.112114	-4.071874
H	-1.737765	-0.514584	-4.068897
H	-0.540805	0.372734	-3.061147
H	-0.014434	-0.820008	-4.236592
H	-1.522201	-3.753491	-2.366356
Fe	-4.816756	-3.130376	-0.179853
Fe	-2.904707	-1.075039	-0.907987
Fe	-4.834592	-0.724229	1.262508
Fe	-5.696481	-0.737848	-1.488963
S	-3.149935	-2.292693	1.204419
S	-4.314921	0.789628	-0.409462
S	-4.224195	-2.321658	-2.314361
S	-6.706851	-1.813224	0.356523

I5 (C2 protonation)

H	-4.720473	3.228190	8.833369
C	-3.914384	2.614388	8.378171
H	-3.351529	2.137783	9.181093
H	-4.375937	1.822270	7.785691
S	-2.778389	3.632089	7.347458
H	-4.378866	6.049170	-4.041954
N	-1.519585	4.232412	-4.637198
H	-1.595073	3.409604	-4.031006
C	-2.475822	5.198395	-4.844365
C	-3.909981	5.105677	-4.396069
H	-3.974833	4.365019	-3.602126
H	-4.477923	4.734262	-5.247011
N	-0.636034	5.740722	-5.987723
C	-1.933448	6.151877	-5.662488
H	-2.442089	7.024925	-6.045439
C	-0.440254	4.570397	-5.356695
H	0.466665	3.985841	-5.402837
H	4.239640	6.009571	6.600169
C	3.577352	5.134539	6.412664
H	2.605835	5.507332	6.090364
H	4.001516	4.577809	5.578061

S	3.336829	3.960647	7.832182
H	3.551618	0.603122	-7.564128
N	1.041336	1.506035	-4.339442
H	0.149251	1.810211	-3.936846
C	1.223141	0.844472	-5.530130
H	0.467173	0.604569	-6.267819
N	3.197322	1.074309	-4.480656
C	2.554348	0.570163	-5.609707
C	2.230968	1.634537	-3.735827
H	2.374575	2.102481	-2.771508
C	3.231422	-0.103524	-6.768571
H	2.531935	-0.825571	-7.192925
H	4.098815	-0.651864	-6.400831
H	6.204636	1.984371	-2.904463
C	5.607134	1.362037	-2.202817
H	4.771491	1.948976	-1.824230
H	5.181232	0.517651	-2.746704
C	6.478028	0.866260	-1.041687
H	7.377161	0.354899	-1.389524
H	6.818190	1.720144	-0.443198
C	5.745990	-0.088706	-0.096314
O	6.376767	-1.081030	0.357849
O	4.541699	0.168438	0.173184
H	3.108443	-0.293577	-1.104914
H	4.931861	-4.177773	-1.915955
C	4.115889	-3.441533	-2.091296
H	3.288862	-3.635496	-1.408470
H	4.503758	-2.440928	-1.898410
C	3.561277	-3.486002	-3.511580
O	3.585814	-4.497095	-4.202878
N	2.997821	-2.401483	-3.985511
H	2.633600	-2.446311	-4.918771
H	2.830165	-1.599082	-3.379414
H	3.896580	-2.665733	2.763486
C	3.904065	-1.626752	3.164592
H	3.263404	-1.625287	4.051350
O	3.292972	-0.730094	2.253621
H	3.878425	-0.501236	1.466874
C	5.302013	-1.167450	3.561105
H	5.238733	-0.233378	4.119380
H	5.785978	-1.911066	4.211315
H	5.937634	-1.030104	2.685062
H	0.106382	-3.375714	1.124163
C	-0.458198	-2.481576	0.783569
H	-1.507802	-2.752352	0.715434
O	-0.005786	-2.024428	-0.467959
H	-0.150496	-1.060098	-0.485992
C	-0.321536	-1.295820	1.734030
H	-1.009111	-0.506289	1.435356
H	-0.555007	-1.602698	2.751013
H	0.696130	-0.916159	1.705829

H	1.818511	-3.496166	8.730164
C	1.527239	-2.451306	8.519299
H	1.217043	-1.976957	9.450744
H	2.415893	-1.933288	8.163412
S	0.180188	-2.292740	7.267688
H	-3.897751	1.189504	-5.001107
C	-3.613281	0.116814	-4.979884
O	-4.389831	-0.756465	-5.383478
N	-2.412060	-0.184225	-4.485364
H	-1.923084	0.595096	-4.045135
C	-1.904248	-1.551340	-4.301540
H	-1.877594	-2.053631	-5.268136
C	-0.474348	-1.509213	-3.750482
H	0.203860	-1.255939	-4.561132
H	-0.399408	-0.746963	-2.977490
O	-0.073208	-2.747762	-3.202903
H	0.025796	-2.604653	-2.244250
C	-2.824660	-2.366281	-3.393006
O	-3.310795	-3.428998	-3.783522
N	-3.084000	-1.868090	-2.184923
H	-2.734195	-0.931876	-1.991044
C	-3.900256	-2.513084	-1.171655
H	-3.425532	-3.475143	-0.985972
C	-3.855718	-1.722725	0.153388
H	-3.886481	-2.432280	0.977286
H	-2.927634	-1.163180	0.231225
C	-5.001652	-0.762770	0.368351
O	-6.000860	-1.108010	0.970192
N	-4.899616	0.447663	-0.110781
H	-5.707263	1.060512	-0.104783
H	-4.067385	0.706000	-0.652496
H	-4.920943	-2.732157	-1.551916
O	2.391392	-0.627203	-1.669450
H	1.605723	-0.108377	-1.423386
H	1.473279	1.473704	1.775489
O	-3.825767	-5.245265	0.894610
H	-2.948250	-5.123638	0.487541
H	-4.456256	-4.964888	0.214680
O	-5.121857	-3.846933	3.166262
H	-4.627065	-4.323439	2.488100
H	-4.659721	-2.999851	3.270954
O	8.486387	-1.998175	-1.026606
H	8.669475	-2.848885	-0.606583
H	7.741995	-1.621750	-0.478267
O	4.821515	-2.141197	6.769741
H	5.159442	-2.745195	6.079523
H	4.277169	-1.485095	6.304917
O	-3.165863	-1.797546	3.557999
H	-2.455706	-2.407964	3.821070
H	-2.923775	-0.978014	4.014546
O	5.555746	-3.903606	8.920439

H	6.511908	-3.859860	8.688593
H	5.186392	-3.256096	8.293093
O	7.350248	-1.470579	8.024333
H	8.110057	-1.862122	7.536459
H	6.620765	-1.568002	7.384786
C	-1.067164	2.593815	2.524603
O	1.738397	1.281388	2.682226
C	-0.594746	3.802928	2.185947
C	-1.354903	5.039051	2.571923
C	-2.215488	5.514647	1.376026
O	-2.977747	4.466364	0.813216
P	-2.731599	3.980925	-0.779968
O	-1.541513	2.831444	-0.508386
P	-1.241769	1.543871	-1.453415
O	-0.031960	0.825357	-0.886679
O	-1.252557	1.989601	-2.949632
O	-2.556398	0.713115	-1.560771
O	-2.055289	5.107691	-1.520641
O	-3.982397	3.292738	-1.249324
C	0.574033	3.999534	1.266421
H	-0.741211	1.706033	1.995784
H	-1.981013	2.507457	3.093044
H	2.375512	0.503126	2.639812
H	-0.689905	5.861704	2.857006
H	-2.903989	6.280113	1.763251
H	-1.590607	5.989999	0.614510
H	1.444117	4.368213	1.825643
H	0.834766	3.090867	0.728447
H	0.327142	4.746889	0.510629
H	-2.000452	4.846637	3.430044
Fe	-1.057153	2.307235	6.680136
Fe	0.593772	1.830963	4.283264
Fe	0.416552	-0.079784	6.741592
Fe	1.724090	2.483165	7.050952
S	-1.246540	0.482276	5.249233
S	2.408747	0.612427	5.832920
S	0.417704	3.748777	5.585146
S	0.136198	1.429490	8.505446

TS3 (C4 protonation)

H	-7.195606	-6.465688	0.340671
C	-6.677355	-5.508200	0.543991
H	-7.416936	-4.783159	0.879223
H	-5.976257	-5.661500	1.367821
S	-5.801574	-4.884379	-0.954825
H	5.082651	-5.670505	-3.933319
N	5.656014	-2.338384	-3.351742
H	5.208301	-2.127941	-2.456298
C	5.838384	-3.575144	-3.925258
C	5.524249	-4.893798	-3.271323
H	4.852535	-4.715556	-2.437081
H	6.460683	-5.275820	-2.866901
N	6.736592	-2.020084	-5.251732
C	6.496014	-3.390606	-5.111236
H	6.826451	-4.169467	-5.783517
C	6.218738	-1.429575	-4.161830
H	6.254188	-0.369486	-3.957792
H	-5.940402	1.112703	-5.718461
C	-5.541694	0.823821	-4.719420
H	-5.118673	-0.177123	-4.805079
H	-4.723632	1.503600	-4.482663
S	-6.757082	0.856908	-3.310509
H	8.642512	3.906666	-2.059042
N	5.756887	1.003035	-1.499106
H	5.468019	0.024357	-1.413674
C	6.929281	1.562654	-1.053814
H	7.778009	1.048624	-0.619190
N	5.634434	3.151916	-1.980527
C	6.848611	2.889439	-1.347669
C	5.012524	1.966772	-2.060004
H	4.048081	1.800172	-2.515280
C	7.968631	3.871170	-1.174994
H	8.541956	3.574652	-0.294937
H	7.552858	4.862607	-0.988894
H	3.784310	5.793508	-3.502258
C	3.274835	5.458334	-2.577037
H	3.100157	4.389476	-2.661749
H	3.927632	5.630861	-1.717600
C	1.933505	6.188957	-2.423087
H	2.065903	7.266295	-2.306879
H	1.331905	6.037896	-3.328805
C	1.081809	5.671496	-1.261158
O	0.494302	6.499190	-0.521500
O	1.003940	4.417672	-1.104561
H	2.473560	3.514518	-0.214349
H	3.131273	6.335266	2.661082
C	3.371341	5.329602	2.255701
H	2.805464	4.567582	2.791315
H	3.088489	5.312973	1.203874
C	4.857034	4.995466	2.372981
O	5.544199	5.384252	3.307828
N	5.406282	4.239319	1.454191
H	6.394172	4.076752	1.537635
H	4.833386	3.768812	0.757342
H	-1.386943	4.350615	2.059958
C	-1.822328	3.910640	1.129477
H	-2.621563	3.233487	1.444835
O	-0.884732	3.113957	0.441909
H	-0.252947	3.681651	-0.053670
C	-2.419587	4.989430	0.222465
H	-3.045042	4.524676	-0.543819
H	-3.029541	5.694966	0.806490

H	-1.636910	5.580076	-0.255927
H	0.788571	1.295755	3.873602
C	1.158390	0.496064	3.198080
H	1.539933	-0.315321	3.807645
O	2.178811	0.935772	2.341368
H	2.387159	0.179668	1.759823
C	0.062907	-0.030825	2.272929
H	0.468472	-0.804870	1.626085
H	-0.738606	-0.462974	2.862080
H	-0.343145	0.777064	1.668625
H	-6.639318	2.212819	3.985873
C	-6.445605	1.560437	3.114135
H	-7.388572	1.085115	2.841073
H	-6.151768	2.205270	2.284639
S	-5.174346	0.249726	3.364763
H	6.641275	-3.459576	0.300336
C	6.709593	-2.807362	1.192810
O	7.315675	-3.176087	2.203163
N	6.074510	-1.637437	1.144507
H	5.511303	-1.468858	0.312063
C	5.931810	-0.746420	2.298454
H	6.924155	-0.545551	2.702258
C	5.345333	0.599280	1.862939
H	6.123784	1.169099	1.360161
H	4.529120	0.443395	1.161706
O	4.874691	1.356981	2.956395
H	3.914687	1.441254	2.826690
C	5.134614	-1.422795	3.419395
O	5.626389	-1.481311	4.548018
N	3.942733	-1.973308	3.139476
H	3.617456	-1.902466	2.177261
C	3.134546	-2.646714	4.163007
H	2.962233	-1.886101	4.921407
C	1.735130	-3.101908	3.700453
H	1.217638	-3.512609	4.564395
H	1.149945	-2.263247	3.357395
C	1.672161	-4.151015	2.619911
O	1.394630	-5.309063	2.867290
N	1.846570	-3.752193	1.392394
H	1.895697	-4.442741	0.655784
H	2.161542	-2.807794	1.188949
H	3.686060	-3.452466	4.688287
O	3.100301	2.891985	0.193749
H	2.865190	2.047197	-0.218135
H	1.937927	1.984921	-2.868888
O	1.521728	-1.613726	6.989060
H	1.836832	-0.791868	6.568058
H	2.267721	-2.227270	6.907844
O	-0.609012	-3.642750	6.445965
H	-0.045701	-2.878500	6.622707
H	-0.919753	-3.534823	5.532328
O	1.339138	9.056375	-0.533392
H	0.812528	9.400672	0.210530
H	1.004576	8.117739	-0.580819
O	-5.594115	6.202376	0.672969
H	-5.022508	6.303099	1.462126
H	-4.970386	5.797213	0.053709
O	-1.569566	-2.819421	3.890928
H	-1.808813	-1.913219	4.151030
H	-2.074725	-2.959193	3.075033
O	-8.113026	6.361052	2.056139
H	-7.838190	7.174227	2.534253
H	-7.267089	6.075023	1.665360
O	-6.719092	8.698371	-0.036853
H	-6.215171	9.422139	0.399857
H	-6.119464	7.939299	0.106074
C	-0.229152	0.620672	-1.574641
O	1.588415	2.757753	-3.321650
C	-0.634644	-0.651655	-2.070410
C	-0.954693	-1.737036	-1.237755
C	-0.890868	-3.185399	-1.702453
O	0.323047	-3.853786	-1.323355
P	1.802009	-3.624741	-2.025050
O	1.943982	-1.961821	-1.721662
P	2.861520	-0.988954	-0.871380
O	2.328038	0.420293	-1.238016
O	4.361623	-1.306160	-1.082069
O	2.831315	-1.286138	0.666500
O	1.785707	-3.687819	-3.529468
O	2.760502	-4.398759	-1.162217
C	-0.662202	-0.850529	-3.558812
H	-0.392544	1.455992	-2.246867
H	-0.493524	0.859568	-0.548002
H	1.287056	3.336216	-2.593485
H	-0.665326	-1.619234	-0.193247
H	-1.677319	-3.765909	-1.219833
H	-1.047214	-3.259203	-2.781956
H	-1.610229	-1.302374	-3.855504
H	-0.550751	0.098479	-4.083360
H	0.135528	-1.533505	-3.862567
H	1.061612	0.510057	-1.466502
Fe	-5.201894	-2.773715	-0.407116
Fe	-2.970941	-1.073366	-0.809076
Fe	-4.975203	-0.430418	1.167711
Fe	-5.598722	-0.262127	-1.647652
S	-3.540283	-2.293625	1.141125
S	-4.056790	0.993567	-0.453975
S	-4.281791	-2.020328	-2.440131
S	-6.910273	-1.225463	0.073394

I5 (C4 protonation)

H	-6.926974	-6.464507	0.689866

C	-6.426292	-5.483438	0.814399
H	-7.177503	-4.739953	1.073303
H	-5.730771	-5.553785	1.653280
S	-5.549440	-4.966724	-0.722709
H	5.282113	-5.786148	-3.724521
N	5.712002	-2.423598	-3.231700
H	5.246711	-2.213650	-2.343610
C	5.958949	-3.664044	-3.772674
C	5.706613	-4.979447	-3.087713
H	5.042430	-4.808962	-2.246377
H	6.662720	-5.317877	-2.690493
N	6.784080	-2.102895	-5.138432
C	6.610611	-3.479084	-4.961675
H	6.978146	-4.258743	-5.613521
C	6.231107	-1.510357	-4.066187
H	6.207293	-0.444410	-3.894329
H	-6.099844	0.723012	-5.760436
C	-5.719683	0.473364	-4.744159
H	-5.274066	-0.519899	-4.786986
H	-4.922671	1.179356	-4.509674
S	-6.961868	0.522843	-3.361241
H	8.638103	3.910528	-2.177629
N	5.773612	0.985499	-1.533691
H	5.476307	0.012379	-1.415124
C	6.952469	1.552785	-1.112945
H	7.804350	1.045955	-0.675997
N	5.647307	3.127526	-2.052341
C	6.866988	2.875379	-1.427605
C	5.025938	1.940994	-2.105794
H	4.059755	1.767750	-2.553848
C	7.981630	3.867123	-1.280913
H	8.576187	3.579363	-0.412192
H	7.558080	4.854332	-1.089012
H	3.688008	5.615926	-3.694149
C	3.174179	5.299014	-2.764259
H	2.970086	4.232591	-2.838498
H	3.836881	5.455991	-1.909983
C	1.861907	6.079290	-2.611972
H	2.032264	7.152587	-2.500372
H	1.252683	5.955520	-3.517709
C	0.973703	5.620674	-1.454356
O	0.292024	6.489478	-0.846857
O	0.950847	4.391439	-1.171967
H	2.445633	3.475827	-0.220981
H	3.023820	6.531992	2.382140
C	3.317199	5.516877	2.034119
H	2.768764	4.757941	2.591460
H	3.064154	5.436097	0.977472
C	4.806277	5.231285	2.204995
O	5.464194	5.708251	3.118925
N	5.387086	4.411699	1.363166

H	6.376589	4.274507	1.469771
H	4.828008	3.860274	0.715601
H	-1.557935	4.448139	1.753236
C	-2.039179	4.048572	0.827523
H	-2.840343	3.375783	1.154763
O	-1.160052	3.248371	0.083532
H	-0.421556	3.779982	-0.300044
C	-2.668173	5.160742	-0.013148
H	-3.259134	4.726869	-0.822772
H	-3.334401	5.786069	0.604605
H	-1.908894	5.818013	-0.442208
H	0.646462	1.513496	3.833594
C	0.916085	0.630819	3.215099
H	1.002505	-0.208688	3.898774
O	2.130101	0.806044	2.522739
H	2.233315	0.042929	1.921961
C	-0.111098	0.289679	2.136587
H	0.140922	-0.669101	1.688285
H	-1.106725	0.226510	2.570483
H	-0.103952	1.058525	1.367327
H	-6.543983	2.367665	3.895108
C	-6.338919	1.688404	3.047089
H	-7.282951	1.207473	2.788657
H	-6.042242	2.306690	2.200344
S	-5.091805	0.369507	3.322785
H	6.840663	-3.298318	0.406570
C	6.918110	-2.599031	1.262352
O	7.547016	-2.910283	2.277068
N	6.260896	-1.443932	1.169547
H	5.679626	-1.344695	0.338979
C	6.055045	-0.528175	2.294325
H	7.026230	-0.237449	2.694288
C	5.342252	0.746398	1.827444
H	6.050774	1.361312	1.277384
H	4.517542	0.489287	1.165999
O	4.838892	1.504244	2.908562
H	3.870534	1.408016	2.872603
C	5.297517	-1.232525	3.419661
O	5.795934	-1.284642	4.546101
N	4.113914	-1.792547	3.139520
H	3.776132	-1.746963	2.178156
C	3.266487	-2.381668	4.172749
H	3.106220	-1.576175	4.887078
C	1.871616	-2.768341	3.654189
H	1.205761	-2.881624	4.505630
H	1.484394	-1.981600	3.023690
C	1.782705	-4.037526	2.851383
O	1.505624	-5.105212	3.364997
N	1.953339	-3.926855	1.565686
H	1.995380	-4.742853	0.982575
H	2.239728	-3.028995	1.172684

H	3.773922	-3.187449	4.743727
O	3.081270	2.909040	0.246060
H	2.947336	2.034548	-0.164298
H	1.973257	1.648639	-2.530792
O	1.535744	-1.375070	6.906046
H	1.804438	-0.562668	6.437582
H	2.287800	-1.977753	6.798820
O	-0.632498	-3.405083	6.402270
H	-0.028979	-2.683301	6.622156
H	-0.901669	-3.233359	5.484646
O	1.019353	9.045473	-0.858610
H	0.487222	9.348077	-0.107843
H	0.736578	8.088398	-0.934595
O	-5.767499	5.231135	1.613176
H	-5.282664	5.977728	2.020936
H	-5.082625	4.817951	1.068763
O	-1.413877	-2.354376	3.876213
H	-1.680634	-1.472556	4.189656
H	-1.938030	-2.482059	3.070685
O	-8.694985	5.968043	2.146410
H	-8.460409	6.924487	2.227611
H	-7.819157	5.605590	1.930007
O	-7.143233	6.978204	-0.264908
H	-6.945765	7.940190	-0.177980
H	-6.430567	6.585343	0.267149
C	-0.840343	0.752534	-1.553235
O	1.598460	2.315181	-3.132956
C	-0.879140	-0.649671	-2.117483
C	-0.772328	-1.746803	-1.322214
C	-0.642369	-3.189771	-1.763792
O	0.519847	-3.809619	-1.235122
P	2.024692	-3.566889	-1.934605
O	2.087735	-1.902711	-1.778521
P	2.950259	-0.954878	-0.789698
O	2.557255	0.471321	-1.120239
O	4.449128	-1.365803	-0.940732
O	2.777950	-1.390404	0.703346
O	1.986876	-3.813589	-3.420764
O	2.995058	-4.248926	-1.011094
C	-0.834303	-0.706351	-3.619713
H	-1.237857	0.855871	-0.541376
H	0.209162	1.047025	-1.482808
H	1.278460	3.015925	-2.539463
H	-0.601390	-1.601973	-0.261515
H	-1.489687	-3.750618	-1.358774
H	-0.679587	-3.279078	-2.854149
H	-1.838064	-0.630265	-4.051176
H	-0.268921	0.155292	-3.978161
H	-0.358412	-1.614607	-3.982125
H	-1.353712	1.471388	-2.197959
Fe	-4.994723	-2.803111	-0.360959
Fe	-2.960202	-0.837169	-1.094526
Fe	-4.935981	-0.443550	1.180742
Fe	-5.779303	-0.416707	-1.611908
S	-3.306273	-2.063821	1.047572
S	-4.385848	1.057712	-0.481111
S	-4.323820	-2.047893	-2.474244
S	-6.835227	-1.475947	0.249537

I5 (C4 protonation)

H	-6.158300	-6.650757	0.766088
C	-5.737310	-5.626688	0.837044
H	-6.542602	-4.940573	1.094992
H	-5.012520	-5.606735	1.652998
S	-4.945636	-5.064266	-0.729143
H	5.767999	-5.711172	-3.918897
N	5.976561	-2.302905	-3.305493
H	5.641131	-2.111626	-2.357693
C	6.197233	-3.515648	-3.924014
C	6.106939	-4.872517	-3.266028
H	5.434587	-4.798058	-2.415581
H	7.097065	-5.106572	-2.879601
N	6.725892	-1.864425	-5.337109
C	6.644025	-3.253391	-5.192474
H	6.941120	-3.986737	-5.928648
C	6.321911	-1.337652	-4.169274
H	6.283065	-0.281373	-3.943292
H	-6.618155	0.233487	-5.746528
C	-6.233256	0.001436	-4.727818
H	-5.758486	-0.977904	-4.764106
H	-5.448538	0.724264	-4.509508
S	-7.444104	0.034027	-3.319125
H	8.260689	4.455471	-2.611664
N	5.462654	1.615690	-1.536989
H	5.233055	0.626359	-1.396738
C	6.701943	2.181319	-1.375771
H	7.626641	1.674590	-1.127223
N	5.216564	3.773343	-1.957292
C	6.549916	3.505909	-1.643129
C	4.594017	2.584978	-1.865518
H	3.538757	2.416228	-2.027349
C	7.671735	4.497703	-1.671880
H	8.326399	4.287840	-0.823873
H	7.266440	5.502276	-1.544132
H	2.959123	5.517863	-4.064194
C	2.419001	5.184627	-3.153026
H	2.264243	4.107853	-3.202779
H	3.035588	5.389503	-2.276354
C	1.061112	5.896061	-3.040839
H	1.167698	6.982590	-3.033912
H	0.437022	5.637501	-3.905469
C	0.287495	5.487140	-1.787258

O	-0.180614	6.386926	-1.041868
O	0.180554	4.251083	-1.559053
H	1.520408	3.362498	-0.416676
H	2.955839	6.689878	2.019852
C	3.353564	5.703911	1.688829
H	2.898690	4.903290	2.272349
H	3.122548	5.555259	0.633769
C	4.865163	5.656115	1.897472
O	5.363760	5.888057	2.989714
N	5.655365	5.400126	0.881484
H	6.641436	5.583753	1.039104
H	5.314147	5.066927	-0.007531
H	-1.666936	4.439770	1.821430
C	-2.244633	3.989973	0.981849
H	-2.965985	3.296517	1.427929
O	-1.400551	3.200251	0.172504
H	-0.811089	3.732474	-0.455547
C	-3.003050	5.020721	0.157351
H	-3.618169	4.520223	-0.592783
H	-3.669423	5.610836	0.802403
H	-2.319168	5.707867	-0.343836
H	0.802046	1.581306	3.738157
C	1.131035	0.699961	3.148693
H	1.179766	-0.120537	3.858034
O	2.390586	0.865330	2.532700
H	2.510009	0.112112	1.929064
C	0.168935	0.308789	2.027801
H	0.455464	-0.659684	1.618248
H	-0.846666	0.242093	2.417456
H	0.203366	1.056103	1.237846
H	-6.898239	1.805260	4.098364
C	-6.727459	1.102259	3.265103
H	-7.631704	0.506751	3.132507
H	-6.593904	1.696294	2.363384
S	-5.284152	-0.023800	3.470836
H	7.210000	-3.017900	0.306680
C	7.308283	-2.318835	1.164562
O	7.923723	-2.644943	2.184007
N	6.682111	-1.146276	1.066169
H	6.138486	-1.025996	0.212958
C	6.470028	-0.202765	2.169277
H	7.437437	0.159678	2.515877
C	5.657458	1.007630	1.687499
H	6.317808	1.662205	1.122443
H	4.850756	0.674471	1.038164
O	5.089530	1.749586	2.748783
H	4.135275	1.551231	2.751398
C	5.796645	-0.894976	3.351044
O	6.344274	-0.891201	4.452749
N	4.624302	-1.501196	3.148145
H	4.254783	-1.525917	2.198477
C	3.844487	-2.091601	4.227911
H	3.739850	-1.299422	4.968179
C	2.417920	-2.449397	3.782252
H	1.751749	-2.389684	4.636989
H	2.085440	-1.743181	3.036258
C	2.231811	-3.811419	3.179504
O	1.947814	-4.786156	3.851914
N	2.357728	-3.896788	1.889139
H	2.369285	-4.833192	1.510938
H	2.669764	-3.095925	1.336900
H	4.387872	-2.912962	4.736549
O	2.048344	2.973217	0.296510
H	2.291215	2.083585	-0.017565
H	-0.965965	0.617555	-0.947938
O	1.940969	-1.118325	6.937721
H	2.145860	-0.313008	6.426090
H	2.722424	-1.679709	6.834390
O	-0.115805	-3.264963	6.390663
H	0.469642	-2.523357	6.590478
H	-0.314866	-3.180834	5.444400
O	0.560619	8.951514	-1.110213
H	0.052214	9.239306	-0.334908
H	0.290037	7.992663	-1.167833
O	-6.192034	4.430989	1.665035
H	-5.557963	5.106052	1.980338
H	-5.660842	3.819476	1.133113
O	-0.910950	-2.314160	3.789515
H	-1.341829	-1.499703	4.100728
H	-1.349062	-2.500556	2.948698
O	-9.010465	5.346029	2.331183
H	-8.765989	6.299821	2.412955
H	-8.139549	4.967498	2.116070
O	-7.453586	6.349631	-0.120314
H	-7.247435	7.305959	-0.013390
H	-6.762197	5.923811	0.413796
C	0.040174	0.443751	-2.721297
O	-1.800745	0.831174	-0.511516
C	-0.325098	-1.015851	-2.740445
C	-0.060883	-1.942785	-1.779664
C	-0.058539	-3.440672	-1.887319
O	1.080711	-3.980587	-1.217765
P	2.611903	-3.698005	-1.831973
O	2.641264	-2.027296	-1.753712
P	3.323315	-0.978285	-0.728371
O	2.645267	0.364007	-0.892964
O	4.855899	-1.051357	-0.995527
O	3.307577	-1.540149	0.730867
O	2.641336	-3.987081	-3.311574
O	3.558213	-4.278934	-0.817356
C	-0.805840	-1.437554	-4.094439
H	0.531537	0.763472	-1.796627

H	0.820301	0.600164	-3.470679
H	-1.730278	1.802326	-0.210361
H	0.305067	-1.586303	-0.824202
H	-0.932230	-3.866791	-1.382048
H	-0.077033	-3.770718	-2.931360
H	-1.703765	-0.878097	-4.382336
H	-0.027326	-1.212825	-4.833679
H	-1.047040	-2.496134	-4.153953
H	-0.794972	1.096079	-3.012798
Fe	-4.780888	-2.819483	-0.457001
Fe	-3.347904	-0.328161	-0.856925
Fe	-5.160838	-0.651929	1.269827
Fe	-6.142913	-0.532849	-1.489966
S	-3.265487	-1.925356	1.034975
S	-5.133592	1.162179	-0.193372
S	-4.279830	-1.702490	-2.428303
S	-6.840161	-1.988197	0.310335

1. T. Grawert, I. Span, W. Eisenreich, F. Rohdich, J. Eppinger, A. Bacher and M. Groll, *P. Natl. Acad. Sci. U.S.A.*, 2010, **107**, 1077-1081.
2. L. Noodleman, *J. Chem. Phys.*, 1981, **74**, 5737-5743.
3. L. Noodleman, C. Y. Peng, D. A. Case and J. M. Mouesca, *Coord. Chem. Rev.*, 1995, **144**, 199-244.
4. A. D. Becke, *J. Chem. Phys.*, 1993, **98**, 5648-5652.
5. C. T. Lee, W. T. Yang and R. G. Parr, *Phys. Rev. B*, 1988, **37**, 785-789.
6. S. H. Vosko, L. Wilk and M. Nusair, *Can. J. Phys.*, 1980, **58**, 1200-1211.
7. P. J. Stephens, F. J. Devlin, C. F. Chabalowski and M. J. Frisch, *J. Phys. Chem.*, 1994, **98**, 11623-11627.
8. F. Weigend and R. Ahlrichs, *Phys. Chem. Chem. Phys.*, 2005, **7**, 3297-3305.
9. F. Weigend, *Phys. Chem. Chem. Phys.*, 2006, **8**, 1057-1065.
10. R. D. Gaussian 09, M. J. Frisch, G. W. Trucks, H. B. Schlegel, G. E. Scuseria, M. A. Robb, J. R. Cheeseman, G. Scalmani, V. Barone, B. Mennucci, G. A. Petersson, H. Nakatsuji, M. Caricato, X. Li, H. P. Hratchian, A. F. Izmaylov, J. Bloino, G. Zheng, J. L. Sonnenberg, M. Hada, M. Ehara, K. Toyota, R. Fukuda, J. Hasegawa, M. Ishida, T. Nakajima, Y. Honda, O. Kitao, H. Nakai, T. Vreven, J. A. Montgomery, Jr., J. E. Peralta, F. Ogliaro, M. Bearpark, J. J. Heyd, E. Brothers, K. N. Kudin, V. N. Staroverov, R. Kobayashi, J. Normand, K. Raghavachari, A. Rendell, J. C. Burant, S. S. Iyengar, J. Tomasi, M. Cossi, N. Rega, J. M. Millam, M. Klene, J. E. Knox, J. B. Cross, V. Bakken, C. Adamo, J. Jaramillo, R. Gomperts, R. E. Stratmann, O. Yazyev, A. J. Austin, R. Cammi, C. Pomelli, J. W. Ochterski, R. L. Martin, K. Morokuma, V. G. Zakrzewski, G. A. Voth, P. Salvador, J. J. Dannenberg, S. Dapprich, A. D. Daniels, Ö. Farkas, J. B. Foresman, J. V. Ortiz, J. Cioslowski, and D. J. Fox, Gaussian, Inc., Wallingford CT, 2009.
11. R. K. Dennington, Todd; Millam, John. , *Semichem Inc., Shawnee Mission, KS*, 2009.
12. C. Greco, M. Bruschi, P. Fantucci, U. Ryde and L. De Gioia, *J. Am. Chem. Soc.*, 2011, **133**, 18742-18749.
13. B. R. Brooks, C. L. Brooks, A. D. Mackerell, L. Nilsson, R. J. Petrella, B. Roux, Y. Won, G. Archontis, C. Bartels, S. Boresch, A. Caflisch, L. Caves, Q. Cui, A. R. Dinner, M. Feig, S. Fischer, J. Gao, M. Hodoscek, W. Im, K. Kuczera, T. Lazaridis, J. Ma, V. Ovchinnikov, E. Paci, R. W. Pastor, C. B. Post, J. Z. Pu, M. Schaefer, B. Tidor, R. M. Venable, H. L. Woodcock, X. Wu, W. Yang, D. M. York and M. Karplus, *J. Comput. Chem.*, 2009, **30**, 1545-1614.
14. J. W. Ponder and D. A. Case, *Adv. Protein Chem.*, 2003, **66**, 27-85.
15. A. W. Sousa Da Silva, W. F. Vranken, *BMC Res. Notes* 2012, **5**, 367.
16. J. M. Wang, R. M. Wolf, J. W. Caldwell, P. A. Kollman and D. A. Case, *J. Comput. Chem.*, 2004, **25**, 1157-1174.
17. J. M. Wang, W. Wang, P. A. Kollman and D. A. Case, *J. Mol. Graph. Model.*, 2006, **25**, 247-260.
18. J. E. D. Glendening, K. Badenhoop, A. E. Reed, J. E. Carpenter, J. A. Bohmann, C. M. Morales, C. R. Landis, and F. Weinhold *Theoretical Chemistry Institute, University of Wisconsin, Madison* 2013.
19. G. Kresse and J. Furthmuller, *Phys. Rev. B.*, 1996, **54**, 11169-11186.
20. G. Kresse and J. Hafner, *Phys. Rev. B*, 1993, **47**, 558-561.
21. G. Kresse and J. Hafner, *Phys. Rev. B*, 1994, **49**, 14251-14269.
22. G. Kresse and J. Hafner, *Mater. Sci.*, 1996, **6**, 15-50.
23. M. Cococcioni and S. de Gironcoli, *Phys. Rev. B*, 2005, **71**, 035105.
24. P. E. Blochl, *Phys. Rev. B.*, 1994, **50**, 17953-17979.
25. G. Kresse and D. Joubert, *Phys. Rev. B*, 1999, **59**, 1758-1775.
